# Synthesis of Indenones via Persulfate Promoted Radical Alkylation/Cyclization of Biaryl Ynones with 1,4-Dihydropyridines

**DOI:** 10.3390/molecules29020458

**Published:** 2024-01-17

**Authors:** Wanwan Wang, Lei Yu

**Affiliations:** Jiangsu Key Laboratory of Chiral Pharmaceuticals Biosynthesis, Taizhou University, Taizhou 225300, China; yulei2637@163.com

**Keywords:** alkylation, indenones, radical, cyclization, biaryl ynones

## Abstract

The oxidative radical cascade cyclization of alkynes has emerged as a versatile strategy for the efficient construction of diverse structural units and complex molecules in organic chemistry. This work reports an alkyl radical initiated 5-*exo*-trig cyclization of biaryl ynones with 1,4-dihydropyridines to selectively synthesize indenones.

## 1. Introduction

Indenones are attracting increasing attention in organic synthesis, as these derivatives are widely used in biological molecules, pharmaceuticals and functional materials [1,2,3]. As a result, considerable effort has been dedicated to developing efficient and novel methods for accessing functionalized indenones [4,5]. The radical cascade reaction provides an efficient method for rapidly increasing the complexity of molecules to obtain indenones [4].

**Scheme 1:**
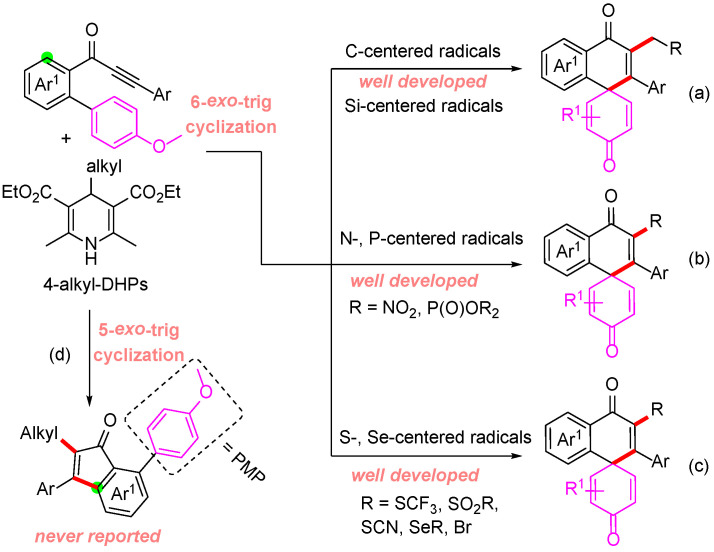
Previous radical cascade reactions of biaryl ynone (**a**–**c**) and our work (**d**). PMP: *p*-methoxyphenyl.

Alkynes are a diverse functional group widely present in organics. The radical induced cascade reactions of alkynes are a useful and efficient method for rapidly accessing complex molecules, and they have therefore attracted considerable attention [6,7,8,9,10]. The direct difunctionalization of alkyne feedstocks has developed rapidly due to its atom and step economy. Therefore, numerous methods have been established to synthesize functionalized molecules in recent years. In addition, radical cascade reactions involving the 1,2-difunctionalization of alkyne have become a versatile tool in contemporary organic chemistry [11,12,13,14]. To date, the cyclization of alkynes toward the synthesis of cyclic compounds has been effectively investigated [15,16,17,18,19,20,21,22,23,24,25,26]. Recently, the radical cascade cyclization of biaryl ynones has been demonstrated to be a useful approach to construct six-membered spiro[5,5]trienones [27,28,29,30,31,32,33,34,35,36,37,38,39,40,41,42] with the development of different types of radical precursors (Scheme 1a–c). For example, Chen and Zhou reported an iron-catalyzed cascade silyl radical addition/6-*exo*-trig cyclization/dearomatization of biaryl ynones with silane, affording silylated spiro[5.5]trienones in good yields [35]. In addition, several groups have demonstrated some elegant examples based on C-, N-, P-, S-, Si-, Se-centered radicals-induced cascade cyclization of biaryl ynones in the presence of transition-metal catalysts, oxidants or photocatalysts [27,28,29,30,31,32,33,34,35,36,37,38,39,40,41,42]. For example, Duan and Yang reported the preparation of alkyl-functional spiro[5.5]trienone through alkylative dearomatization and the spirocyclization of biaryl ynones, respectively [29,33]. Later, Yang’s group further explored this reaction using 4-alkyl-1,4-dihydropyridines (DHPs) as radical precursors under an irradiation of visible light [32]. Although several groups have applied this radical cascade strategy to prepare alkyl-functional spiro[5.5]trienone, 5-*exo*-trig cascade reactions of biaryl ynones and alkyl radical precursors toward indenones are surprisingly rarely explored due to the chemo- and regio-selective issues of the highly reactive vinyl radical species [43]. Inspired by these works, we believe that the control of the selective cyclization sites of the electron-poor alkylated vinyl radical intermediates is interesting and still in great demand, especially those triggered by the same alkyl radical precursors. Herein, we would like to report our efforts to put this concept into practice, using 4-alkyl-DHPs as alkyl radical precursors in the presence of Na_2_S_2_O_8_ (Scheme 1d).

## 2. Results and Discussion

The study commenced with the reaction of biaryl ynone (**1a**) and 4-cyclohexyl-DHP (**2a**) under visible light irradiation conditions. In the presence of Na_2_S_2_O_8_ (2 equiv), the reaction was performed in acetone at 60 °C for 12 h (Table 1, entry 1). The five-membered indenone was obtained in a yield of 39%, which was a pleasing result. To improve the yield, we screened different solvents and found that using MeCN or AcOEt instead of acetone increased the yield (Table 1, entries 2–3). Notably, the reaction efficiency for the generation of indenone **3a** was significantly higher when using MeCN-H_2_O mixed solvents compared to single solvents (Table 1, entries 4–7). This may be due to the appropriate solubility providing necessary medium substances, which assisted the diffusion and interaction of reactants in the mixed solvents. The effect of different oxidants on this cascade reaction was also explored. When the oxidant Na_2_S_2_O_8_ was changed to K_2_S_2_O_8_, (NH_4_)_2_S_2_O_8_, TBHP or DTBP, the reaction was either inhibited or resulted in lower yields of 3a (Table 1, entries 8–11). Meanwhile, the yield did not improve significantly when the reaction temperature was increased or decreased (Table 1, entries 12 and 13). Prolonging the reaction time did not achieve better results (Table 1, entry 14). No product was detected when the reaction was performed without the oxidant Na_2_S_2_O_8_ (Table 1, entry 15).

**Scheme 2:**
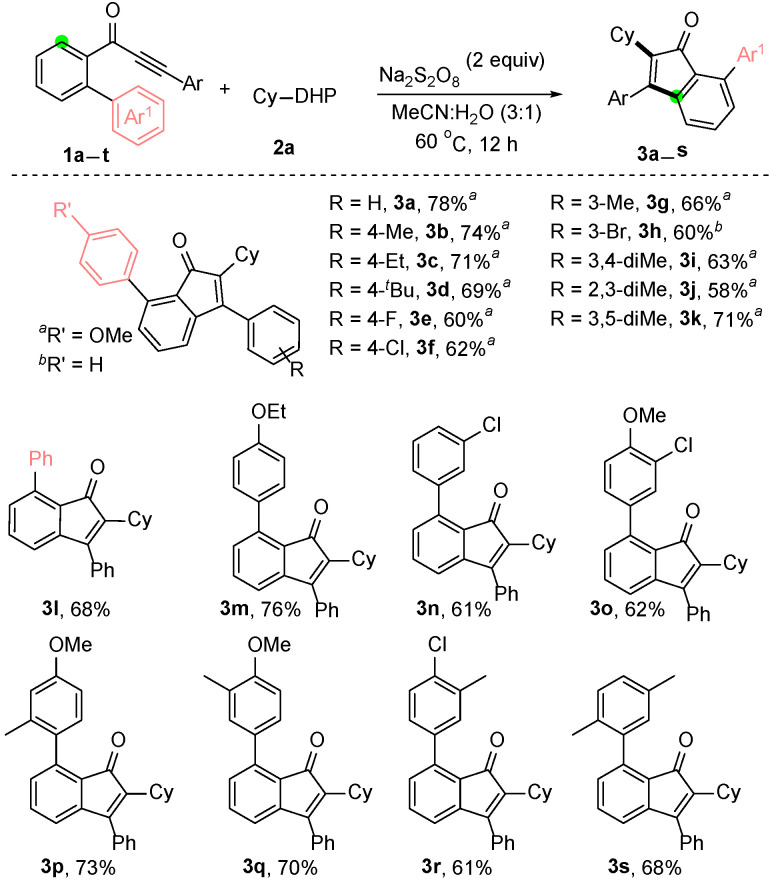
Substrate scope. All reactions were performed with **1** (0.20 mmol), R-DHP (**2**, 0.4 mmol), Na_2_S_2_O_8_ (2.0 equiv), CH_3_CN/H_2_O (3:1, 2 mL), 60 °C, 12 h. Yields are given for isolated products. *^a^* R’ = OMe; *^b^* R’ = H.

With the optimized reaction conditions, we set out to investigate the generality of this nucleophilic C-centered radical-induced cyclization of biaryl ynones with 4-cyclohexyl-DHP for the construction of 5-membered indenones (Scheme 2). To begin with, we examined the scope and limitation of biaryl ynones in this reaction. A variety of monosubstituted group on Ar ring (**1b**–**1i**) reacted well with 4-cyclohexyl-DHP to give the corresponding indenones **3b**–**3i** in yields ranging from 60% to 85%. For example, halogen substituents (−F, −Cl and −Br) on the Ar ring were well tolerated, providing opportunities for further synthetic transformations of products. Moreover, disubstituted phenyl ring **1j**–**1l** also successfully participated in the reaction, affording the target products (**3j**–**3l**) in 63–71% yields. Next, the reaction scope with different substituents on the Ar^1^ ring was investigated. The Ar^1^ ring without any substitutions was also compatible in the annulation system. Importantly, no matter whether the Ar^1^ ring in biaryl ynones was modified with either one or two groups, all of them could undergo the cascade alkylation reaction, generating the desired products (**3m**–**3s**) in yields ranging from 61% to 76%.

**Scheme 3:**
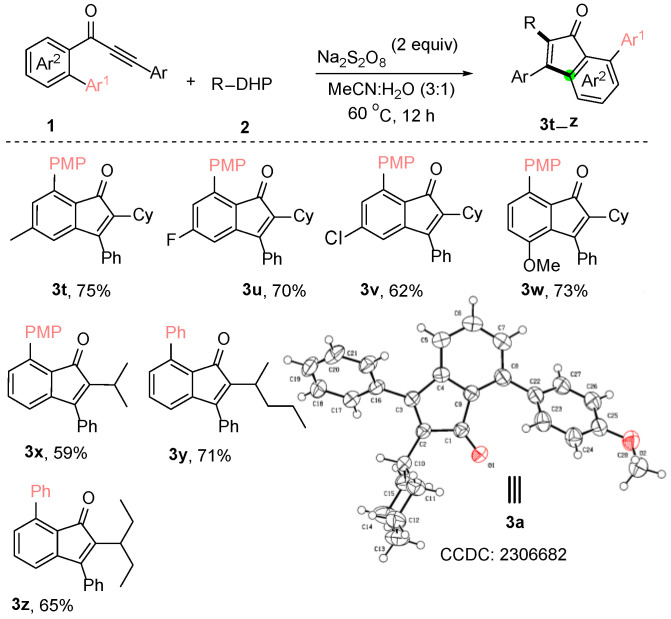
Substrate scope. All reactions were performed with **1** (0.20 mmol), R-DHP (**2**, 0.4 mmol), Na_2_S_2_O_8_ (2.0 equiv), CH_3_CN/H_2_O (3:1, 2 mL), 60 °C, 12 h. Yields are given for isolated products.

Next, we moved to assess the generality of the cyclization with different groups on the Ar^2^ ring in biaryl ynones as well as various nucleophilic C-centered radical precursors. As shown in Scheme 3, the reaction tolerated substitution around the Ar^2^ ring well, including methyl, halogens, and methoxy. For example, substrate **1v** reacted with 4-cyclohexyl-DHP well to give product **3v** in 62% yield. Furthermore, we also tested another 4-alkyl-DHP in this nucleophilic C-centered radical-induced cyclization. There was good tolerance of secondary alkyl-substituted DHP. The reaction proceeded smoothly under the standard conditions when using the bulky DHP as the substrate, yielding the desired product **3z** in a lower yield. Finally, **3a** was definitely confirmed by X-ray crystallography (CCDC 2306682).

**Scheme 4:**
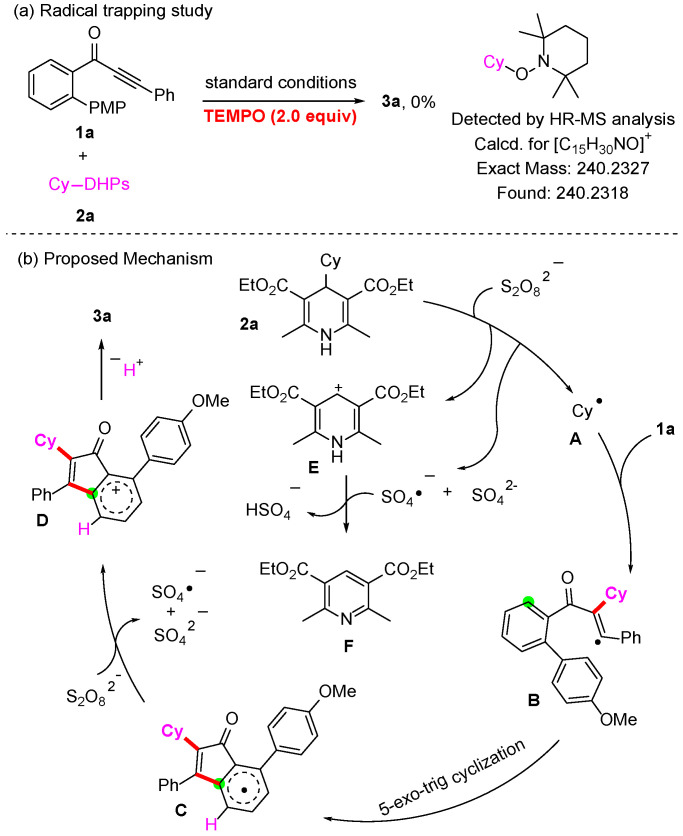
The proposed mechanism.

Preliminary experiments were conducted to gain insight into this radical cascade reaction mechanism. The alkylation reaction was almost completely inhibited when the radical-trapping reagent TEMPO was added (Scheme 4a and Supporting Information S2). During the process, the alkyl radical intermediate was captured by TEMPO, and its corresponding adduct is detected by HR-MS analysis. Based on recent studies on S_2_O_8_^2−^ salt-promoted cascade alkylation [44,45,46] and the above experimental results, we proposed a possible mechanism for this alkyl-centered radical-initiated cyclization transformation of alkynes (Scheme 4b). Initially, S_2_O_8_^2−^ salt oxidized 4-cyclohexyl-DHP to generate cyclohexyl radical **A** and cation **E**, which reacted with sulfate anions to give a pyridine derivative. For the 5-*exo*-trig cyclization, the cyclohexyl radical **A** was trapped by C–C triple bonds to afford vinyl radical **B**, which subsequently underwent 5-*exo*-trig cyclization to obtain intermediate **C**. The above-mentioned intermediate **C** was oxidized by S_2_O_8_^2−^ to provide carbocation **D**, which finally lost H^+^ to produce indenones **3a**.

In summary, we report the synthesis of a series of five-membered indenones via the alkyl-centered radical-induced cyclization of biaryl ynones, using cheap Na_2_S_2_O_8_ as an oxidant. The unfavorable enthalpic and entropic factors and variable chemo-selectivity are the challenges of this cascade reaction. Key features of this approach include straightforward and simple operational procedures, good functional group tolerance, excellent chem- and regioselectivity, and wide substrate scope.

## 3. Materials and Methods

### 3.1. General Information

All reactions were carried out under air atmosphere. ^1^H NMR and ^13^C NMR spectra were measured on a Bruker Avance NMR spectrometer (600 MHz/151 MHz/565 NMR) in CDCl_3_ as solvent and recorded in ppm relative to internal tetramethylsilane standard. ^1^H NMR data are reported as follows: δ, chemical shift; coupling constants (*J* are given in Hertz, Hz) and integration. Abbreviations to denote the multiplicity of a particular signal were s (singlet), d (doublet), t (triplet), q (quartet), dd (doublet of doublets) and m (multiplet).

### 3.2. Preparation of the Starting Materials

Biaryl ynone (**1a**) derivatives were prepared according to the reported method [29,30,31,32]. The solvents and oxidants including acetone, MeCN, K_2_S_2_O_8_, DTBP, etc. were purchased from commercial companies such as Energy Chemical (Shanghai), Shanghai Xianding Biotechnology Co., Ltd., etc. (Shanghai, China); petroleum ether and ethyl acetate were purchased from Shanghai Titan Technology Co., Ltd. (Shanghai, China). Products were purified by flash chromatography on 200–300 mesh silica gel.

### 3.3. General Procedure for the Synthesis of ***3a***

A 15 mL pressure tube was charged with biaryl ynone (**1a,** 0.2 mmol), 4-alkyl Hantzsch ester (**2a**, 0.4 mmol), Na_2_S_2_O_8_ (2 equiv, 0.4 mmol) in CH_3_CN/H_2_O (2 mL, *v*/*v* = 3:1), and a magnetic stir bar. The reaction mixture was stirred at 60 °C for 12 h (TLC tracking detection). After the reaction was finished, the mixture was diluted with brine and extracted with EtOAc. The combined organic layers were dried over anhydrous Na_2_SO_4_ and concentrated to yield the crude product, which was further purified by flash chromatography (silica gel, petroleum ether/ethyl acetate) to give the desired product **3a**.

### 3.4. Characterization Data of Products

The chemical structural formulae and ^1^H NMR and ^13^C NMR spectra of the products **3a**–**3z** can be seen in the Supporting Information S3 and S17. 

2-Cyclohexyl-7-(4-methoxyphenyl)-3-phenyl-1*H*-inden-1-one (**3a**). The product was purified by flash column chromatography on silica gel (petroleum ether/EtOAc = 20:1) to afford **3a** as a yellow solid (61 mg, 78% yield), *m.p.*: 90.5–91.8 °C. ^1^H NMR (600 MHz, CDCl_3_) δ 7.54–7.47 (m, 4H), 7.47–7.43 (m, 1H), 7.40–7.35 (m, 2H), 7.25 (t, *J* = 3.8 Hz, 1H), 7.09 (dd, *J* = 7.9, 0.6 Hz, 1H), 7.01–6.95 (m, 2H), 6.81 (dd, *J* = 7.2, 0.6 Hz, 1H), 3.86 (s, 3H), 2.44 (tt, *J* = 12.1, 3.4 Hz, 1H), 1.81 (qd, *J* = 12.4, 2.9 Hz, 2H), 1.69 (d, *J* = 12.5 Hz, 2H), 1.59 (d, *J* = 10.1 Hz, 1H), 1.54 (s, 2H), 1.22–1.09 (m, 3H). ^13^C NMR (151 MHz, CDCl_3_) δ 197.4, 159.6, 153.5, 147.0, 139.9, 139.1, 133.2, 132.6, 131.0, 130.4, 129.7, 128.7, 128.7, 128.0, 125.7, 119.2, 113.3, 55.2, 36.0, 31.0, 26.5, 25.7. HRMS (ESI) calcd for C_28_H_26_NaO_2_ [M + Na]^+^ 417.1830, found 417.1818.

2-Cyclohexyl-7-(4-methoxyphenyl)-3-(*p*-tolyl)-1*H*-inden-1-one (**3b**). The product purified by flash column chromatography on silica gel (petroleum ether/EtOAc = 20:1) to afford **3b** as a yellow solid (60 mg, 74% yield), *m.p.*: 112.6–114.1 °C. ^1^H NMR (600 MHz, CDCl_3_) δ 7.49 (d, *J* = 8.7 Hz, 2H), 7.32 (d, *J* = 7.9 Hz, 2H), 7.28 (d, *J* = 8.0 Hz, 2H), 7.24 (d, *J* = 7.4 Hz, 1H), 7.08 (d, *J* = 7.9 Hz, 1H), 6.98 (d, *J* = 8.7 Hz, 2H), 6.84 (d, *J* = 7.2 Hz, 1H), 3.88–3.85 (m, 3H), 2.45 (s, 3H), 1.83 (dd, 2H), 1.69 (d, *J* = 12.7 Hz, 2H), 1.59 (d, *J* = 10.6 Hz, 1H), 1.53 (s, 1H), 1.26 (d, *J* = 9.2 Hz, 2H), 1.16 (dd, *J* = 22.1, 11.0 Hz, 3H). ^13^C NMR (151 MHz, CDCl_3_) δ 197.4, 159.6, 153.7, 147.0, 139.8, 138.9, 138.7, 132.5, 131.0, 130.4, 130.2, 129.7, 129.3, 128.0, 125.8, 119.2, 113.3, 55.2, 36.0, 31.0, 26.6, 25.7, 21.4. HRMS (ESI) calcd for C_29_H_28_NaO_2_ [M + Na]^+^ 431.1987, found 431.1974.

2-Cyclohexyl-3-(4-ethylphenyl)-7-(4-methoxyphenyl)-1*H*-inden-1-one (**3c**). The product purified by flash column chromatography on silica gel (petroleum ether/EtOAc = 20:1) to afford **3c** as a yellow solid (60 mg, 71% yield), *m.p.*: 97–98 °C. ^1^H NMR (600 MHz, CDCl_3_) δ 7.41 (d, *J* = 8.6 Hz, 2H), 7.27 (d, *J* = 8.0 Hz, 2H), 7.24 (d, *J* = 8.0 Hz, 2H), 7.18 (d, 1H), 7.01 (d, *J* = 7.8 Hz, 1H), 6.91 (d, *J* = 8.6 Hz, 2H), 6.78 (d, *J* = 7.2 Hz, 1H), 3.79 (s, 3H), 2.68 (q, *J* = 7.6 Hz, 2H), 2.39 (ddd, *J* = 12.1, 8.9, 3.4 Hz, 1H), 1.77 (dd, *J* = 23.5, 11.0 Hz, 2H), 1.63 (d, *J* = 12.4 Hz, 2H), 1.52 (d, *J* = 10.9 Hz, 1H), 1.46 (s, 1H), 1.25 (t, *J* = 7.6 Hz, 3H), 1.16–1.06 (m, 4H). ^13^C NMR (151 MHz, CDCl_3_) δ 197.5, 159.6, 153.7, 147.0, 145.0, 139.8, 138.9, 132.5, 131.0, 130.4, 129.7, 128.1, 128.0, 125.9, 121.8, 119.3, 113.3, 55.2, 36.0, 30.9, 28.8, 26.6, 25.7, 15.3. HRMS (ESI) calcd for C_30_H_30_NaO_2_ [M + Na]^+^ 445.2143, found 445.2135.

3-(4-(*tert*-Butyl)phenyl)-2-cyclohexyl-7-(4-methoxyphenyl)-1*H*-inden-1-one (**3d**). The product purified by flash column chromatography on silica gel (petroleum ether/EtOAc = 20:1) to afford **3d** as a yellow solid (62 mg, 69% yield), *m.p.*: 107.1–108.3 °C. ^1^H NMR (600 MHz, CDCl_3_) δ 7.45 (d, *J* = 8.3 Hz, 2H), 7.41 (d, *J* = 8.7 Hz, 2H), 7.26 (d, *J* = 8.3 Hz, 2H), 7.18 (t, *J* = 3.8 Hz, 1H), 7.01 (d, *J* = 7.4 Hz, 1H), 6.91 (d, *J* = 8.7 Hz, 2H), 6.81 (d, *J* = 6.7 Hz, 1H), 3.79 (s, 3H), 2.44–2.37 (m, 1H), 1.80 (dd, 2H), 1.63 (d, *J* = 12.4 Hz, 2H), 1.52 (d, *J* = 9.8 Hz, 1H), 1.46 (s, 1H), 1.33 (s, 9H), 1.23–1.18 (m, 2H), 1.12–1.08 (m, 2H). ^13^C NMR (151 MHz, CDCl_3_) δ 196.4, 158.5, 152.5, 150.8, 145.9, 138.7, 137.8, 131.4, 129.9, 129.4, 129.0, 128.7, 126.8, 124.9, 124.5, 118.4, 112.3, 54.2, 35.0, 33.8, 30.3, 29.9, 25.5, 24.7. HRMS (ESI) calcd for C_32_H_34_NaO_2_ [M + Na]^+^ 473.2457, found 473.2442.

2-Cyclohexyl-3-(4-fluorophenyl)-7-(4-methoxyphenyl)-1*H*-inden-1-one (**3e**). The product purified by flash column chromatography on silica gel (petroleum ether/EtOAc = 20:1) to afford **3e** as a yellow solid (49 mg, 60% yield), *m.p.*: 101.3–102.1 °C. ^1^H NMR (600 MHz, CDCl_3_) δ 7.43–7.40 (m, 2H), 7.30–7.28 (m, 2H), 7.20–7.19 (m, 1H), 7.14 (t, *J* = 8.6 Hz, 2H), 7.03 (d, *J* = 7.5 Hz, 1H), 6.93–6.90 (m, 2H), 6.71 (d, *J* = 6.8 Hz, 1H), 3.79 (s, 3H), 2.33 (tt, *J* = 12.1, 3.4 Hz, 1H), 1.75–1.69 (m, 2H), 1.63 (d, *J* = 12.4 Hz, 2H), 1.53 (d, *J* = 10.3 Hz, 1H), 1.46 (d, *J* = 13.3 Hz, 2H), 1.13–1.04 (m, 3H). ^13^C NMR (151 MHz, CDCl_3_) δ 197.1, 162.8 (d, *J* = 248.6 Hz), 159.7, 152.5, 146.9, 140.0, 139.4, 132.6, 131.1, 130.4, 129.9 (d, *J* = 8.4 Hz), 129.5, 129.1 (d, *J* = 3.2 Hz), 125.6, 119.0, 115.9 (d, *J* = 21.4 Hz), 113.3, 55.2, 36.0, 31.0, 26.5, 25.7.

3-(4-Chlorophenyl)-2-cyclohexyl-7-(4-methoxyphenyl)-1*H*-inden-1-one (**3f**). The product purified by flash column chromatography on silica gel (petroleum ether/EtOAc = 20:1) to afford **3f** as a yellow solid (53 mg, 62% yield), *m.p.*: 97.6–98.5 °C. ^1^H NMR (600 MHz, CDCl_3_) δ 7.52–7.47 (m, 4H), 7.32 (d, *J* = 8.4 Hz, 2H), 7.26 (d, *J* = 2.5 Hz, 1H), 7.11 (dd, *J* = 7.9, 0.7 Hz, 1H), 6.98 (d, *J* = 8.7 Hz, 2H), 6.77 (dd, *J* = 7.2, 0.6 Hz, 1H), 3.87 (s, 3H), 2.43–2.36 (m, 1H), 1.81–1.75 (m, 2H), 1.70 (d, *J* = 12.4 Hz, 2H), 1.60 (d, *J* = 10.0 Hz, 1H), 1.54 (d, 1H), 1.28–1.25 (m, 1H), 1.18–1.11 (m, 3H). ^13^C NMR (151 MHz, CDCl_3_) δ 197.0, 159.7, 152.2, 146.7, 140.1, 139.6, 134.7, 132.7, 131.6, 131.2, 130.4, 129.5, 129.0, 125.5, 119.0, 113.3, 55.2, 36.1, 31.0, 26.5, 25.7. HRMS (ESI) calcd for C_28_H_25_ClNaO_2_ [M + Na]^+^ 451.1441, found 451.1429.

2-Cyclohexyl-7-(4-methoxyphenyl)-3-(*m*-tolyl)-1*H*-inden-1-one (**3g**). The product purified by flash column chromatography on silica gel (petroleum ether/EtOAc = 30:1) to afford **3g** as a yellow solid (54 mg, 66% yield), *m.p.*: 121.8–122.8 °C. ^1^H NMR (600 MHz, CDCl_3_) δ 7.42 (d, *J* = 8.7 Hz, 2H), 7.33 (t, *J* = 7.6 Hz, 1H), 7.19 (dd, *J* = 8.1, 5.1 Hz, 2H), 7.13–7.09 (m, 2H), 7.02 (d, *J* = 7.8 Hz, 1H), 6.92 (t, *J* = 5.8 Hz, 2H), 6.75 (d, *J* = 7.2 Hz, 1H), 3.79 (s, 3H), 2.40–2.34 (m, 4H), 1.78–1.71 (m, 2H), 1.62 (d, *J* = 12.4 Hz, 2H), 1.50 (s, 3H), 1.14–1.04 (m, 3H). ^13^C NMR (151 MHz, CDCl_3_) δ 197.4, 159.6, 153.7, 147.1, 139.8, 139.0, 138.3, 133.1, 132.5, 131.0, 130.4, 129.7, 129.5, 128.5, 128.5, 125.8, 125.1, 119.3, 113.3, 55.2, 36.0, 31.0, 26.5, 25.7, 21.6. HRMS (ESI) calcd for C_29_H_28_NaO_2_ [M + Na]^+^ 431.1987, found 431.1977.

3-(3-Bromophenyl)-2-cyclohexyl-7-phenyl-1*H*-inden-1-one (**3h**). The product purified by flash column chromatography on silica gel (petroleum ether/EtOAc = 30:1) to afford **3h** as a yellow oil (53 mg, 60% yield). ^1^H NMR (600 MHz, CDCl_3_) δ 7.64–7.57 (m, 1H), 7.55–7.50 (m, 3H), 7.47–7.43 (m, 2H), 7.43–7.38 (m, 2H), 7.31–7.28 (m, 2H), 7.12 (dd, *J* = 7.8, 0.6 Hz, 1H), 6.82 (dd, *J* = 7.2, 0.6 Hz, 1H), 2.39 (tt, *J* = 12.1, 3.4 Hz, 1H), 1.81–1.73 (m, 2H), 1.70 (dd, *J* = 9.6, 2.3 Hz, 2H), 1.60 (d, *J* = 7.7 Hz, 1H), 1.54 (d, *J* = 13.6 Hz, 2H), 1.17 (q, *J* = 12.2 Hz, 3H). ^13^C NMR (151 MHz, CDCl_3_) δ 196.8, 151.9, 146.5, 140.4, 139.8, 137.3, 135.3, 132.8, 131.8, 131.2, 130.8, 130.3, 129.0, 128.2, 127.9, 126.7, 125.7, 122.8, 119.4, 36.1, 31.0, 26.5, 25.6. HRMS (ESI) calcd for C_27_H_23_BrNaO [M + Na]^+^ 465.0830, found 465.0819.

2-Cyclohexyl-3-(3,4-dimethylphenyl)-7-(4-methoxyphenyl)-1*H*-inden-1-one (**3i**). The product purified by flash column chromatography on silica gel (petroleum ether/EtOAc = 30:1) to afford **3i** as a yellow solid (53 mg, 63% yield), *m.p.*: 107.6–108.6 °C. ^1^H NMR (600 MHz, CDCl_3_) δ 7.44 (d, *J* = 8.7 Hz, 2H), 7.17–7.10 (m, 3H), 7.00 (dd, *J* = 7.9, 0.6 Hz, 1H), 6.92 (d, *J* = 8.7 Hz, 3H), 6.41–6.36 (m, 1H), 3.79 (s, 3H), 2.30 (s, 3H), 2.20–2.14 (m, 1H), 2.10 (s, 3H), 1.58 (d, *J* = 9.2 Hz, 4H), 1.43 (d, *J* = 12.5 Hz, 2H), 1.27–1.17 (m, 2H), 1.04 (s, 2H). ^13^C NMR (151 MHz, CDCl_3_) δ 197.5, 159.6, 155.1, 147.7, 139.6, 139.4, 137.4, 134.1, 133.4, 132.8, 130.8, 130.4, 129.9, 129.6, 125.9, 125.7, 125.4, 119.2, 113.3, 55.2, 36.0, 30.9, 30.6, 26.5, 26.5, 25.7, 20.4, 17.1. HRMS (ESI) calcd for C_30_H_30_NaO_2_ [M + Na]^+^ 445.2143, found 445.2131.

2-Cyclohexyl-3-(2,3-dimethylphenyl)-7-(4-methoxyphenyl)-1*H*-inden-1-one (**3j**). The product purified by flash column chromatography on silica gel (petroleum ether/EtOAc = 20:1) to afford **3j** as a yellow solid (50 mg, 58% yield), *m.p.*: 111.4–112.4 °C. ^1^H NMR (600 MHz, CDCl_3_) δ 7.44 (d, *J* = 8.6 Hz, 2H), 7.17–7.11 (m, 3H), 7.01 (d, *J* = 7.9 Hz, 1H), 6.95–6.88 (m, 3H), 6.39 (d, *J* = 7.1 Hz, 1H), 3.80 (s, 3H), 2.30 (s, 3H), 2.17 (dd, *J* = 13.5, 10.1 Hz, 1H), 2.10 (s, 3H), 1.58 (d, *J* = 9.1 Hz, 3H), 1.43 (d, *J* = 12.6 Hz, 4H), 1.04 (s, 3H). ^13^C NMR (151 MHz, CDCl_3_) δ 197.5, 159.6, 155.1, 147.7, 139.6, 139.4, 137.4, 134.1, 133.4, 132.8, 130.8, 130.4, 129.9, 129.6, 125.9, 125.7, 125.4, 119.2, 113.3, 55.2, 36.0, 30.9, 30.6, 26.5, 26.5, 25.7, 20.4, 17.1. HRMS (ESI) calcd for C_30_H_30_NaO_2_ [M + Na]^+^ 445.2143, found 445.2136.

2-Cyclohexyl-3-(3,5-dimethylphenyl)-7-(4-methoxyphenyl)-1*H*-inden-1-one (**3k**). The product purified by flash column chromatography on silica gel (petroleum ether/EtOAc = 30:1) to afford **3k** as a yellow solid (60 mg, 71% yield), *m.p.*: 126.3–127.3 °C. ^1^H NMR (600 MHz, CDCl_3_) δ 7.43–7.39 (m, 2H), 7.18 (t, 1H), 7.01 (d, *J* = 7.3 Hz, 2H), 6.92–6.89 (m, 4H), 6.75 (d, *J* = 7.1 Hz, 1H), 3.79 (s, 3H), 2.39–2.36 (m, 1H), 2.33 (s, 6H), 1.79–1.72 (m, 2H), 1.62 (d, *J* = 12.5 Hz, 2H), 1.52 (d, *J* = 8.9 Hz, 1H), 1.46 (s, 2H), 1.13–1.05 (m, 3H). ^13^C NMR (151 MHz, CDCl_3_) δ 196.5, 158.5, 152.9, 146.1, 138.7, 137.8, 137.1, 132.0, 131.5, 129.9, 129.3, 128.7, 124.7, 124.6, 118.3, 112.2, 54.2, 35.0, 29.9, 25.5, 24.7, 20.4. HRMS (ESI) calcd for C_30_H_30_NaO_2_ [M + Na]^+^ 445.2143, found 445.2133.

2-Cyclohexyl-3,7-diphenyl-1*H*-inden-1-one (**3l**). The product purified by flash column chromatography on silica gel (petroleum ether/EtOAc = 20:1) to afford **3m** as a yellow solid (50 mg, 68% yield), *m.p.*: 89.2–90.1 °C. ^1^H NMR (600 MHz, CDCl_3_) δ 7.44 (dd, *J* = 11.3, 4.4 Hz, 4H), 7.39–7.35 (m, 3H), 7.33–7.30 (m, 3H), 7.19 (t, 1H), 7.02 (dd, *J* = 7.8, 0.7 Hz, 1H), 6.77 (dd, *J* = 7.2, 0.6 Hz, 1H), 2.36 (tt, *J* = 12.1, 7.8, 3.4 Hz, 1H), 1.72 (qd, *J* = 12.2, 6.0 Hz, 2H), 1.61 (d, *J* = 12.4 Hz, 2H), 1.48 (t, *J* = 12.5 Hz, 3H), 1.12–1.04 (m, 3H). ^13^C NMR (151 MHz, CDCl_3_) δ 197.2, 153.7, 147.0, 140.1, 139.2, 137.4, 133.2, 132.6, 131.0, 129.1, 128.8, 128.7, 128.1, 128.0, 127.9, 119.6, 36.0, 31.0, 26.6, 25.7.

2-Cyclohexyl-7-(4-ethoxyphenyl)-3-phenyl-1*H*-inden-1-one (**3m**). The product purified by flash column chromatography on silica gel (petroleum ether/EtOAc = 30:1) to afford **3m** as a yellow oil (62 mg, 76% yield). ^1^H NMR (600 MHz, CDCl_3_) δ 7.55–7.43 (m, 5H), 7.40–7.35 (m, 2H), 7.25 (t, *J* = 3.8 Hz, 1H), 7.09 (dd, *J* = 7.9, 0.7 Hz, 1H), 7.00–6.95 (m, 2H), 6.80 (dd, *J* = 7.2, 0.7 Hz, 1H), 4.10 (q, *J* = 7.0 Hz, 2H), 2.44 (tt, *J* = 12.1, 3.4 Hz, 1H), 1.86–1.77 (m, 2H), 1.69 (d, *J* = 12.5 Hz, 2H), 1.59 (d, *J* = 8.9 Hz, 1H), 1.53 (s, 2H), 1.44 (t, *J* = 7.0 Hz, 3H), 1.20–1.10 (m, 3H). ^13^C NMR (151 MHz, CDCl_3_) δ 197.3, 159.1, 153.5, 147.0, 140.0, 139.1, 133.2, 132.6, 131.0, 130.4, 129.6, 129.5, 128.7, 128.6, 128.0, 125.7, 119.2, 113.8, 63.4, 36.0, 31.0, 26.5, 25.7, 14.9. HRMS (ESI) calcd for C_29_H_28_NaO_2_ [M + Na]^+^ 431.1987, found 431.1971.

7-(3-Chlorophenyl)-2-cyclohexyl-3-phenyl-1*H*-inden-1-one (**3n**). The product purified by flash column chromatography on silica gel (petroleum ether/EtOAc = 30:1) to afford **3n** as a yellow oil (49 mg, 61% yield). ^1^H NMR (600 MHz, CDCl_3_) δ 7.52 (t, *J* = 7.4 Hz, 2H), 7.46 (d, *J* = 11.0 Hz, 2H), 7.42 (dd, *J* = 5.0, 2.0 Hz, 1H), 7.40–7.34 (m, 4H), 7.28 (t, *J* = 7.6 Hz, 1H), 7.06 (d, *J* = 7.8 Hz, 1H), 6.87 (d, *J* = 7.2 Hz, 1H), 2.48–2.41 (m, 1H), 1.84–1.74 (m, 2H), 1.70 (d, *J* = 12.1 Hz, 2H), 1.59 (d, *J* = 9.8 Hz, 1H), 1.54 (s, 2H), 1.21–1.10 (m, 3H). ^13^C NMR (151 MHz, CDCl_3_) δ 197.0, 153.8, 147.0, 139.3, 139.2, 138.4, 133.8, 133.0, 132.8, 130.6, 129.0, 128.9, 128.8, 128.7, 128.1, 128.0, 127.5, 126.1, 120.0, 36.0, 31.0, 26.5, 25.7. HRMS (ESI) calcd for C_27_H_23_ClNaO [M + Na]^+^ 421.1335, found 421.1324.

7-(3-Chloro-4-methoxyphenyl)-2-cyclohexyl-3-phenyl-1*H*-inden-1-one (**3o**). The product purified by flash column chromatography on silica gel (petroleum ether/EtOAc = 30:1) to afford **3o** as a yellow solid (53 mg, 62% yield), *m.p.*: 99.5–100.7 °C. ^1^H NMR (600 MHz, CDCl_3_) δ 7.54–7.50 (m, 3H), 7.48–7.45 (m, 2H), 7.40–7.37 (m, 2H), 7.26 (d, *J* = 2.5 Hz, 1H), 7.06 (d, *J* = 7.4 Hz, 1H), 7.01 (d, *J* = 8.5 Hz, 1H), 6.85–6.82 (m, 1H), 3.96 (s, 3H), 2.44 (tt, *J* = 12.1, 3.4 Hz, 1H), 1.83–1.76 (m, 2H), 1.70 (d, *J* = 12.5 Hz, 2H), 1.55 (d, *J* = 13.4 Hz, 2H), 1.23–1.12 (m, 4H). ^13^C NMR (151 MHz, CDCl_3_) δ 197.2, 154.9, 153.7, 147.1, 139.2, 138.4, 133.1, 132.7, 130.7, 130.6, 130.6, 128.9, 128.8, 128.7, 128.0, 125.9, 121.9, 119.7, 111.2, 56.1, 36.0, 31.0, 26.5, 25.7. HRMS (ESI) calcd for C_28_H_25_ClNaO_2_ [M + Na]^+^ 451.1441, found 451.1436.

2-Cyclohexyl-7-(4-methoxy-2-methylphenyl)-3-phenyl-1*H*-inden-1-one (**3p**). The product purified by flash column chromatography on silica gel (petroleum ether/EtOAc = 30:1) to afford **3p** as a yellow solid (60 mg, 73% yield), *m.p.*: 82.4–83.4 °C. ^1^H NMR (600 MHz, CDCl_3_) δ 7.52 (dd, *J* = 9.3, 5.5 Hz, 2H), 7.48–7.44 (m, 1H), 7.41–7.39 (m, 2H), 7.24 (d, *J* = 7.5 Hz, 1H), 7.11 (d, *J* = 8.3 Hz, 1H), 6.98–6.95 (m, 1H), 6.87–6.83 (m, 2H), 6.80 (dd, *J* = 8.3, 2.6 Hz, 1H), 3.84 (s, 3H), 2.42 (tt, *J* = 12.1, 3.4 Hz, 1H), 2.16 (s, 3H), 1.81–1.76 (m, 2H), 1.68 (d, *J* = 9.5 Hz, 2H), 1.53 (s, 2H), 1.25 (s, 2H), 1.14 (d, *J* = 9.9 Hz, 2H). ^13^C NMR (151 MHz, CDCl_3_) δ 197.5, 159.1, 153.7, 146.3, 139.1, 138.9, 137.2, 133.2, 132.3, 131.5, 130.3, 130.0, 128.7, 128.6, 128.0, 127.1, 119.3, 115.2, 110.8, 55.1, 35.9, 30.9, 26.5, 25.7, 20.4. HRMS (ESI) calcd for C_29_H_28_NaO_2_ [M + Na]^+^ 431.1987, found 431.1975.

2-Cyclohexyl-7-(4-methoxy-3-methylphenyl)-3-phenyl-1*H*-inden-1-one (**3q**). The product purified by flash column chromatography on silica gel (petroleum ether/EtOAc = 30:1) to afford **3q** as a yellow solid (57 mg, 70% yield), *m.p.*: 101.5–102.5 °C. ^1^H NMR (600 MHz, CDCl_3_) δ 7.52 (t, *J* = 7.5 Hz, 2H), 7.45 (t, *J* = 7.4 Hz, 1H), 7.42–7.35 (m, 3H), 7.29 (s, 1H), 7.24 (t, *J* = 7.6 Hz, 1H), 7.09 (d, *J* = 7.9 Hz, 1H), 6.91 (d, *J* = 8.4 Hz, 1H), 6.80 (d, *J* = 7.2 Hz, 1H), 3.89 (s, 3H), 2.44 (tt, *J* = 12.1, 3.5 Hz, 1H), 2.29 (d, *J* = 5.3 Hz, 3H), 1.84–1.76 (m, 2H), 1.69 (d, *J* = 12.1 Hz, 2H), 1.60 (s, 1H), 1.54 (d, *J* = 13.4 Hz, 2H), 1.20–1.12 (m, 3H). ^13^C NMR (151 MHz, CDCl_3_) δ 197.3, 157.8, 153.4, 147.0, 140.2, 139.1, 133.3, 132.5, 131.2, 131.1, 129.2, 128.7, 128.6, 128.1, 128.0, 126.0, 125.7, 119.1, 109.1, 55.3, 36.0, 31.0, 26.6, 25.7, 16.3. HRMS (ESI) calcd for C_29_H_28_NaO_2_ [M + Na]^+^ 431.1987, found 431.1975.

7-(4-Chloro-3-methylphenyl)-2-cyclohexyl-3-phenyl-1*H*-inden-1-one (**3r**). The product purified by flash column chromatography on silica gel (petroleum ether/EtOAc = 30:1) to afford **3r** as a yellow oil (50 mg, 61% yield). ^1^H NMR (600 MHz, CDCl_3_) δ 7.51 (t, *J* = 7.4 Hz, 2H), 7.46 (d, *J* = 7.3 Hz, 1H), 7.39–7.33 (m, 2H), 7.25–7.23 (m, 2H), 7.11–7.04 (m, 3H), 6.81 (d, *J* = 7.2 Hz, 1H), 2.47–2.41 (m, 1H), 2.38 (s, 3H), 1.78 (dt, *J* = 22.0, 7.7 Hz, 2H), 1.70–1.68 (m, 2H), 1.59–1.53 (m, 3H), 1.20–1.13 (m, 3H). ^13^C NMR (151 MHz, CDCl_3_) δ 197.0, 153.5, 146.9, 140.4, 139.1, 137.5, 137.2, 133.3, 132.4, 131.1, 129.7, 128.7, 128.6, 128.0, 126.8, 126.0, 119.4, 35.9, 31.0, 26.6, 25.7, 21.4.

2-Cyclohexyl-7-(2,5-dimethylphenyl)-3-phenyl-1*H*-inden-1-one (**3s**). The product purified by flash column chromatography on silica gel (petroleum ether/EtOAc = 30:1) to afford **3s** as a yellow oil (53 mg, 68% yield). ^1^H NMR (600 MHz, CDCl_3_) δ 7.52 (t, *J* = 7.4 Hz, 2H), 7.48–7.44 (m, 1H), 7.42–7.38 (m, 2H), 7.26 (t, *J* = 7.5 Hz, 1H), 7.16 (d, *J* = 7.7 Hz, 1H), 7.11 (dd, *J* = 7.7, 1.2 Hz, 1H), 6.98–6.94 (m, 2H), 6.86 (dd, *J* = 7.3, 0.7 Hz, 1H), 2.42 (tt, *J* = 12.1, 3.4 Hz, 1H), 2.34 (s, 3H), 2.11 (s, 3H), 1.82–1.74 (m, 2H), 1.68 (s, 2H), 1.56 (s, 1H), 1.51 (d, *J* = 11.5 Hz, 2H), 1.18–1.09 (m, 3H). ^13^C NMR (151 MHz, CDCl_3_) δ 197.3, 153.7, 146.2, 139.2, 139.0, 137.8, 134.7, 133.2, 132.6, 132.4, 131.0, 129.5, 129.3, 128.7, 128.6, 128.6, 128.0, 127.0, 119.4, 35.9, 30.9, 30.9, 26.5, 25.7, 21.0, 19.5. HRMS (ESI) calcd for C_29_H_28_NaO [M + Na]^+^ 415.2038, found 415.2025.

2-Cyclohexyl-7-(4-methoxyphenyl)-5-methyl-3-phenyl-1*H*-inden-1-one (**3t**). The product purified by flash column chromatography on silica gel (petroleum ether/EtOAc = 30:1) to afford **3t** as a yellow solid (61 mg, 75% yield), *m.p.*: 118.3–119.7 °C. ^1^H NMR (600 MHz, CDCl_3_) δ 7.52 (dd, *J* = 10.2, 4.6 Hz, 2H), 7.50–7.45 (m, 3H), 7.39–7.36 (m, 2H), 6.98 (dd, 2H), 6.89 (s, 1H), 6.61 (s, 1H), 3.86 (s, 3H), 2.42 (tt, *J* = 12.1, 3.3 Hz, 1H), 2.29 (s, 3H), 1.83–1.76 (m, 2H), 1.69 (d, *J* = 12.5 Hz, 2H), 1.56–1.52 (m, 2H), 1.24–1.07 (m, 4H). ^13^C NMR (151 MHz, CDCl_3_) δ 197.0, 159.6, 153.1, 147.6, 143.4, 139.9, 139.6, 133.4, 130.9, 130.3, 129.8, 128.6, 128.6, 128.1, 123.4, 120.6, 113.3, 55.2, 36.0, 31.0, 26.6, 25.7, 21.8. HRMS (ESI) calcd for C_29_H_28_NaO_2_ [M + Na]^+^ 431.1987, found 431.1976.

2-Cyclohexyl-5-fluoro-7-(4-methoxyphenyl)-3-phenyl-1*H*-inden-1-one (**3u**). The product purified by flash column chromatography on silica gel (petroleum ether/EtOAc = 30:1) to afford **3u** as a yellow solid (58 mg, 70% yield), *m.p.*: 119.0–120.1 °C. ^1^H NMR (600 MHz, CDCl_3_) δ 7.53 (t, *J* = 7.4 Hz, 2H), 7.50–7.44 (m, 3H), 7.36 (dd, *J* = 5.1, 3.2 Hz, 2H), 7.01–6.96 (m, 2H), 6.75 (dd, *J* = 10.0, 2.2 Hz, 1H), 6.53 (dd, *J* = 8.1, 2.2 Hz, 1H), 3.86 (s, 3H), 2.45 (tt, *J* = 12.1, 3.4 Hz, 1H), 1.84–1.75 (m, 2H), 1.70 (d, *J* = 12.3 Hz, 2H), 1.59 (d, *J* = 10.1 Hz, 1H), 1.54 (s, 2H), 1.19–1.10 (m, 3H). ^13^C NMR (151 MHz, CDCl_3_) δ 195.6, 165.5 (d, *J* = 254.3 Hz), 160.0, 151.6, 150.7 (d, *J* = 9.4 Hz), 142.3, 142.2, 140.7, 132.6, 130.3, 128.9, 128.8, 128.6, 128.0, 121.7, 115.7 (d, *J* = 22.5 Hz), 113.4, 108.1 (d, *J* = 25.3 Hz), 55.3, 36.1, 31.0, 26.5, 25.7. HRMS (ESI) calcd for C_28_H_25_FNaO_2_ [M + Na]^+^ 435.1736, found 435.1736.

5-Chloro-2-cyclohexyl-7-(4-methoxyphenyl)-3-phenyl-1*H*-inden-1-one (**3v**). The product purified by flash column chromatography on silica gel (petroleum ether/EtOAc = 30:1) to afford **3v** as a yellow solid (53 mg, 62% yield), *m.p.*: 93.5–94.3 °C. ^1^H NMR (600 MHz, CDCl_3_) δ 7.54 (dd, *J* = 10.2, 4.6 Hz, 2H), 7.49–7.46 (m, 3H), 7.36 (dd, *J* = 5.2, 3.2 Hz, 2H), 7.10 (d, *J* = 1.7 Hz, 1H), 7.00–6.96 (m, 2H), 6.77 (d, *J* = 1.7 Hz, 1H), 3.87 (s, 3H), 2.44 (tt, *J* = 12.1, 3.4 Hz, 1H), 1.83–1.75 (m, 2H), 1.70 (d, *J* = 12.2 Hz, 2H), 1.54 (d, *J* = 13.4 Hz, 2H), 1.23–1.09 (m, 4H). ^13^C NMR (151 MHz, CDCl_3_) δ 195.9, 160.0, 152.5, 149.2, 141.1, 140.5, 138.6, 132.6, 130.4, 130.0, 129.0, 128.8, 128.4, 128.0, 123.9, 119.8, 113.4, 55.3, 36.1, 30.9, 26.5, 25.7. HRMS (ESI) calcd for C_28_H_25_ClNaO_2_ [M + Na]^+^ 451.1441, found 451.1426.

2-Cyclohexyl-4-methoxy-7-(4-methoxyphenyl)-3-phenyl-1*H*-inden-1-one (**3w**). The product purified by flash column chromatography on silica gel (petroleum ether/EtOAc = 50:1) to afford **3w** as a yellow oil (62 mg, 73% yield). ^1^H NMR (600 MHz, CDCl_3_) δ 7.44–7.40 (m, 4H), 7.39–7.35 (m, 1H), 7.32–7.29 (m, 2H), 7.05 (d, *J* = 8.6 Hz, 1H), 6.98–6.95 (m, 2H), 6.90 (d, *J* = 8.6 Hz, 1H), 3.86 (s, 3H), 3.49 (s, 3H), 2.25 (tt, *J* = 12.2, 3.5 Hz, 1H), 1.76–1.72 (m, 2H), 1.64 (d, *J* = 12.9 Hz, 2H), 1.53 (s, 1H), 1.49–1.45 (m, 2H), 1.15–1.03 (m, 3H). ^13^C NMR (151 MHz, CDCl_3_) δ 197.3, 159.3, 154.6, 152.3, 138.7, 135.8, 133.4, 133.0, 131.4, 130.3, 129.7, 129.4, 128.2, 127.8, 127.7, 127.5, 127.2, 119.5, 113.3, 55.9, 55.2, 35.7, 30.9, 26.5, 25.7. HRMS (ESI) calcd for C_29_H_28_NaO_3_ [M + Na]^+^ 447.1936, found 447.1960.

2-Isopropyl-7-(4-methoxyphenyl)-3-phenyl-1*H*-inden-1-one (**3x**). The product purified by flash column chromatography on silica gel (petroleum ether/EtOAc = 50:1) to afford **3x** as a yellow solid (42 mg, 59% yield), *m.p.*: 90.8–91.7 °C. ^1^H NMR (600 MHz, CDCl_3_) δ 7.51 (t, *J* = 8.2 Hz, 4H), 7.45 (t, *J* = 7.4 Hz, 1H), 7.41–7.37 (m, 2H), 7.26 (d, *J* = 8.0 Hz, 1H), 7.10 (d, *J* = 7.3 Hz, 1H), 6.99 (d, *J* = 8.7 Hz, 2H), 6.83 (d, *J* = 6.6 Hz, 1H), 3.87 (s, 3H), 2.81 (dt, *J* = 13.9, 7.0 Hz, 1H), 1.20 (d, *J* = 7.0 Hz, 6H). ^13^C NMR (151 MHz, CDCl_3_) δ 197.2, 159.7, 153.2, 147.0, 139.9, 139.8, 133.1, 132.6, 131.0, 130.4, 129.6, 128.7, 128.6, 128.0, 125.8, 119.3, 113.3, 55.2, 25.5, 21.4. HRMS (ESI) calcd for C_25_H_22_NaO_2_ [M + Na]^+^ 377.1517, found 377.1505.

2-(Pentan-2-yl)-3,7-diphenyl-1*H*-inden-1-one (**3y**). The product purified by flash column chromatography on silica gel (petroleum ether/EtOAc = 50:1) to afford **3y** as a yellow oil (50 mg, 71% yield). ^1^H NMR (600 MHz, CDCl_3_) δ 7.48–7.41 (m, 4H), 7.39–7.36 (m, 2H), 7.34–7.29 (m, 3H), 7.19 (dd, *J* = 17.2, 9.7 Hz, 2H), 7.07–7.02 (m, 1H), 6.76 (dd, *J* = 7.2, 0.5 Hz, 1H), 2.59–2.53 (m, 1H), 1.68–1.58 (m, 1H), 1.38–1.32 (m, 1H), 1.13–1.08 (m, 5H), 0.67 (t, *J* = 7.3 Hz, 3H). ^13^C NMR (151 MHz, CDCl_3_) δ 197.1, 154.3, 147.0, 140.1, 139.0, 137.4, 133.1, 132.7, 131.0, 129.1, 128.7, 128.7, 128.1, 128.0, 127.9, 126.0, 119.5, 37.2, 30.6, 21.3, 19.8, 13.9.

2-(Pentan-3-yl)-3,7-diphenyl-1*H*-inden-1-one (**3z**). The product purified by flash column chromatography on silica gel (petroleum ether/EtOAc = 50:1) to afford **3z** as a yellow oil (46 mg, 65% yield). ^1^H NMR (600 MHz, CDCl_3_) δ 7.48–7.45 (m, 2H), 7.42 (t, *J* = 7.4 Hz, 2H), 7.37 (t, *J* = 7.1 Hz, 3H), 7.33 (d, *J* = 7.3 Hz, 1H), 7.31–7.28 (m, 2H), 7.20 (t, *J* = 7.6 Hz, 1H), 7.07–7.02 (m, 1H), 6.72 (d, *J* = 7.2 Hz, 1H), 2.27–2.20 (m, 1H), 1.69–1.59 (m, 2H), 1.48–1.42 (m, 2H), 0.72 (t, *J* = 7.5 Hz, 6H). ^13^C NMR (151 MHz, CDCl_3_) 197.2, 156.3, 147.2, 140.1, 137.3, 137.2, 133.2, 132.7, 131.1, 129.1, 128.6, 128.6, 128.1, 128.0, 127.9, 125.9, 119.5, 40.2, 26.6, 12.8.

## Figures and Tables

**Table 1:** Optimization of the reaction conditions *^a^*.

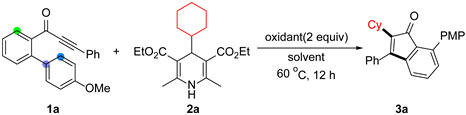			
Entry	Oxidant	Solvent	Yield (%) *^b^*
1	Na_2_S_2_O_8_	acetone	39
2	Na_2_S_2_O_8_	MeCN	67
3	Na_2_S_2_O_8_	AcOEt	56
4	Na_2_S_2_O_8_	MeCN/H_2_O (3:1)	78
5	Na_2_S_2_O_8_	MeCN/H_2_O (2:1)	70
6	Na_2_S_2_O_8_	MeCN/H_2_O (4:1)	55
7	Na_2_S_2_O_8_	AcOEt/H_2_O (3:1)	61
8	K_2_S_2_O_8_	MeCN/H_2_O (3:1)	65
9	(NH_4_)_2_S_2_O_8_	MeCN/H_2_O (3:1)	48
10	TBHP	MeCN/H_2_O (3:1)	22
11	DTBP	MeCN/H_2_O (3:1)	27
12 *^c^*	Na_2_S_2_O_8_	MeCN/H_2_O (3:1)	77
13 *^d^*	Na_2_S_2_O_8_	MeCN/H_2_O (3:1)	51
14 *^e^*	Na_2_S_2_O_8_	MeCN/H_2_O (3:1)	63
15	-	MeCN/H_2_O (3:1)	n.d.

*^a^ *Reaction conditions: (**1a**, 0.20 mmol), Cy-DHP (**2a**, 0.40 mmol), Na_2_S_2_O_8_ (2.0 equiv), CH_3_CN/H_2_O (3:1, 2 mL), 60 °C, 12 h. *^b^* Isolated yields. *^c^* At 80 °C. *^d^* At 40 °C. *^e^* For 24 h. n.d. = not detected. TBHP: *tert*-butyl hydroperoxide; DTBP: di-*tert*-butyl peroxide.

## Data Availability

Data are contained within the article and Supplementary Materials.

## Supplementary Materials

The following supporting information can be downloaded at https://www.mdpi.com/article/10.3390/molecules29020458/s1. The supporting information include general considerations, radical trapping experiment, characterization data of products, and ^1^H NMR and ^13^C NMR spectra of the products.
