# Supplementary material for: Synthesis of Indenones via Persulfate Promoted Radical Alkylation/Cyclization of Biaryl Ynones with 1,4-Dihydropyridines
Source: Molecules. 2024 Jan 17;29(2):458. doi: 10.3390/molecules29020458 (PMC10818456; doi:10.3390/molecules29020458)

Supporting Information for

Synthesis of indenones via persulfate promoted radical alkylation/cyclization of biaryl ynones with 1,4-dihydropyridines

Wanwan Wang * and Lei Yu

Jiangsu Key Laboratory of Chiral Pharmaceuticals Biosynthesis, Taizhou University, Taizhou 225300, China.

Table of Contents for Supporting Information

General considerations……………………………………………………..………..S2

Radical trapping experiment……………………….…………………………..……S2

Characterization data of products...S3

^{1}H NMR and ^{13}C NMR spectra of the products....................................S17

General considerations

All reactions were carried out under air. ^{1}H NMR and ^{13}C NMR spectra were measured on a Bruker Avance NMR spectrometer (600 MHz/151 MHz/565 NMR) in $CDCl_3$ as solvent and recorded in ppm relative to internal tetramethylsilane standard. ^{1}H NMR data are reported as follows: δ, chemical shift; coupling constants (J are given in Hertz, Hz) and integration. Abbreviations to denote the multiplicity of a particular signal were s (singlet), d (doublet), t (triplet), q (quartet), dd (doublet of doublets) and m (multiplet).

Radical trapping experiment

(a) Radical trapping study

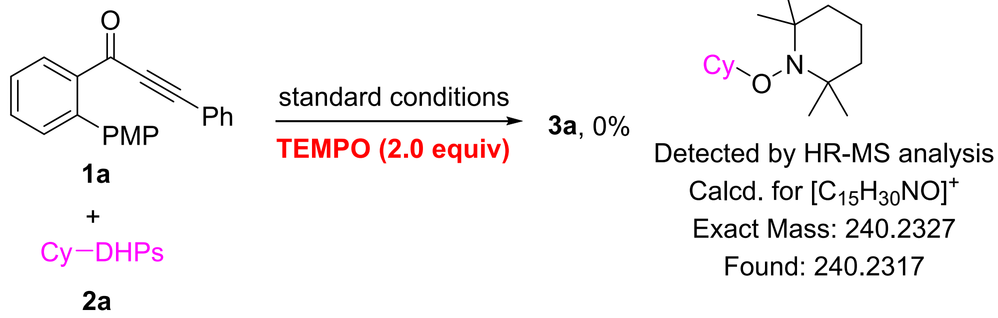

TEMPO (2.0 equiv) was subjected to the standard conditions and reacted for 12 hours. The reaction was fully inhibited and no desired product could be detected. The TEMPO-adduct could be detected by HRMS analysis of the reaction system (see below).

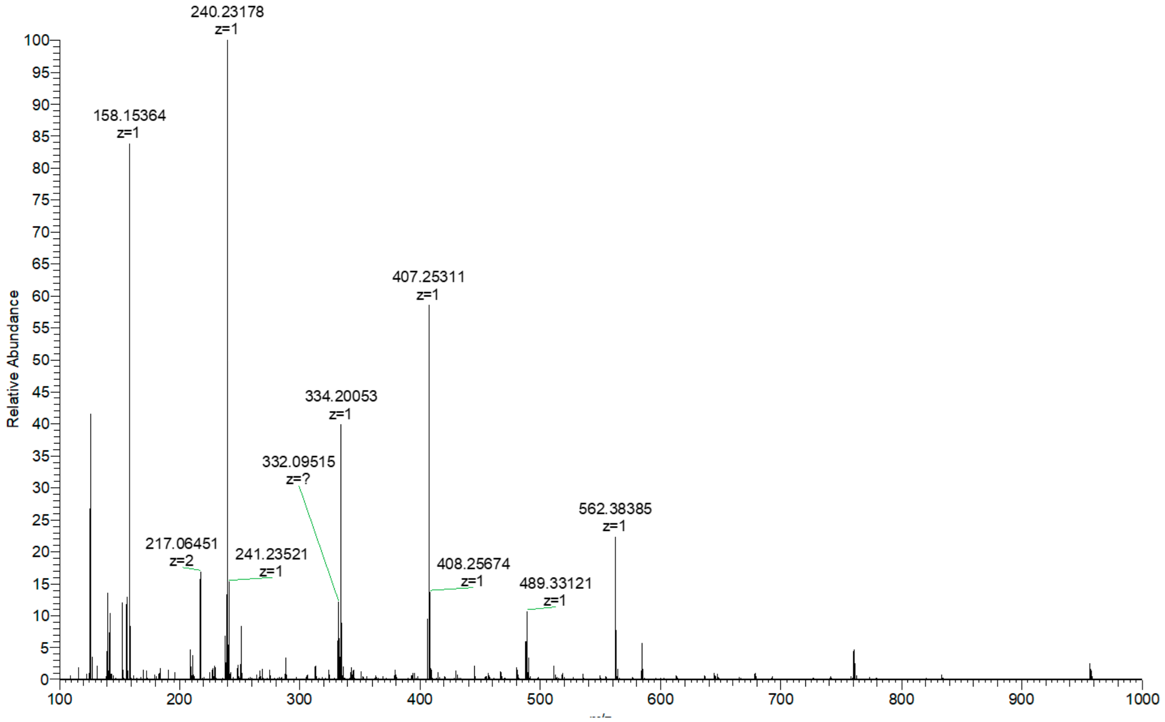

Characterization data of products

2-Cyclohexyl-7-(4-methoxyphenyl)-3-phenyl-1*H*-inden-1-one (3a)

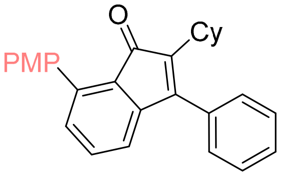

3a

The product purified by flash column chromatography on silica gel (petroleum ether/EtOAc = 20:1) to afford the **3a** as a yellow solid (61 mg, 78% yield). ^{1}H NMR (600 MHz, $CDCl_3$) δ 7.54 – 7.47 (m, 4H), 7.47 – 7.43 (m, 1H), 7.40 – 7.35 (m, 2H), 7.25 (t, *J* = 3.8 Hz, 1H), 7.09 (dd, *J* = 7.9, 0.6 Hz, 1H), 7.01 – 6.95 (m, 2H), 6.81 (dd, *J* = 7.2, 0.6 Hz, 1H), 3.86 (s, 3H), 2.44 (tt, *J* = 12.1, 3.4 Hz, 1H), 1.81 (qd, *J* = 12.4, 2.9 Hz, 2H), 1.69 (d, *J* = 12.5 Hz, 2H), 1.59 (d, *J* = 10.1 Hz, 1H), 1.54 (s, 2H), 1.22 – 1.09 (m, 3H). ^{13}C NMR (151 MHz, $CDCl_3$) δ 197.4, 159.6, 153.5, 147.0, 139.9, 139.1, 133.2, 132.6, 131.0, 130.4, 129.7, 128.7, 128.7, 128.0, 125.7, 119.2, 113.3, 55.2, 36.0, 31.0,

26.5, 25.7. HRMS (ESI) calcd for $C_{28}H_{26}NaO_2$ $[M+Na]^+$ 417.1830, found 417.1818.

2-Cyclohexyl-7-(4-methoxyphenyl)-3-(*p*-tolyl)-1*H*-inden-1-one (3b)

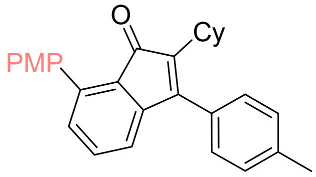

3b

The product purified by flash column chromatography on silica gel (petroleum ether/EtOAc = 20:1) to afford the **3b** as a yellow solid (60 mg, 74% yield). ^1H NMR (600 MHz, $CDCl_3$) δ 7.49 (d, *J* = 8.7 Hz, 2H), 7.32 (d, *J* = 7.9 Hz, 2H), 7.28 (d, *J* = 8.0 Hz, 2H), 7.24 (d, *J* = 7.4 Hz, 1H), 7.08 (d, *J* = 7.9 Hz, 1H), 6.98 (d, *J* = 8.7 Hz, 2H), 6.84 (d, *J* = 7.2 Hz, 1H), 3.88 – 3.85 (m, 3H), 2.45 (s, 3H), 1.83 (dd, 2H), 1.69 (d, *J* = 12.7 Hz, 2H), 1.59 (d, *J* = 10.6 Hz, 1H), 1.53 (s, 1H), 1.26 (d, *J* = 9.2 Hz, 2H), 1.16 (dd, *J* = 22.1, 11.0 Hz, 3H). ^{13}C NMR (151 MHz, $CDCl_3$) δ 197.4, 159.6, 153.7, 147.0, 139.8, 138.9, 138.7, 132.5, 131.0, 130.4, 130.2, 129.7, 129.3, 128.0, 125.8, 119.2, 113.3, 55.2, 36.0, 31.0, 26.6, 25.7, 21.4. HRMS (ESI) calcd for $C_{29}H_{28}NaO_2$ $[M+Na]^+$ 431.1987, found 431.1974.

2-Cyclohexyl-3-(4-ethylphenyl)-7-(4-methoxyphenyl)-1*H*-inden-1-one (3c)

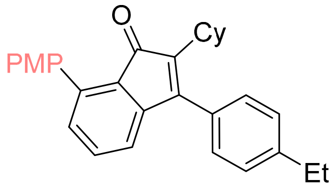

3c

The product purified by flash column chromatography on silica gel (petroleum ether/EtOAc = 20:1) to afford the **3c** as a yellow solid (60 mg, 71% yield). ^1H NMR (600 MHz, $CDCl_3$) δ 7.41 (d, *J* = 8.6 Hz, 2H), 7.27 (d, *J* = 8.0 Hz, 2H), 7.24 (d, *J* = 8.0 Hz, 2H), 7.18 (d, 1H), 7.01 (d, *J* = 7.8 Hz, 1H), 6.91 (d, *J* = 8.6 Hz, 2H), 6.78 (d, *J* = 7.2 Hz, 1H), 3.79 (s, 3H), 2.68 (q, *J* = 7.6 Hz, 2H), 2.39 (ddd, *J* = 12.1, 8.9, 3.4 Hz, 1H), 1.77 (dd, *J* = 23.5, 11.0 Hz, 2H), 1.63 (d, *J* = 12.4 Hz, 2H), 1.52 (d, *J* = 10.9 Hz, 1H), 1.46 (s, 1H), 1.25 (t, *J* =

7.6 Hz, 3H), 1.16 – 1.06 (m, 4H). ^{13}C NMR (151 MHz, $CDCl_3$) δ 197.5, 159.6, 153.7, 147.0, 145.0, 139.8, 138.9, 132.5, 131.0, 130.4, 129.7, 128.1, 128.0, 125.9, 121.8, 119.3, 113.3, 55.2, 36.0, 30.9, 28.8, 26.6, 25.7, 15.3. HRMS (ESI) calcd for $C_{30}H_{30}NaO_2$ $[M+Na]^+$ 445.2143, found 445.2135.

3-(4-(*tert*-Butyl)phenyl)-2-cyclohexyl-7-(4-methoxyphenyl)-1*H*-inden-1-one (3d)

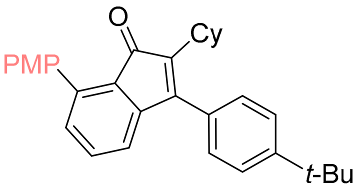

3d

The product purified by flash column chromatography on silica gel (petroleum ether/EtOAc = 20:1) to afford the **3d** as a yellow solid (62 mg, 69% yield). ^{1}H NMR (600 MHz, $CDCl_3$) δ 7.45 (d, *J* = 8.3 Hz, 2H), 7.41 (d, *J* = 8.7 Hz, 2H), 7.26 (d, *J* = 8.3 Hz, 2H), 7.18 (t, *J* = 3.8 Hz, 1H), 7.01 (d, *J* = 7.4 Hz, 1H), 6.91 (d, *J* = 8.7 Hz, 2H), 6.81 (d, *J* = 6.7 Hz, 1H), 3.79 (s, 3H), 2.44 – 2.37 (m, 1H), 1.80 (dd, 2H), 1.63 (d, *J* = 12.4 Hz, 2H), 1.52 (d, *J* = 9.8 Hz, 1H), 1.46 (s, 1H), 1.33 (s, 9H), 1.23 – 1.18 (m, 2H), 1.12 – 1.08 (m, 2H). ^{13}C NMR (151 MHz, $CDCl_3$) δ 196.4, 158.5, 152.5, 150.8, 145.9, 138.7, 137.8, 131.4, 129.9, 129.4, 129.0, 128.7, 126.8, 124.9, 124.5, 118.4, 112.3, 54.2, 35.0, 33.8, 30.3, 29.9, 25.5, 24.7. HRMS (ESI) calcd for $C_{32}H_{34}NaO_2$ $[M+Na]^+$ 473.2457, found 473.2442.

2-Cyclohexyl-3-(4-fluorophenyl)-7-(4-methoxyphenyl)-1*H*-inden-1-one (3e)

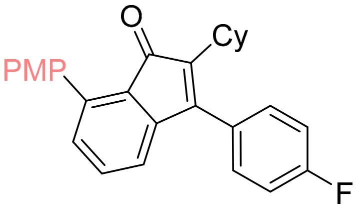

3e

The product purified by flash column chromatography on silica gel (petroleum ether/EtOAc = 20:1) to afford the **3e** as a yellow solid (49 mg, 60% yield). ^{1}H NMR (600 MHz, $CDCl_3$) δ 7.43 – 7.40 (m, 2H), 7.30 – 7.28

(m, 2H), 7.20 – 7.19 (m, 1H), 7.14 (t, *J* = 8.6 Hz, 2H), 7.03 (d, *J* = 7.5 Hz, 1H), 6.93 – 6.90 (m, 2H), 6.71 (d, *J* = 6.8 Hz, 1H), 3.79 (s, 3H), 2.33 (tt, *J* = 12.1, 3.4 Hz, 1H), 1.75 – 1.69 (m, 2H), 1.63 (d, *J* = 12.4 Hz, 2H), 1.53 (d, *J* = 10.3 Hz, 1H), 1.46 (d, *J* = 13.3 Hz, 2H), 1.13 – 1.04 (m, 3H). ^{13}C NMR (151 MHz, $CDCl_3$) δ 197.1, 162.8 (d, *J* = 248.6 Hz), 159.7, 152.5, 146.9, 140.0, 139.4, 132.6, 131.1, 130.4, 129.9 (d, *J* = 8.4 Hz), 129.5, 129.1 (d, *J* = 3.2 Hz), 125.6, 119.0, 115.9 (d, *J* = 21.4 Hz), 113.3, 55.2, 36.0, 31.0, 26.5, 25.7.

3-(4-Chlorophenyl)-2-cyclohexyl-7-(4-methoxyphenyl)-1*H*-inden-1-one (3f)

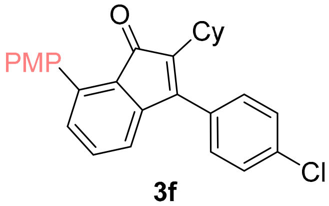

3f

The product purified by flash column chromatography on silica gel (petroleum ether/EtOAc = 20:1) to afford the **3f** as a yellow solid (53 mg, 62% yield). ^{1}H NMR (600 MHz, $CDCl_3$) δ 7.52 – 7.47 (m, 4H), 7.32 (d, *J* = 8.4 Hz, 2H), 7.26 (d, *J* = 2.5 Hz, 1H), 7.11 (dd, *J* = 7.9, 0.7 Hz, 1H), 6.98 (d, *J* = 8.7 Hz, 2H), 6.77 (dd, *J* = 7.2, 0.6 Hz, 1H), 3.87 (s, 3H), 2.43 – 2.36 (m, 1H), 1.81 – 1.75 (m, 2H), 1.70 (d, *J* = 12.4 Hz, 2H), 1.60 (d, *J* = 10.0 Hz, 1H), 1.54 (d, 1H), 1.28 – 1.25 (m, 1H), 1.18 – 1.11 (m, 3H). ^{13}C NMR (151 MHz, $CDCl_3$) δ 197.0, 159.7, 152.2, 146.7, 140.1, 139.6, 134.7, 132.7, 131.6, 131.2, 130.4, 129.5, 129.0, 125.5, 119.0, 113.3, 55.2, 36.1, 31.0, 26.5, 25.7. HRMS (ESI) calcd for $C_{28}H_{25}ClNaO_2$ $[M+Na]^+$ 451.1441, found 451.1429.

2-Cyclohexyl-7-(4-methoxyphenyl)-3-(*m*-tolyl)-1*H*-inden-1-one (3g)

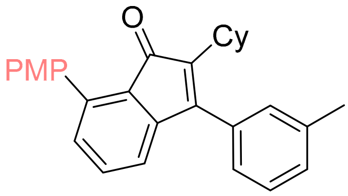

3g

The product purified by flash column chromatography on silica gel (petroleum ether/EtOAc = 30:1) to afford the **3g** as a yellow

solid (54 mg, 66% yield). ^{1}H NMR (600 MHz, $CDCl_3$) δ 7.42 (d, *J* = 8.7 Hz, 2H), 7.33 (t, *J* = 7.6 Hz, 1H), 7.19 (dd, *J* = 8.1, 5.1 Hz, 2H), 7.13 – 7.09 (m, 2H), 7.02 (d, *J* = 7.8 Hz, 1H), 6.92 (t, *J* = 5.8 Hz, 2H), 6.75 (d, *J* = 7.2 Hz, 1H), 3.79 (s, 3H), 2.40 – 2.34 (m, 4H), 1.78 – 1.71 (m, 2H), 1.62 (d, *J* = 12.4 Hz, 2H), 1.50 (s, 3H), 1.14 – 1.04 (m, 3H). ^{13}C NMR (151 MHz, $CDCl_3$) δ 197.4, 159.6, 153.7, 147.1, 139.8, 139.0, 138.3, 133.1, 132.5, 131.0, 130.4, 129.7, 129.5, 128.5, 128.5, 125.8, 125.1, 119.3, 113.3, 55.2, 36.0, 31.0, 26.5, 25.7, 21.6. HRMS (ESI) calcd for $C_{29}H_{28}NaO_2$ $[M+Na]^+$ 431.1987, found 431.1977.

3-(3-Bromophenyl)-2-cyclohexyl-7-phenyl-1*H*-inden-1-one (3h)

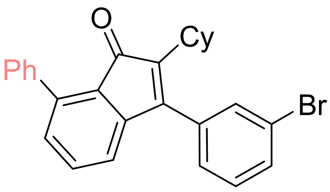

3h

The product purified by flash column chromatography on silica gel (petroleum ether/EtOAc = 30:1) to afford the **3h** as a yellow oil (53 mg, 60% yield). ^{1}H NMR (600 MHz, $CDCl_3$) δ 7.64 – 7.57 (m, 1H), 7.55 – 7.50 (m, 3H), 7.47 – 7.43 (m, 2H), 7.43 – 7.38 (m, 2H), 7.31 – 7.28 (m, 2H), 7.12 (dd, *J* = 7.8, 0.6 Hz, 1H), 6.82 (dd, *J* = 7.2, 0.6 Hz, 1H), 2.39 (tt, *J* = 12.1, 3.4 Hz, 1H), 1.81 – 1.73 (m, 2H), 1.70 (dd, *J* = 9.6, 2.3 Hz, 2H), 1.60 (d, *J* = 7.7 Hz, 1H), 1.54 (d, *J* = 13.6 Hz, 2H), 1.17 (q, *J* = 12.2 Hz, 3H). ^{13}C NMR (151 MHz, $CDCl_3$) δ 196.8, 151.9, 146.5, 140.4, 139.8, 137.3, 135.3, 132.8, 131.8, 131.2, 130.8, 130.3, 129.0, 128.2, 127.9, 126.7, 125.7, 122.8, 119.4, 36.1, 31.0, 26.5, 25.6. HRMS (ESI) calcd for $C_{27}H_{23}BrNaO$ $[M+Na]^+$ 465.0830, found 465.0819.

2-Cyclohexyl-3-(3,4-dimethylphenyl)-7-(4-methoxyphenyl)-1*H*-inden-1-one (3i)

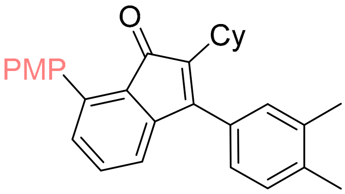

3i

The product purified by flash column chromatography on silica gel (petroleum

ether/EtOAc = 30:1) to afford the **3i** as a yellow solid (53 mg, 63% yield). ^{1}H NMR (600 MHz, $CDCl_3$) δ 7.44 (d, *J* = 8.7 Hz, 2H), 7.17 – 7.10 (m, 3H), 7.00 (dd, *J* = 7.9, 0.6 Hz, 1H), 6.92 (d, *J* = 8.7 Hz, 3H), 6.41 – 6.36 (m, 1H), 3.79 (s, 3H), 2.30 (s, 3H), 2.20 – 2.14 (m, 1H), 2.10 (s, 3H), 1.58 (d, *J* = 9.2 Hz, 4H), 1.43 (d, *J* = 12.5 Hz, 2H), 1.27 – 1.17 (m, 2H), 1.04 (s, 2H). ^{13}C NMR (151 MHz, $CDCl_3$) δ 197.5, 159.6, 155.1, 147.7, 139.6, 139.4, 137.4, 134.1, 133.4, 132.8, 130.8, 130.4, 129.9, 129.6, 125.9, 125.7, 125.4, 119.2, 113.3, 55.2, 36.0, 30.9, 30.6, 26.5, 26.5, 25.7, 20.4, 17.1. HRMS (ESI) calcd for $C_{30}H_{30}NaO_2$ $[M+Na]^+$ 445.2143, found 445.2131.

2-Cyclohexyl-3-(2,3-dimethylphenyl)-7-(4-methoxyphenyl)-1*H*-inden-1-one (**3j**)

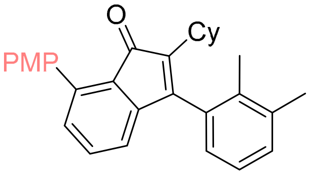

3j

The product purified by flash column chromatography on silica gel (petroleum ether/EtOAc = 20:1) to afford the **3j** as a yellow solid (50 mg, 58% yield). ^{1}H NMR (600 MHz, $CDCl_3$) δ 7.44 (d, *J* = 8.6 Hz, 2H), 7.17 – 7.11 (m, 3H), 7.01 (d, *J* = 7.9 Hz, 1H), 6.95 – 6.88 (m, 3H), 6.39 (d, *J* = 7.1 Hz, 1H), 3.80 (s, 3H), 2.30 (s, 3H), 2.17 (dd, *J* = 13.5, 10.1 Hz, 1H), 2.10 (s, 3H), 1.58 (d, *J* = 9.1 Hz, 3H), 1.43 (d, *J* = 12.6 Hz, 4H), 1.04 (s, 3H). ^{13}C NMR (151 MHz, $CDCl_3$) δ 197.5, 159.6, 155.1, 147.7, 139.6, 139.4, 137.4, 134.1, 133.4, 132.8, 130.8, 130.4, 129.9, 129.6, 125.9, 125.7, 125.4, 119.2, 113.3, 55.2, 36.0, 30.9, 30.6, 26.5, 26.5, 25.7, 20.4, 17.1. HRMS (ESI) calcd for $C_{30}H_{30}NaO_2$ $[M+Na]^+$ 445.2143, found 445.2136.

2-Cyclohexyl-3-(3,5-dimethylphenyl)-7-(4-methoxyphenyl)-1*H*-inden-1-one (**3k**)

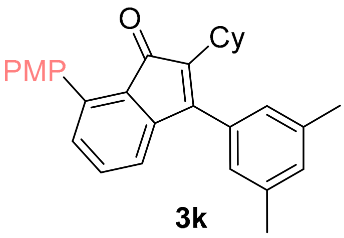

3k

The product purified by flash column chromatography on silica gel (petroleum

ether/EtOAc = 30:1) to afford the **3k** as a yellow solid (60 mg, 71% yield). ^{1}H NMR (600 MHz, $CDCl_3$) δ 7.43 – 7.39 (m, 2H), 7.18 (t, 1H), 7.01 (d, *J* = 7.3 Hz, 2H), 6.92 – 6.89 (m, 4H), 6.75 (d, *J* = 7.1 Hz, 1H), 3.79 (s, 3H), 2.39 – 2.36 (m, 1H), 2.33 (s, 6H), 1.79 – 1.72 (m, 2H), 1.62 (d, *J* = 12.5 Hz, 2H), 1.52 (d, *J* = 8.9 Hz, 1H), 1.46 (s, 2H), 1.13 – 1.05 (m, 3H). ^{13}C NMR (151 MHz, $CDCl_3$) δ 196.5, 158.5, 152.9, 146.1, 138.7, 137.8, 137.1, 132.0, 131.5, 129.9, 129.3, 128.7, 124.7, 124.6, 118.3, 112.2, 54.2, 35.0, 29.9, 25.5, 24.7, 20.4. HRMS (ESI) calcd for $C_{30}H_{30}NaO_2$ $[M+Na]^+$ 445.2143, found 445.2133.

2-Cyclohexyl-3,7-diphenyl-1*H*-inden-1-one (3l)

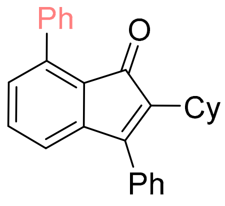

3l

The product purified by flash column chromatography on silica gel (petroleum ether/EtOAc = 20:1) to afford the **3m** as a yellow solid (50 mg, 68% yield). ^{1}H NMR (600 MHz, $CDCl_3$) δ 7.44 (dd, *J* = 11.3, 4.4 Hz, 4H), 7.39 – 7.35 (m, 3H), 7.33 – 7.30 (m, 3H), 7.19 (t, 1H), 7.02 (dd, *J* = 7.8, 0.7 Hz, 1H), 6.77 (dd, *J* = 7.2, 0.6 Hz, 1H), 2.36 (tt, *J* = 12.1, 7.8, 3.4 Hz, 1H), 1.72 (qd, *J* = 12.2, 6.0 Hz, 2H), 1.61 (d, *J* = 12.4 Hz, 2H), 1.48 (t, *J* = 12.5 Hz, 3H), 1.12 – 1.04 (m, 3H). ^{13}C NMR (151 MHz, $CDCl_3$) δ 197.2, 153.7, 147.0, 140.1, 139.2, 137.4, 133.2, 132.6, 131.0, 129.1, 128.8, 128.7, 128.1, 128.0, 127.9, 119.6, 36.0, 31.0, 26.6, 25.7.

2-Cyclohexyl-7-(4-ethoxyphenyl)-3-phenyl-1*H*-inden-1-one (3m)

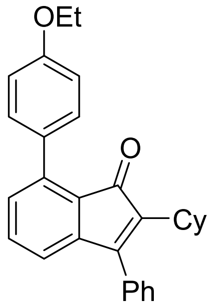

3m

The product purified by flash column chromatography on silica gel (petroleum ether/EtOAc = 30:1) to afford the **3m** as a yellow oil (62 mg, 76% yield). ^{1}H NMR (600 MHz, $CDCl_3$) δ 7.55 – 7.43 (m, 5H), 7.40 – 7.35 (m, 2H), 7.25 (t, *J* = 3.8 Hz, 1H), 7.09 (dd, *J* = 7.9, 0.7 Hz, 1H), 7.00 – 6.95 (m, 2H), 6.80 (dd, *J* = 7.2, 0.7 Hz, 1H), 4.10 (q, *J* = 7.0 Hz, 2H), 2.44 (tt, *J* = 12.1, 3.4 Hz, 1H), 1.86 – 1.77 (m, 2H), 1.69 (d, *J* = 12.5 Hz, 2H), 1.59 (d, *J* = 8.9 Hz, 1H), 1.53 (s, 2H),

1.44 (t, *J* = 7.0 Hz, 3H), 1.20 – 1.10 (m, 3H). ^{13}C NMR (151 MHz, $CDCl_3$) δ 197.3, 159.1, 153.5, 147.0, 140.0, 139.1, 133.2, 132.6, 131.0, 130.4, 129.6, 129.5, 128.7, 128.6, 128.0, 125.7, 119.2, 113.8, 63.4, 36.0, 31.0, 26.5, 25.7, 14.9. HRMS (ESI) calcd for $C_{29}H_{28}NaO_2$ [M+Na]$^+$ 431.1987, found 431.1971.

7-(3-Chlorophenyl)-2-cyclohexyl-3-phenyl-1*H*-inden-1-one (3n)

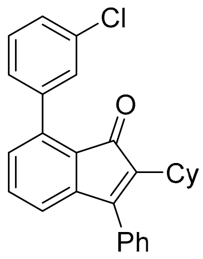

3n

The product purified by flash column chromatography on silica gel (petroleum ether/EtOAc = 30:1) to afford the **3n** as a yellow oil (49 mg, 61% yield). ^{1}H NMR (600 MHz, $CDCl_3$) δ 7.52 (t, *J* = 7.4 Hz, 2H), 7.46 (d, *J* = 11.0 Hz, 2H), 7.42 (dd, *J* = 5.0, 2.0 Hz, 1H), 7.40 – 7.34 (m, 4H), 7.28 (t, *J* = 7.6 Hz, 1H), 7.06 (d, *J* = 7.8 Hz, 1H), 6.87 (d, *J* = 7.2 Hz, 1H), 2.48 – 2.41 (m, 1H), 1.84 – 1.74 (m, 2H), 1.70 (d, *J* = 12.1 Hz, 2H), 1.59 (d, *J* = 9.8 Hz, 1H), 1.54 (s, 2H), 1.21 – 1.10 (m, 3H). ^{13}C NMR (151 MHz, $CDCl_3$) δ 197.0, 153.8, 147.0, 139.3, 139.2, 138.4, 133.8, 133.0, 132.8, 130.6, 129.0, 128.9, 128.8, 128.7, 128.1, 128.0, 127.5, 126.1, 120.0, 36.0, 31.0, 26.5, 25.7. HRMS (ESI) calcd for $C_{27}H_{23}ClNaO$ [M+Na]$^+$ 421.1335, found 421.1324.

7-(3-Chloro-4-methoxyphenyl)-2-cyclohexyl-3-phenyl-1*H*-inden-1-one (3o)

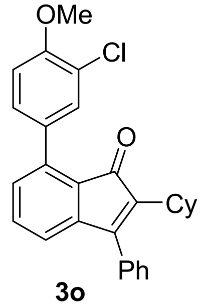

3o

The product purified by flash column chromatography on silica gel (petroleum ether/EtOAc = 30:1) to afford the **3o** as a yellow solid (53 mg, 62% yield). ^{1}H NMR (600 MHz, $CDCl_3$) δ 7.54 – 7.50 (m, 3H), 7.48 – 7.45 (m, 2H), 7.40 – 7.37 (m, 2H), 7.26 (d, *J* = 2.5 Hz, 1H), 7.06 (d, *J* = 7.4 Hz, 1H), 7.01 (d, *J* = 8.5 Hz, 1H), 6.85 – 6.82 (m, 1H), 3.96 (s, 3H), 2.44 (tt, *J* = 12.1, 3.4 Hz, 1H), 1.83 – 1.76 (m, 2H), 1.70 (d, *J* = 12.5 Hz, 2H), 1.55 (d, *J* = 13.4 Hz, 2H), 1.23 – 1.12 (m, 4H). ^{13}C NMR

(151 MHz, $CDCl_3$) δ 197.2, 154.9, 153.7, 147.1, 139.2, 138.4, 133.1, 132.7, 130.7, 130.6, 130.6, 128.9, 128.8, 128.7, 128.0, 125.9, 121.9, 119.7, 111.2, 56.1, 36.0, 31.0, 26.5, 25.7. HRMS (ESI) calcd for $C_{28}H_{25}ClNaO_2$ $[M+Na]^+$ 451.1441, found 451.1436.

2-Cyclohexyl-7-(4-methoxy-2-methylphenyl)-3-phenyl-1*H*-inden-1-one (**3p**)

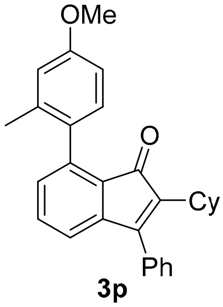

3p

The product purified by flash column chromatography on silica gel (petroleum ether/EtOAc = 30:1) to afford the **3p** as a yellow solid (60 mg, 73% yield). ^1H NMR (600 MHz, $CDCl_3$) δ 7.52 (dd, *J* = 9.3, 5.5 Hz, 2H), 7.48 – 7.44 (m, 1H), 7.41 – 7.39 (m, 2H), 7.24 (d, *J* = 7.5 Hz, 1H), 7.11 (d, *J* = 8.3 Hz, 1H), 6.98 – 6.95 (m, 1H), 6.87 – 6.83 (m, 2H), 6.80 (dd, *J* = 8.3, 2.6 Hz, 1H), 3.84 (s, 3H), 2.42 (tt, *J* = 12.1, 3.4 Hz, 1H), 2.16 (s, 3H), 1.81 – 1.76 (m, 2H), 1.68 (d, *J* = 9.5 Hz, 2H), 1.53 (s, 2H), 1.25 (s, 2H), 1.14 (d, *J* = 9.9 Hz, 2H). ^{13}C NMR (151 MHz, $CDCl_3$) δ 197.5, 159.1, 153.7, 146.3, 139.1, 138.9, 137.2, 133.2, 132.3, 131.5, 130.3, 130.0, 128.7, 128.6, 128.0, 127.1, 119.3, 115.2, 110.8, 55.1, 35.9, 30.9, 26.5, 25.7, 20.4. HRMS (ESI) calcd for $C_{29}H_{28}NaO_2$ $[M+Na]^+$ 431.1987, found 431.1975.

2-Cyclohexyl-7-(4-methoxy-3-methylphenyl)-3-phenyl-1*H*-inden-1-one (**3q**)

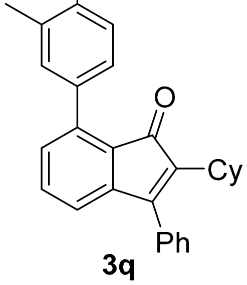

3q

The product purified by flash column chromatography on silica gel (petroleum ether/EtOAc = 30:1) to afford the **3q** as a yellow solid (57 mg, 70% yield). ^1H NMR (600 MHz, $CDCl_3$) δ 7.52 (t, *J* = 7.5 Hz, 2H), 7.45 (t, *J* = 7.4 Hz, 1H), 7.42 – 7.35 (m, 3H), 7.29 (s, 1H), 7.24 (t, *J* = 7.6 Hz, 1H), 7.09 (d, *J* = 7.9 Hz, 1H), 6.91 (d, *J* = 8.4 Hz,

1H), 6.80 (d, *J* = 7.2 Hz, 1H), 3.89 (s, 3H), 2.44 (tt, *J* = 12.1, 3.5 Hz, 1H), 2.29 (d, *J* = 5.3 Hz, 3H), 1.84 – 1.76 (m, 2H), 1.69 (d, *J* = 12.1 Hz, 2H), 1.60 (s, 1H), 1.54 (d, *J* = 13.4 Hz, 2H), 1.20 – 1.12 (m, 3H). ^{13}C NMR (151 MHz, $CDCl_3$) δ 197.3, 157.8, 153.4, 147.0, 140.2, 139.1, 133.3, 132.5, 131.2, 131.1, 129.2, 128.7, 128.6, 128.1, 128.0, 126.0, 125.7, 119.1, 109.1, 55.3, 36.0, 31.0, 26.6, 25.7, 16.3. HRMS (ESI) calcd for $C_{29}H_{28}NaO_2$ $[M+Na]^+$ 431.1987, found 431.1975.

7-(4-Chloro-3-methylphenyl)-2-cyclohexyl-3-phenyl-1*H*-inden-1-one (3r)

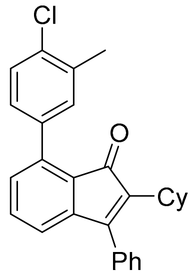

3r

The product purified by flash column chromatography on silica gel (petroleum ether/EtOAc = 30:1) to afford the **3r** as a yellow oil (50 mg, 61% yield). ^{1}H NMR (600 MHz, $CDCl_3$) δ 7.51 (t, *J* = 7.4 Hz, 2H), 7.46 (d, *J* = 7.3 Hz, 1H), 7.39 – 7.33 (m, 2H), 7.25 – 7.23 (m, 2H), 7.11 – 7.04 (m, 3H), 6.81 (d, *J* = 7.2 Hz, 1H), 2.47 – 2.41 (m, 1H), 2.38 (s, 3H), 1.78 (dt, *J* = 22.0, 7.7 Hz, 2H), 1.70 – 1.68 (m, 2H), 1.59 – 1.53 (m, 3H), 1.20 – 1.13 (m, 3H). ^{13}C NMR (151 MHz, $CDCl_3$) δ 197.0, 153.5, 146.9, 140.4, 139.1, 137.5, 137.2, 133.3, 132.4, 131.1, 129.7, 128.7, 128.6, 128.0, 126.8, 126.0, 119.4, 35.9, 31.0, 26.6, 25.7, 21.4.

2-Cyclohexyl-7-(2,5-dimethylphenyl)-3-phenyl-1*H*-inden-1-one (3s)

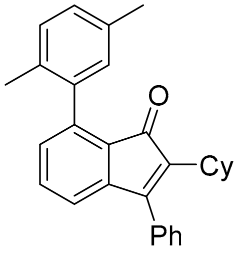

3s

The product purified by flash column chromatography on silica gel (petroleum ether/EtOAc = 30:1) to afford the **3s** as a yellow oil (53 mg, 68% yield). ^{1}H NMR (600 MHz, $CDCl_3$) δ 7.52 (t, *J* = 7.4 Hz, 2H), 7.48 – 7.44 (m, 1H), 7.42 – 7.38 (m, 2H), 7.26 (t, *J* = 7.5 Hz, 1H), 7.16 (d, *J* = 7.7 Hz, 1H), 7.11 (dd, *J* = 7.7, 1.2 Hz, 1H), 6.98 – 6.94 (m, 2H), 6.86 (dd, *J* = 7.3, 0.7 Hz, 1H), 2.42 (tt, *J* = 12.1, 3.4 Hz, 1H),

2.34 (s, 3H), 2.11 (s, 3H), 1.82 – 1.74 (m, 2H), 1.68 (s, 2H), 1.56 (s, 1H), 1.51 (d, *J* = 11.5 Hz, 2H), 1.18 – 1.09 (m, 3H). ^{13}C NMR (151 MHz, $CDCl_3$) δ 197.3, 153.7, 146.2, 139.2, 139.0, 137.8, 134.7, 133.2, 132.6, 132.4, 131.0, 129.5, 129.3, 128.7, 128.6, 128.6, 128.0, 127.0, 119.4, 35.9, 30.9, 30.9, 26.5, 25.7, 21.0, 19.5. HRMS (ESI) calcd for $C_{29}H_{28}NaO$ $[M+Na]^+$ 415.2038, found 415.2025.

2-Cyclohexyl-7-(4-methoxyphenyl)-5-methyl-3-phenyl-1*H*-inden-1-one (**3t**)

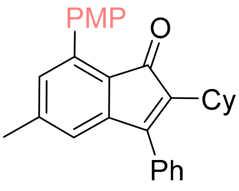

3t

The product purified by flash column chromatography on silica gel (petroleum ether/EtOAc = 30:1) to afford the **3t** as a yellow solid (61 mg, 75% yield). ^{1}H NMR (600 MHz, $CDCl_3$) δ 7.52 (dd, *J* = 10.2, 4.6 Hz, 2H), 7.50 – 7.45 (m, 3H), 7.39 – 7.36 (m, 2H), 6.98 (dd, 2H), 6.89 (s, 1H), 6.61 (s, 1H), 3.86 (s, 3H), 2.42 (tt, *J* = 12.1, 3.3 Hz, 1H), 2.29 (s, 3H), 1.83 – 1.76 (m, 2H), 1.69 (d, *J* = 12.5 Hz, 2H), 1.56 – 1.52 (m, 2H), 1.24 – 1.07 (m, 4H). ^{13}C NMR (151 MHz, $CDCl_3$) δ 197.0, 159.6, 153.1, 147.6, 143.4, 139.9, 139.6, 133.4, 130.9, 130.3, 129.8, 128.6, 128.6, 128.1, 123.4, 120.6, 113.3, 55.2, 36.0, 31.0, 26.6, 25.7, 21.8. HRMS (ESI) calcd for $C_{29}H_{28}NaO_2$ $[M+Na]^+$ 431.1987, found 431.1976.

2-Cyclohexyl-5-fluoro-7-(4-methoxyphenyl)-3-phenyl-1*H*-inden-1-one (**3u**)

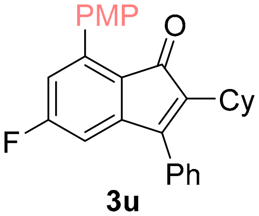

3u

The product purified by flash column chromatography on silica gel (petroleum ether/EtOAc = 30:1) to afford the **3u** as a yellow solid (58 mg, 70% yield). ^{1}H NMR (600 MHz, $CDCl_3$) δ 7.53 (t, *J* = 7.4 Hz, 2H), 7.50 – 7.44 (m, 3H), 7.36 (dd, *J* = 5.1, 3.2 Hz, 2H), 7.01 – 6.96 (m, 2H), 6.75 (dd, *J* = 10.0, 2.2 Hz, 1H), 6.53 (dd, *J* = 8.1, 2.2 Hz, 1H), 3.86 (s,

3H), 2.45 (tt, *J* = 12.1, 3.4 Hz, 1H), 1.84 – 1.75 (m, 2H), 1.70 (d, *J* = 12.3 Hz, 2H), 1.59 (d, *J* = 10.1 Hz, 1H), 1.54 (s, 2H), 1.19 – 1.10 (m, 3H). ^{13}C NMR (151 MHz, $CDCl_3$) δ 195.6, 165.5 (d, *J* = 254.3 Hz), 160.0, 151.6, 150.7 (d, *J* = 9.4 Hz), 142.3, 142.2, 140.7, 132.6, 130.3, 128.9, 128.8, 128.6, 128.0, 121.7, 115.7 (d, *J* = 22.5 Hz), 113.4, 108.1 (d, *J* = 25.3 Hz), 55.3, 36.1, 31.0, 26.5, 25.7. HRMS (ESI) calcd for $C_{28}H_{25}FNaO_2$ $[M+Na]^+$ 435.1736, found 435.1736.

5-Chloro-2-cyclohexyl-7-(4-methoxyphenyl)-3-phenyl-1*H*-inden-1-one (**3v**)

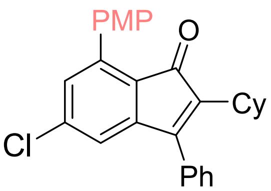

3v

The product purified by flash column chromatography on silica gel (petroleum ether/EtOAc = 30:1) to afford the **3v** as a yellow solid (53 mg, 62% yield). ^{1}H NMR (600 MHz, $CDCl_3$) δ 7.54 (dd, *J* = 10.2, 4.6 Hz, 2H), 7.49 – 7.46 (m, 3H), 7.36 (dd, *J* = 5.2, 3.2 Hz, 2H), 7.10 (d, *J* = 1.7 Hz, 1H), 7.00 – 6.96 (m, 2H), 6.77 (d, *J* = 1.7 Hz, 1H), 3.87 (s, 3H), 2.44 (tt, *J* = 12.1, 3.4 Hz, 1H), 1.83 – 1.75 (m, 2H), 1.70 (d, *J* = 12.2 Hz, 2H), 1.54 (d, *J* = 13.4 Hz, 2H), 1.23 – 1.09 (m, 4H). ^{13}C NMR (151 MHz, $CDCl_3$) δ 195.9, 160.0, 152.5, 149.2, 141.1, 140.5, 138.6, 132.6, 130.4, 130.0, 129.0, 128.8, 128.4, 128.0, 123.9, 119.8, 113.4, 55.3, 36.1, 30.9, 26.5, 25.7. HRMS (ESI) calcd for $C_{28}H_{25}ClNaO_2$ $[M+Na]^+$ 451.1441, found 451.1426.

2-Cyclohexyl-4-methoxy-7-(4-methoxyphenyl)-3-phenyl-1*H*-inden-1-one (**3w**)

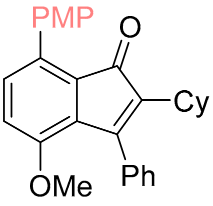

3w

The product purified by flash column chromatography on silica gel (petroleum ether/EtOAc = 50:1) to afford the **3w** as a yellow oil (62 mg, 73% yield). ^{1}H NMR (600 MHz,

$CDCl_3$) δ 7.44 – 7.40 (m, 4H), 7.39 – 7.35 (m, 1H), 7.32 – 7.29 (m, 2H), 7.05 (d, *J* = 8.6 Hz, 1H), 6.98 – 6.95 (m, 2H), 6.90 (d, *J* = 8.6 Hz, 1H), 3.86 (s, 3H), 3.49 (s, 3H), 2.25 (tt, *J* = 12.2, 3.5 Hz, 1H), 1.76 – 1.72 (m, 2H), 1.64 (d, *J* = 12.9 Hz, 2H), 1.53 (s, 1H), 1.49 – 1.45 (m, 2H), 1.15 – 1.03 (m, 3H). ^{13}C NMR (151 MHz, $CDCl_3$) δ 197.3, 159.3, 154.6, 152.3, 138.7, 135.8, 133.4, 133.0, 131.4, 130.3, 129.7, 129.4, 128.2, 127.8, 127.7, 127.5, 127.2, 119.5, 113.3, 55.9, 55.2, 35.7, 30.9, 26.5, 25.7. HRMS (ESI) calcd for $C_{29}H_{28}NaO_3$ $[M+Na]^+$ 447.1936, found 447.1960.

2-Isopropyl-7-(4-methoxyphenyl)-3-phenyl-1*H*-inden-1-one (**3x**)

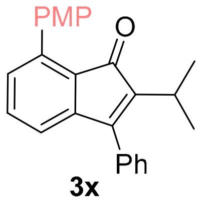

3x

The product purified by flash column chromatography on silica gel (petroleum ether/EtOAc = 50:1) to afford the **3x** as a yellow solid (42 mg, 59% yield). ^1H NMR (600 MHz, $CDCl_3$) δ 7.51 (t, *J* = 8.2 Hz, 4H), 7.45 (t, *J* = 7.4 Hz, 1H), 7.41 – 7.37 (m, 2H), 7.26 (d, *J* = 8.0 Hz, 1H), 7.10 (d, *J* = 7.3 Hz, 1H), 6.99 (d, *J* = 8.7 Hz, 2H), 6.83 (d, *J* = 6.6 Hz, 1H), 3.87 (s, 3H), 2.81 (dt, *J* = 13.9, 7.0 Hz, 1H), 1.20 (d, *J* = 7.0 Hz, 6H). ^{13}C NMR (151 MHz, $CDCl_3$) δ 197.2, 159.7, 153.2, 147.0, 139.9, 139.8, 133.1, 132.6, 131.0, 130.4, 129.6, 128.7, 128.6, 128.0, 125.8, 119.3, 113.3, 55.2, 25.5, 21.4. HRMS (ESI) calcd for $C_{25}H_{22}NaO_2$ $[M+Na]^+$ 377.1517, found 377.1505.

2-(Pentan-2-yl)-3,7-diphenyl-1*H*-inden-1-one (**3y**)

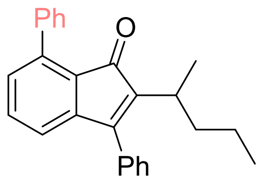

3y

The product purified by flash column chromatography on silica gel (petroleum ether/EtOAc = 50:1) to afford the **3y** as a yellow oil (50 mg, 71% yield). ^1H NMR (600 MHz, $CDCl_3$) δ 7.48 – 7.41 (m, 4H), 7.39 – 7.36 (m, 2H), 7.34 – 7.29 (m, 3H), 7.19 (dd, *J* = 17.2, 9.7 Hz, 2H), 7.07 – 7.02 (m, 1H), 6.76 (dd, *J* = 7.2, 0.5 Hz, 1H), 2.59 – 2.53 (m, 1H), 1.68 – 1.58 (m, 1H), 1.38 – 1.32 (m, 1H),

1.13 – 1.08 (m, 5H), 0.67 (t, *J* = 7.3 Hz, 3H). ^{13}C NMR (151 MHz, $CDCl_3$) δ 197.1, 154.3, 147.0, 140.1, 139.0, 137.4, 133.1, 132.7, 131.0, 129.1, 128.7, 128.7, 128.1, 128.0, 127.9, 126.0, 119.5, 37.2, 30.6, 21.3, 19.8, 13.9.

2-(Pentan-3-yl)-3,7-diphenyl-1*H*-inden-1-one (3z)

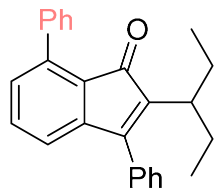

3z

The product purified by flash column chromatography on silica gel (petroleum ether/EtOAc = 50:1) to afford the **3z** as a yellow oil (46 mg, 65% yield). ^{1}H NMR (600 MHz, $CDCl_3$) δ 7.48 – 7.45 (m, 2H), 7.42 (t, *J* = 7.4 Hz, 2H), 7.37 (t, *J* = 7.1 Hz, 3H), 7.33 (d, *J* = 7.3 Hz, 1H), 7.31 – 7.28 (m, 2H), 7.20 (t, *J* = 7.6 Hz, 1H), 7.07 – 7.02 (m, 1H), 6.72 (d, *J* = 7.2 Hz, 1H), 2.27 – 2.20 (m, 1H), 1.69 – 1.59 (m, 2H), 1.48 – 1.42 (m, 2H), 0.72 (t, *J* = 7.5 Hz, 6H). ^{13}C NMR (151 MHz, $CDCl_3$) 197.2, 156.3, 147.2, 140.1, 137.3, 137.2, 133.2, 132.7, 131.1, 129.1, 128.6, 128.6, 128.1, 128.0, 127.9, 125.9, 119.5, 40.2, 26.6, 12.8.

NMR spectra of compound

^1H NMR of **3a** in $CDCl_3$ (600 MHz, $CDCl_3$)

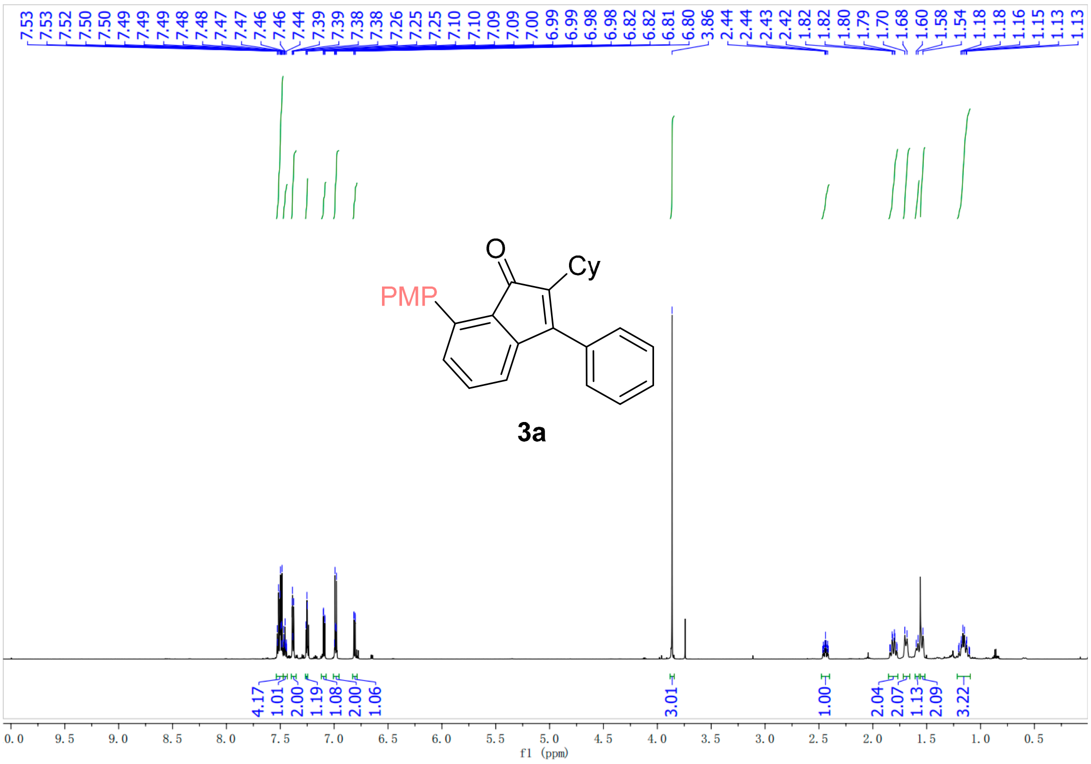

^{13}C NMR of **3a** in $CDCl_3$ (151 MHz, $CDCl_3$)

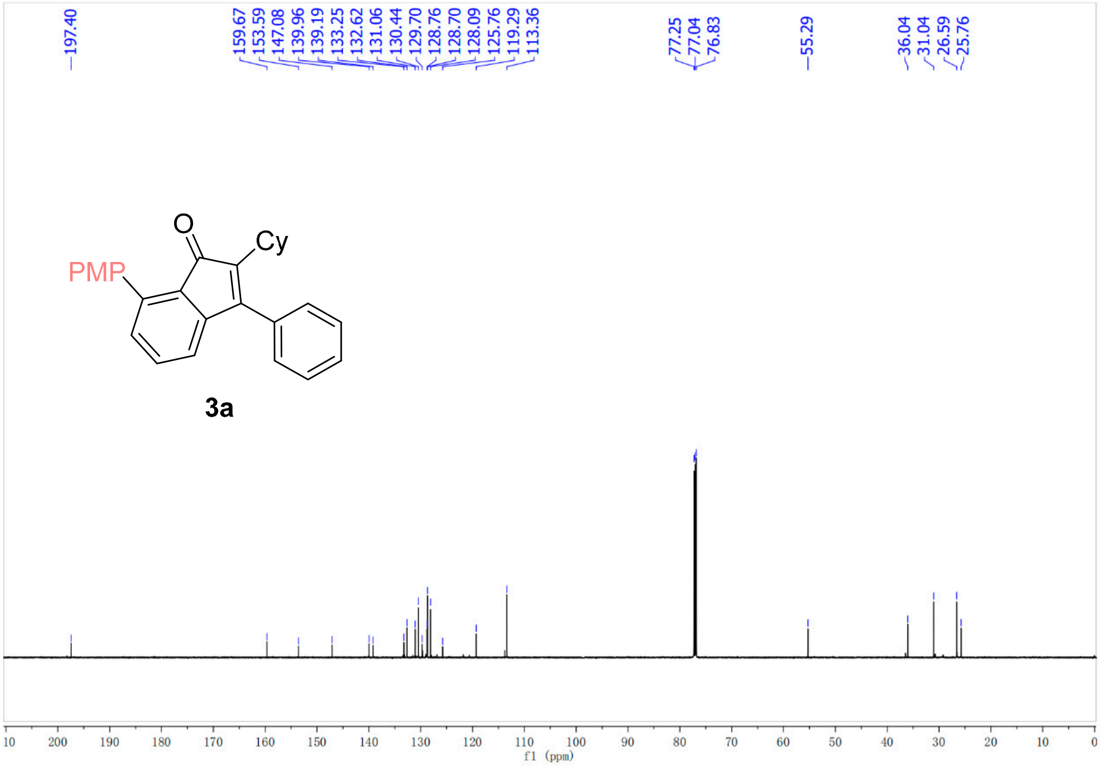

^{1}H NMR of **3b** in $CDCl_3$ (600 MHz, $CDCl_3$)

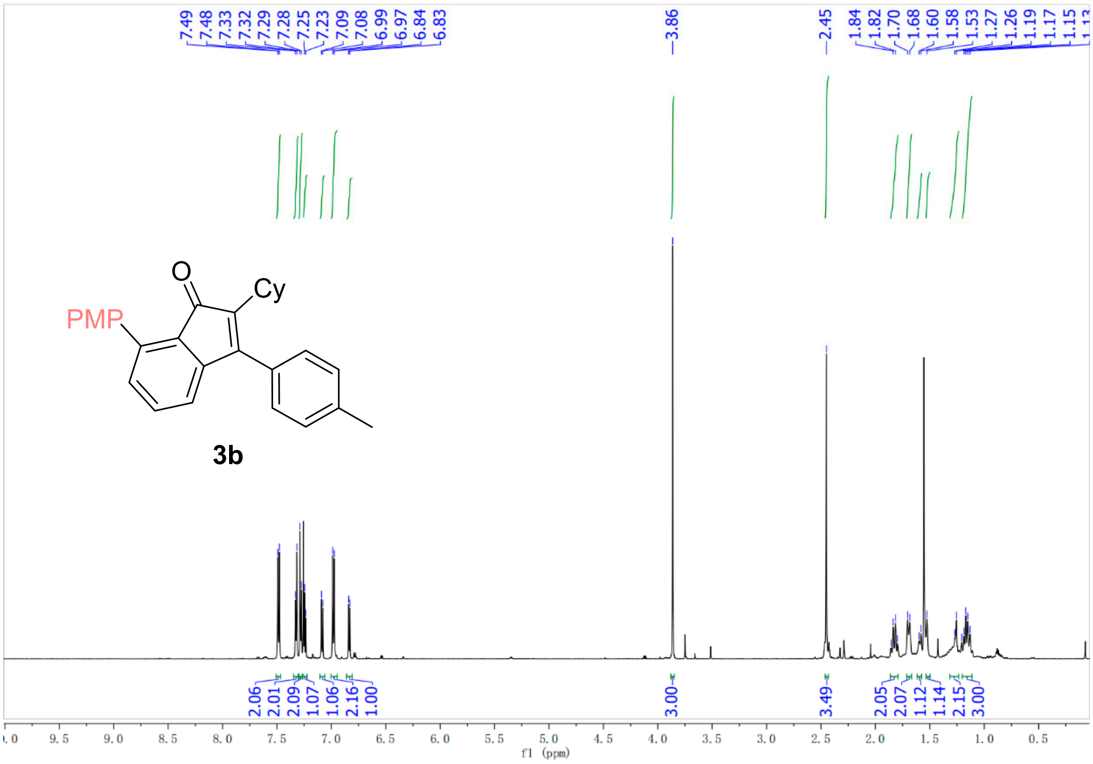

^{13}C NMR of **3b** in $CDCl_3$ (151 MHz, $CDCl_3$)

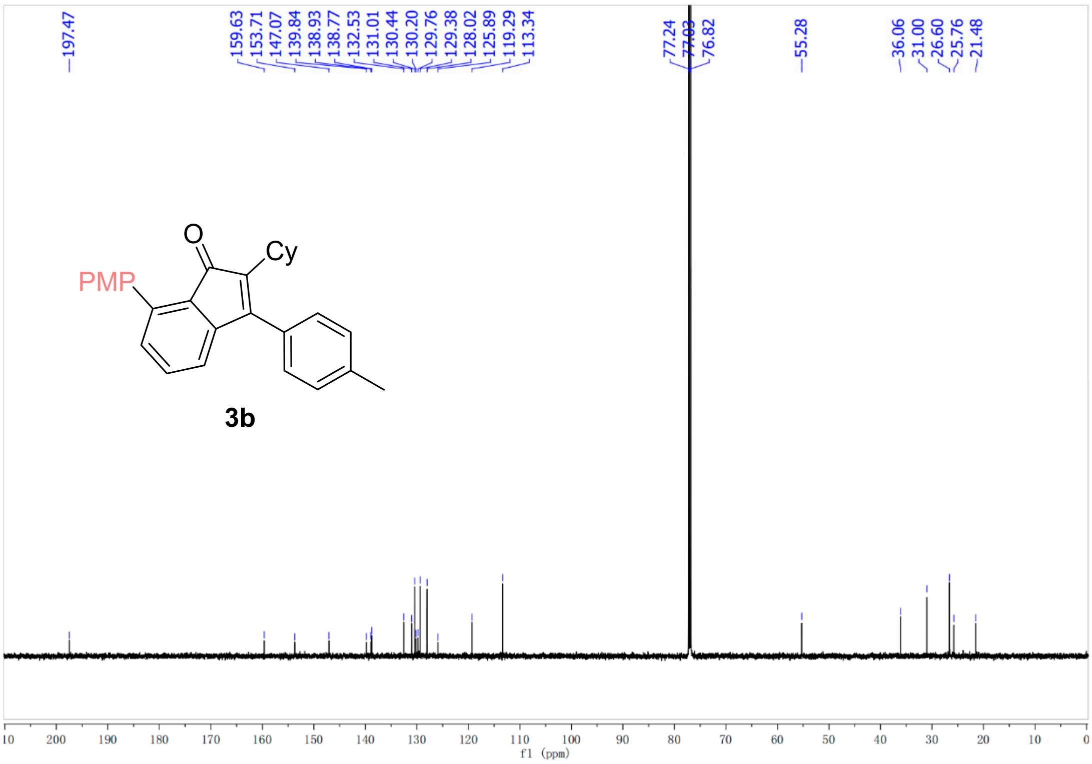

^{1}H NMR of **3c** in $CDCl_3$ (600 MHz, $CDCl_3$)

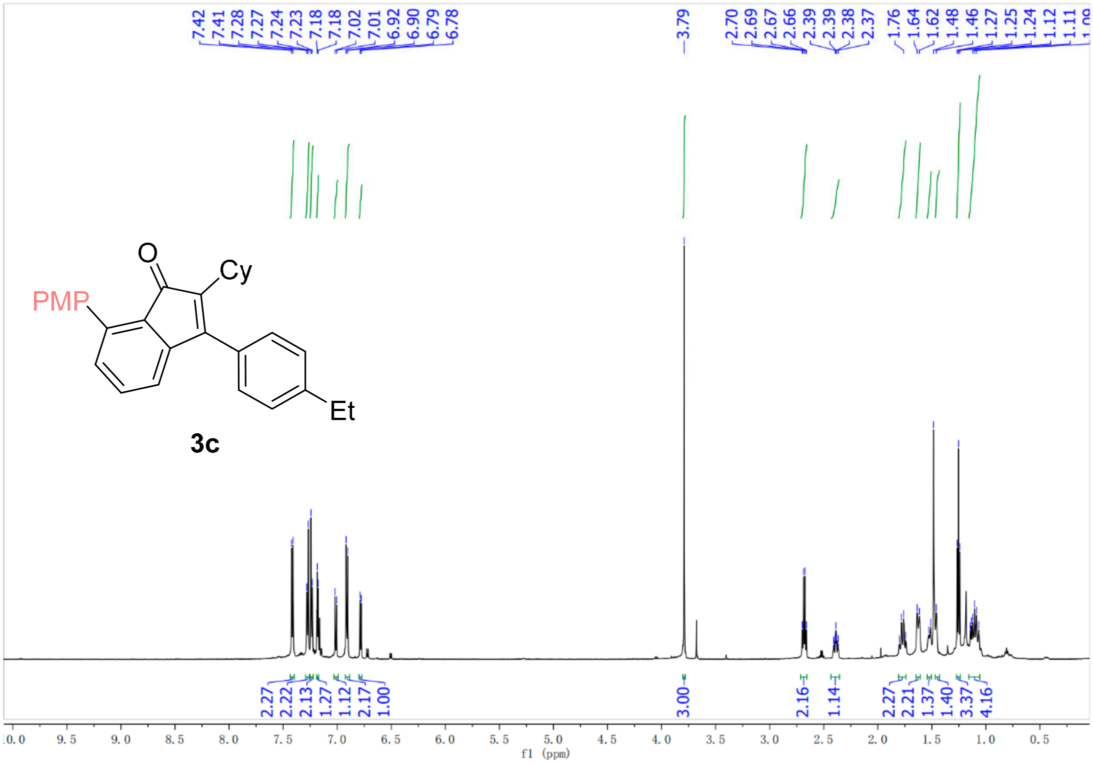

^{13}C NMR of **3c** in $CDCl_3$ (151 MHz, $CDCl_3$)

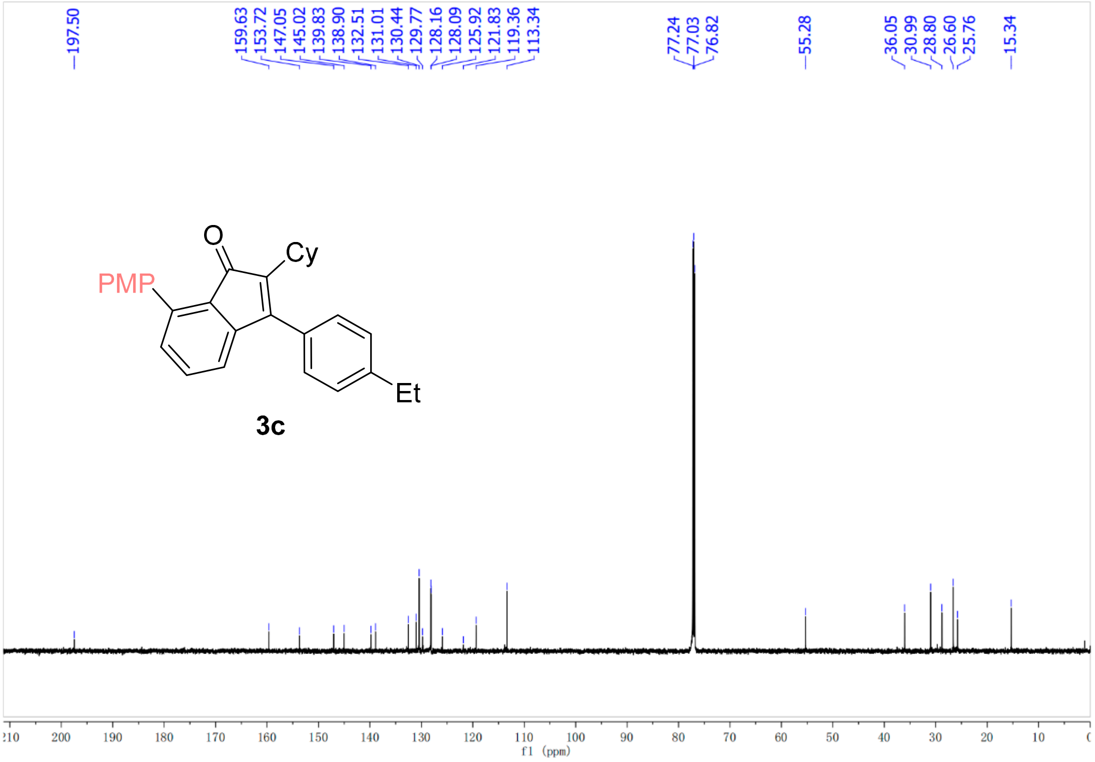

^{1}H NMR of **3d** in $CDCl_3$ (600 MHz, $CDCl_3$)

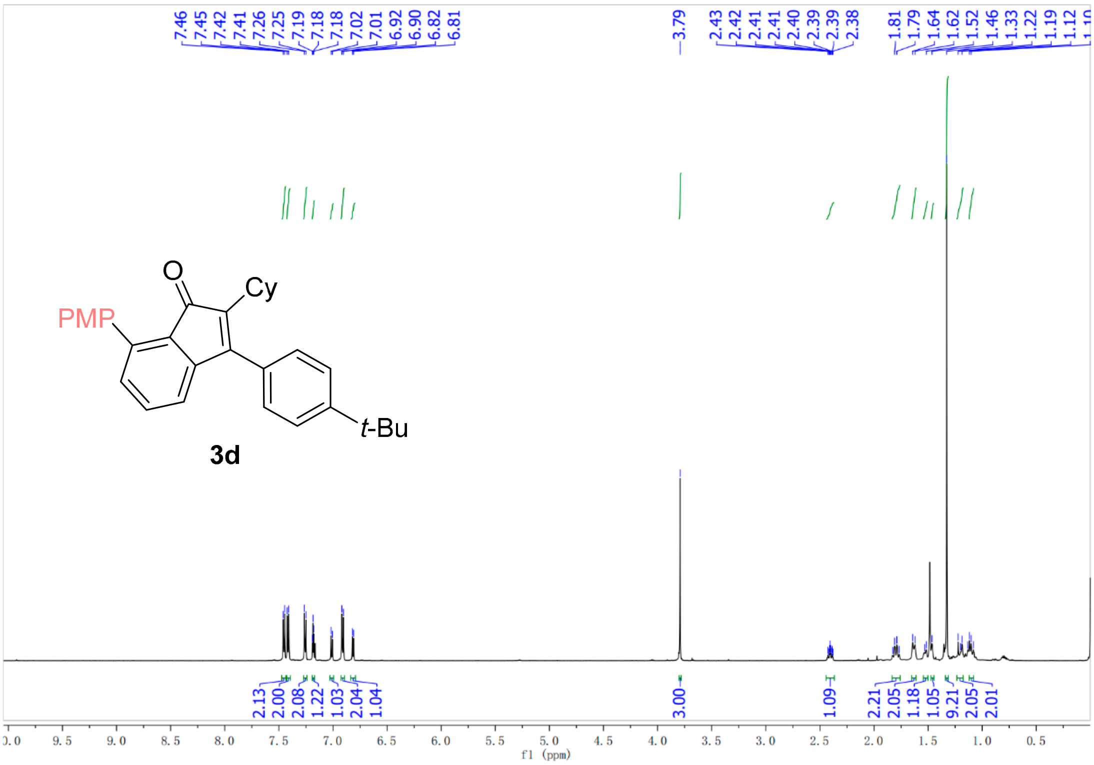

^{13}C NMR of **3d** in $CDCl_3$ (151 MHz, $CDCl_3$)

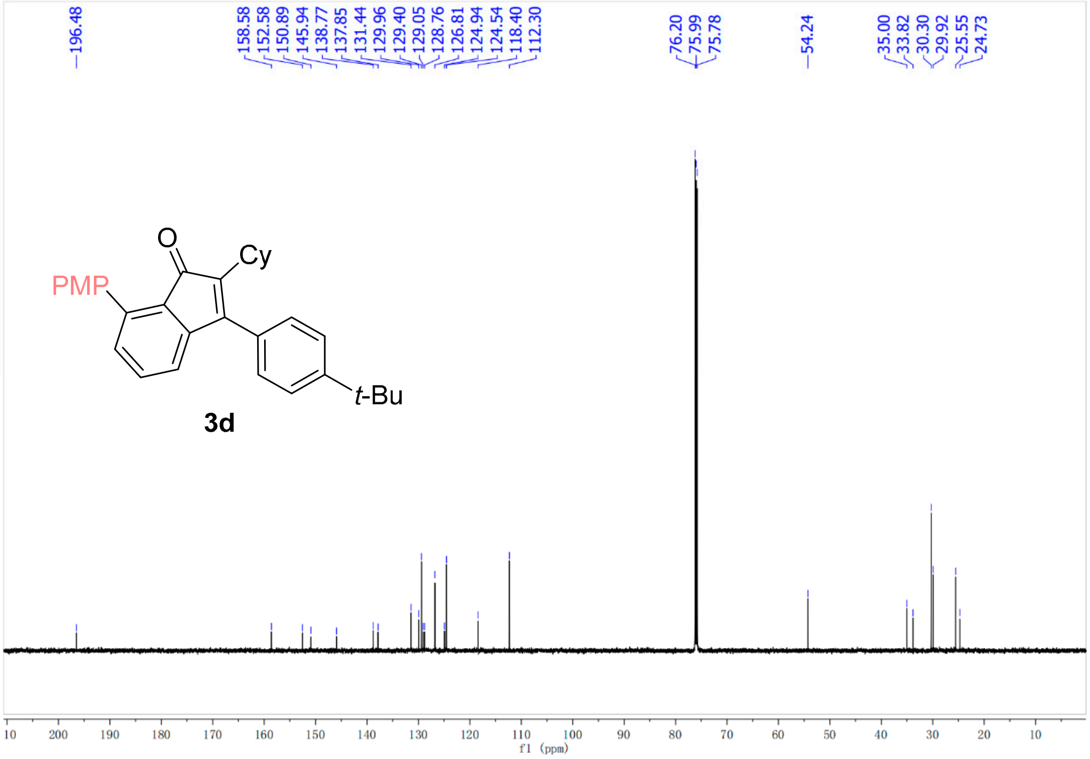

^{1}H NMR of **3e** in $CDCl_3$ (600 MHz, $CDCl_3$)

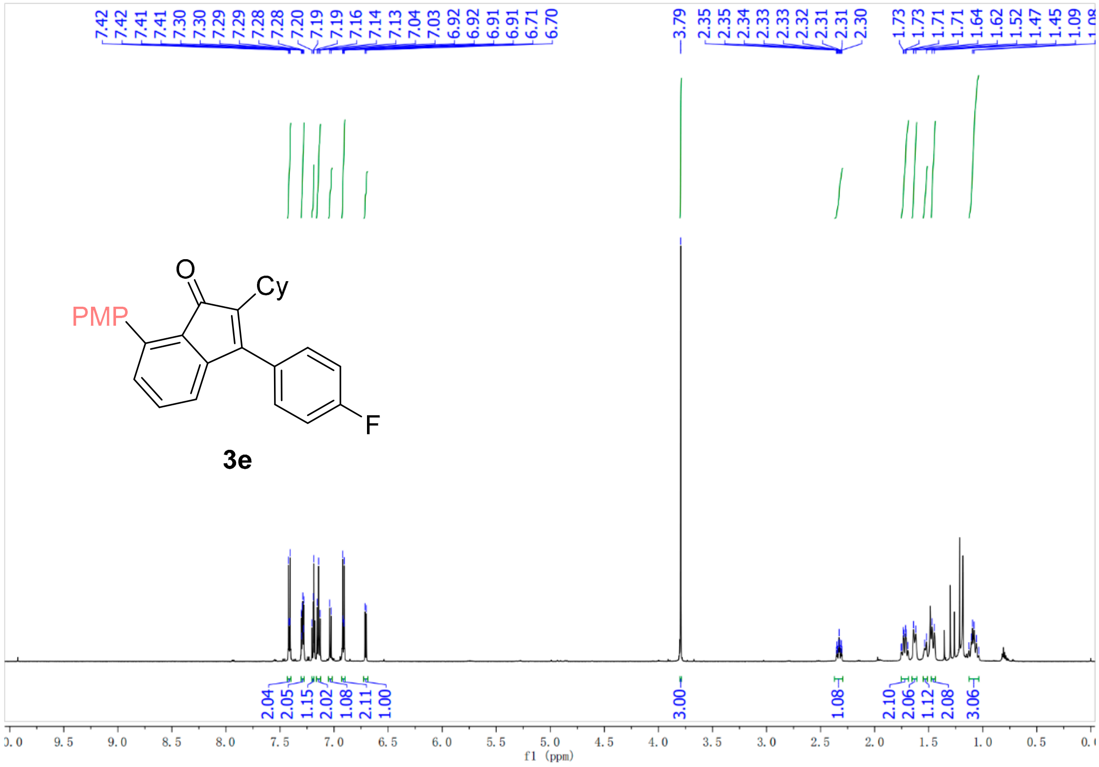

^{13}C NMR of **3e** in $CDCl_3$ (151 MHz, $CDCl_3$)

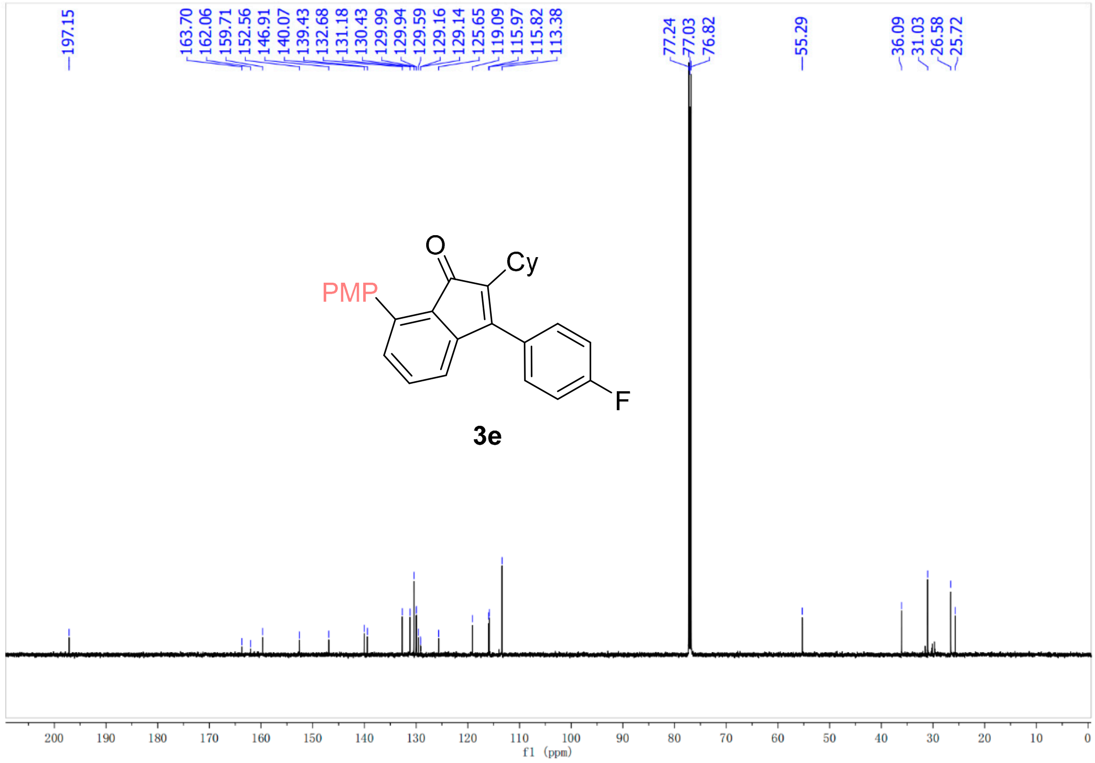

^{1}H NMR of **3f** in $CDCl_3$ (600 MHz, $CDCl_3$)

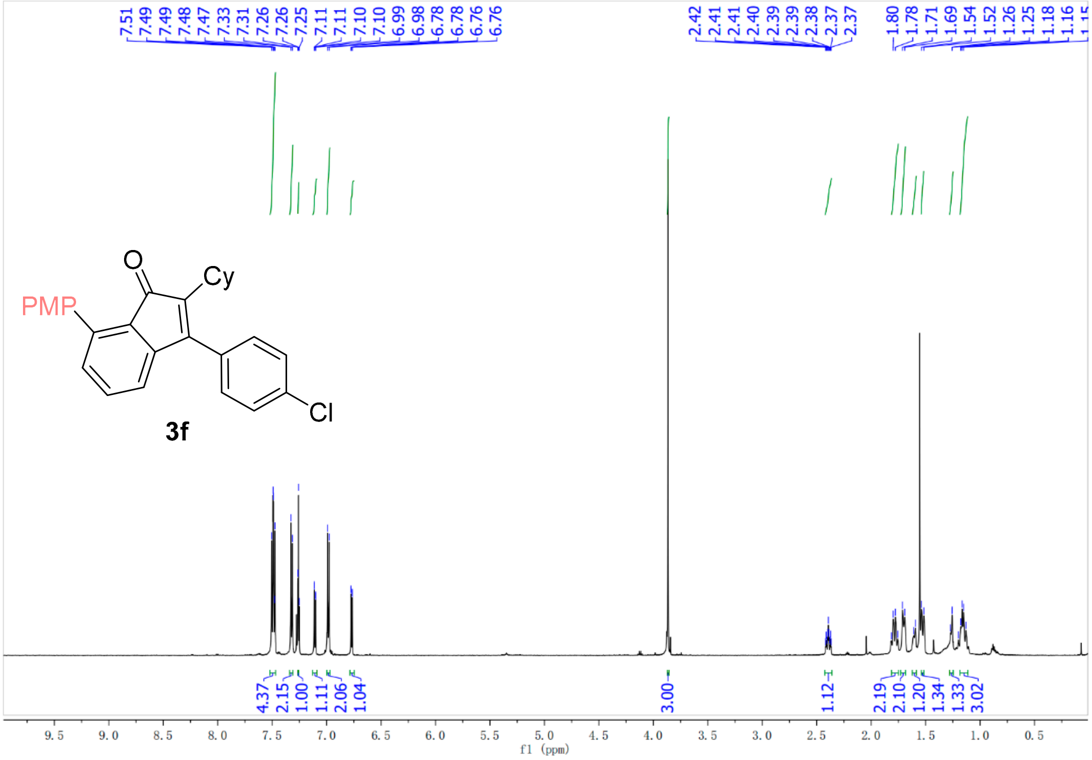

^{13}C NMR of **3f** in $CDCl_3$ (151 MHz, $CDCl_3$)

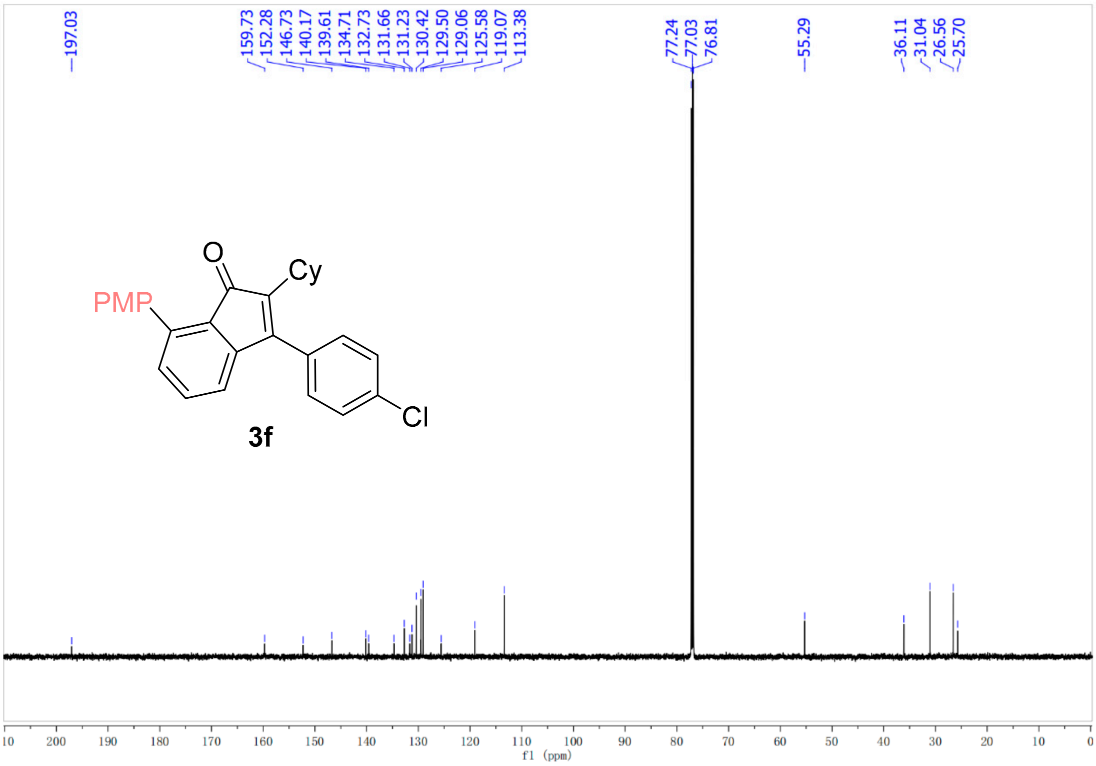

^{1}H NMR of **3g** in $CDCl_3$ (600 MHz, $CDCl_3$)

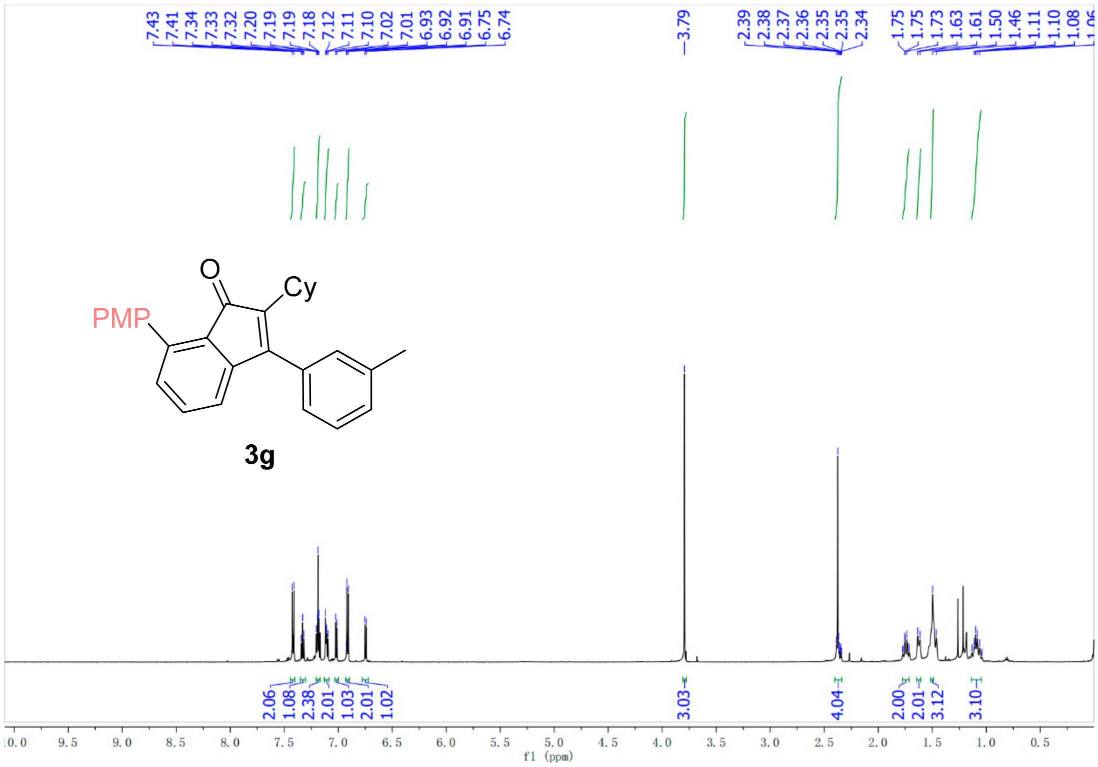

^{13}C NMR of **3g** in $CDCl_3$ (151 MHz, $CDCl_3$)

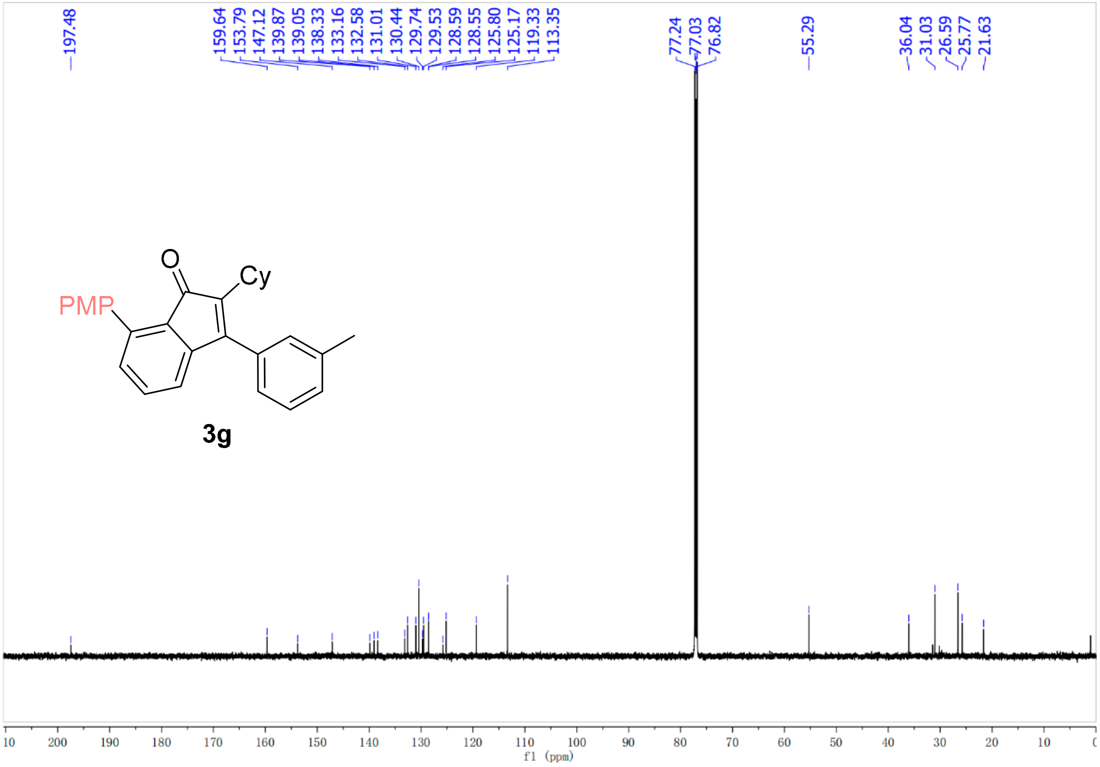

^{1}H NMR of **3h** in $CDCl_3$ (600 MHz, $CDCl_3$)

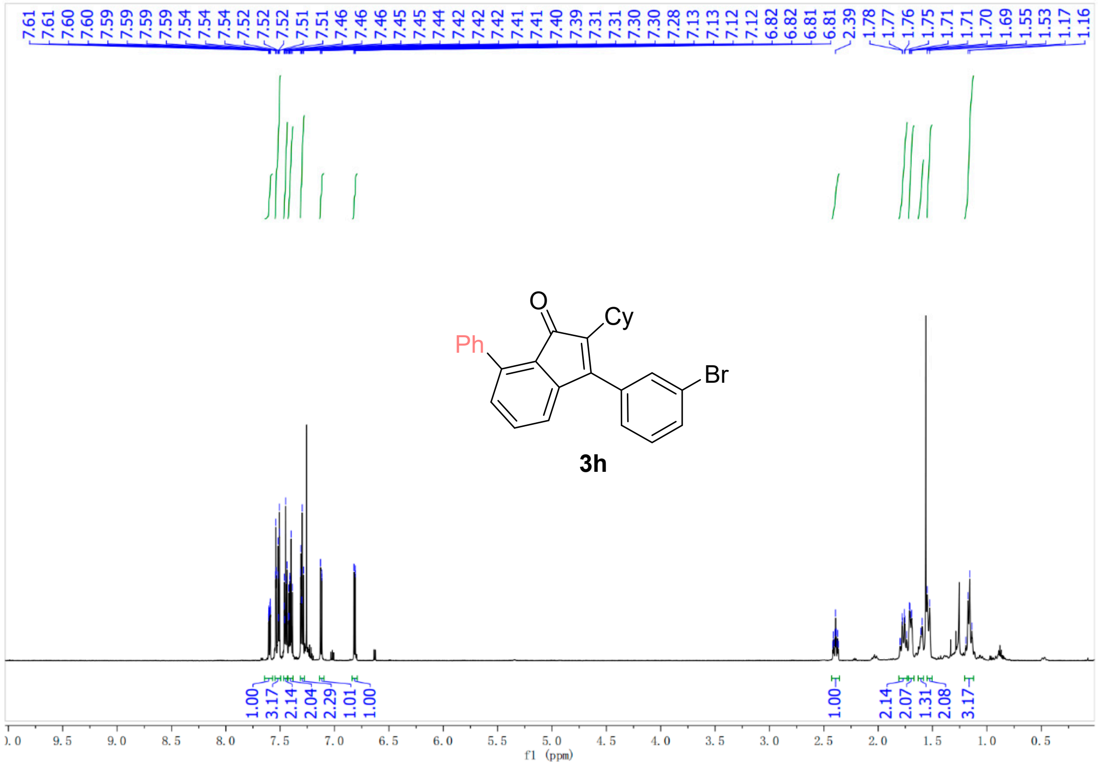

^{13}C NMR of **3h** in $CDCl_3$ (151 MHz, $CDCl_3$)

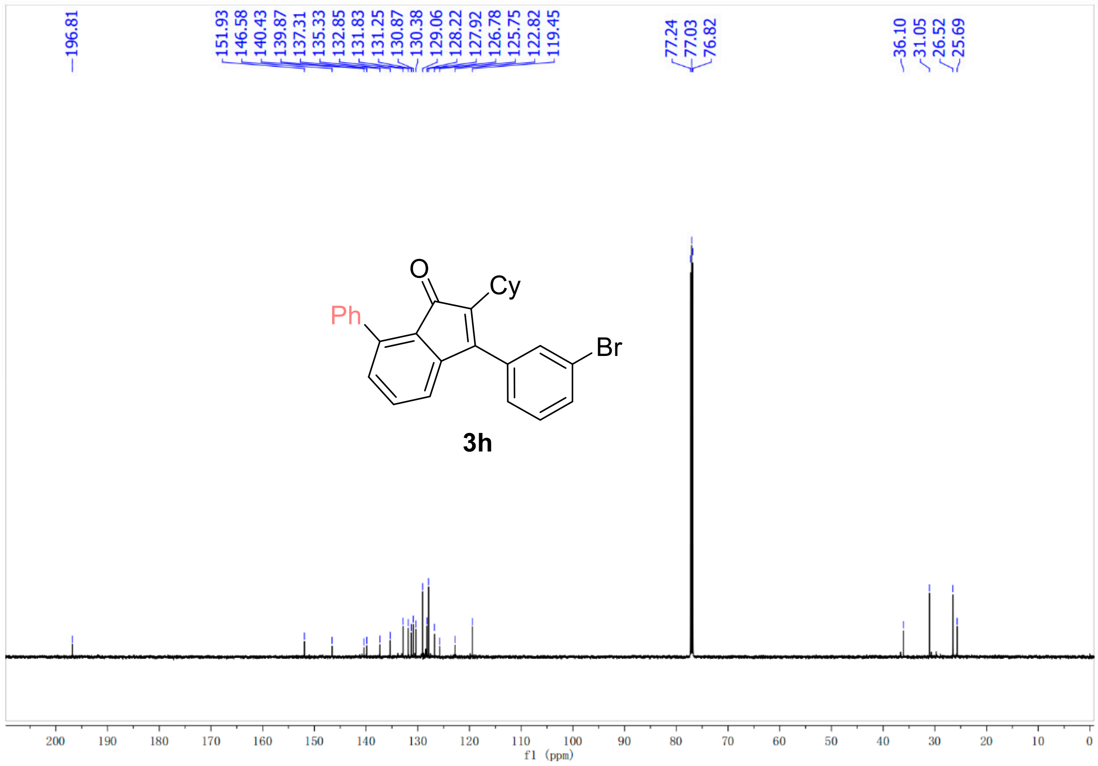

^{1}H NMR of **3i** in $CDCl_3$ (600 MHz, $CDCl_3$)

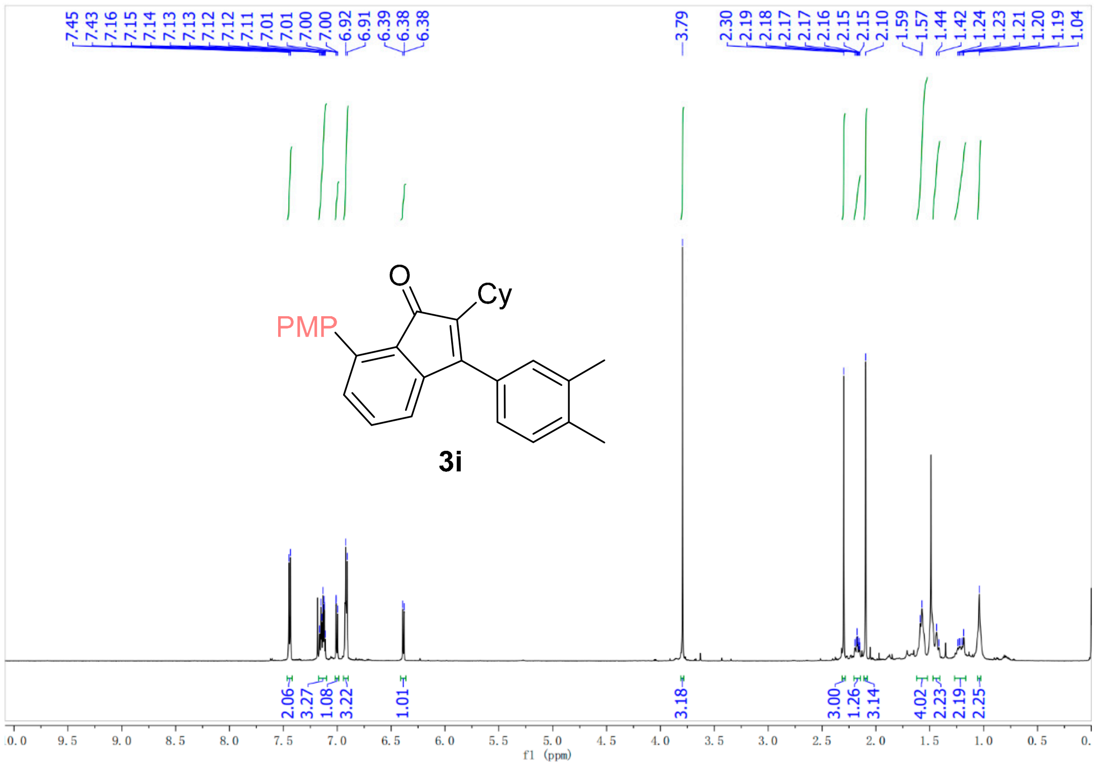

^{13}C NMR of **3i** in $CDCl_3$ (151 MHz, $CDCl_3$)

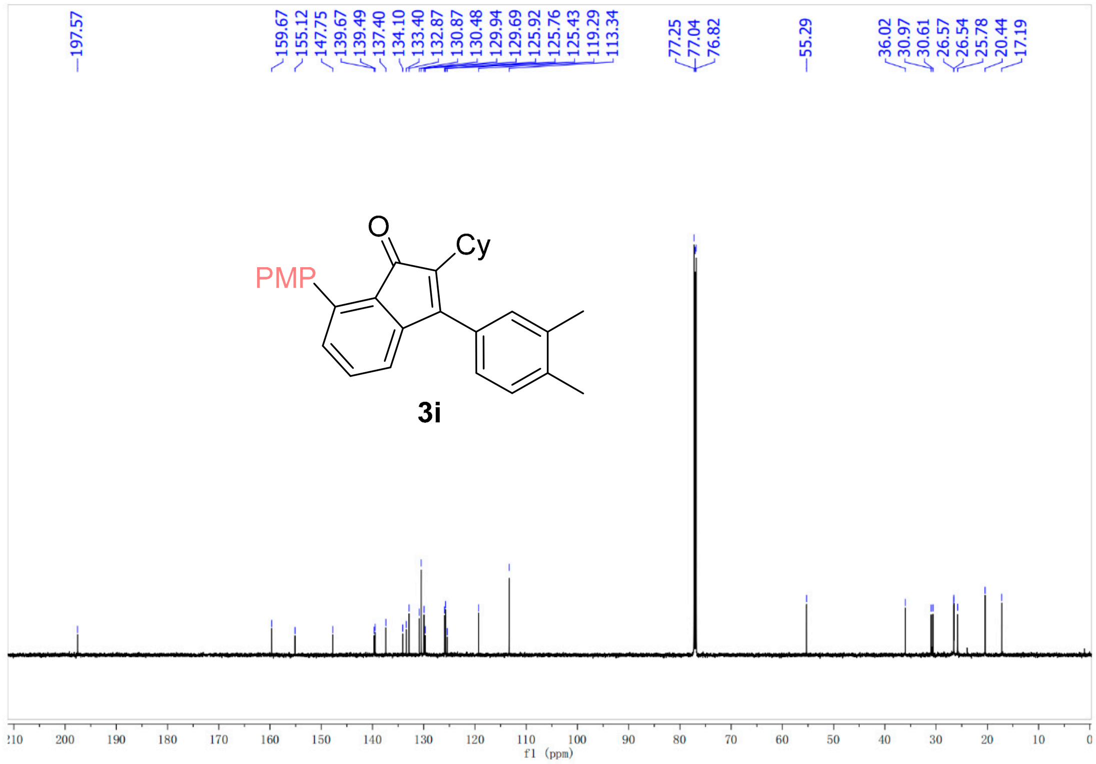

^{1}H NMR of **3j** in $CDCl_3$ (600 MHz, $CDCl_3$)

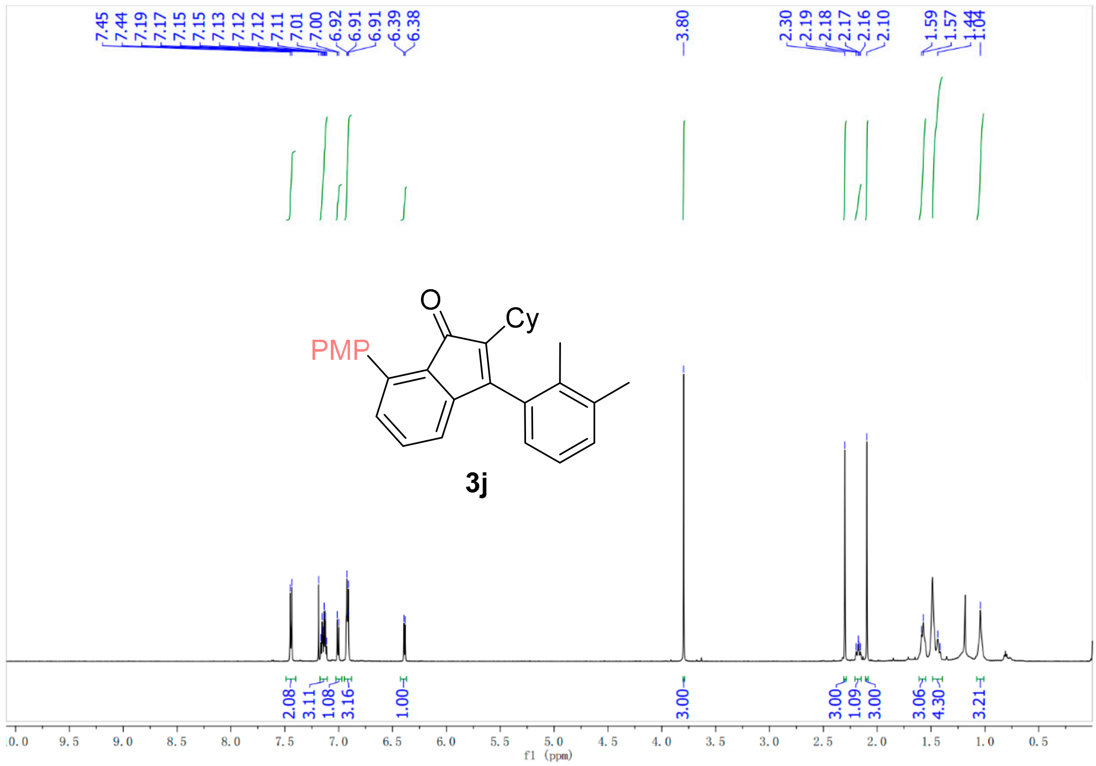

^{13}C NMR of **3j** in $CDCl_3$ (151 MHz, $CDCl_3$)

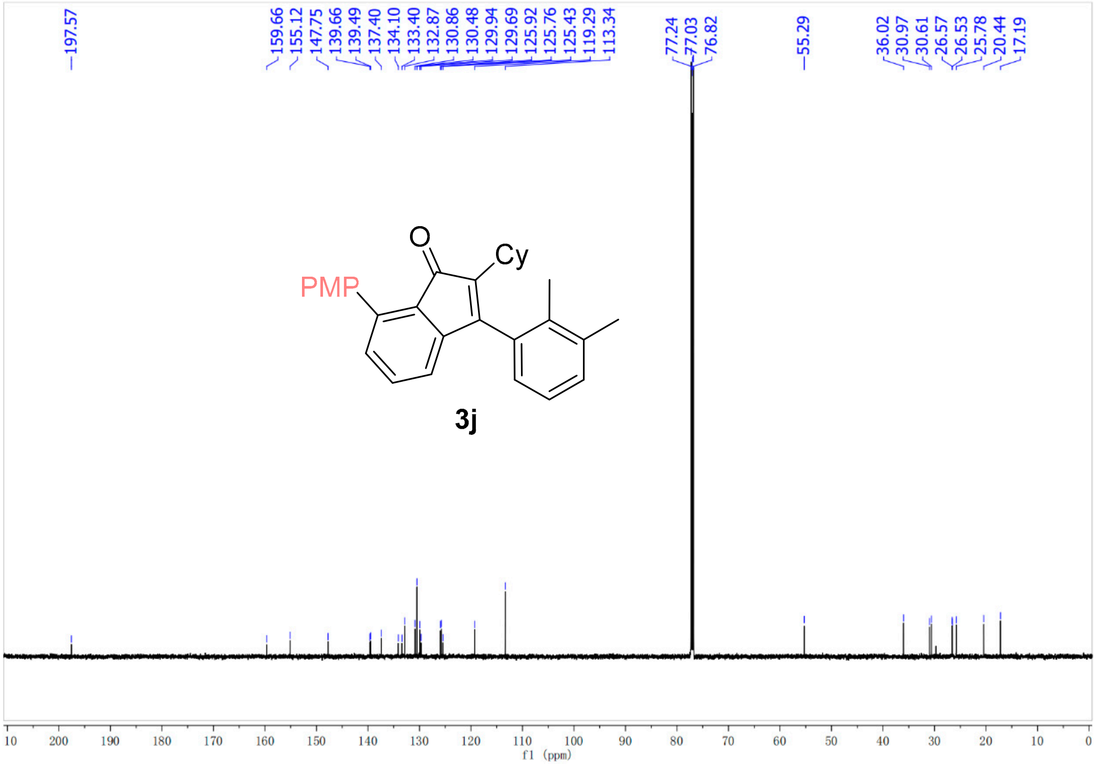

^1H NMR of **3k** in $CDCl_3$ (600 MHz, $CDCl_3$)

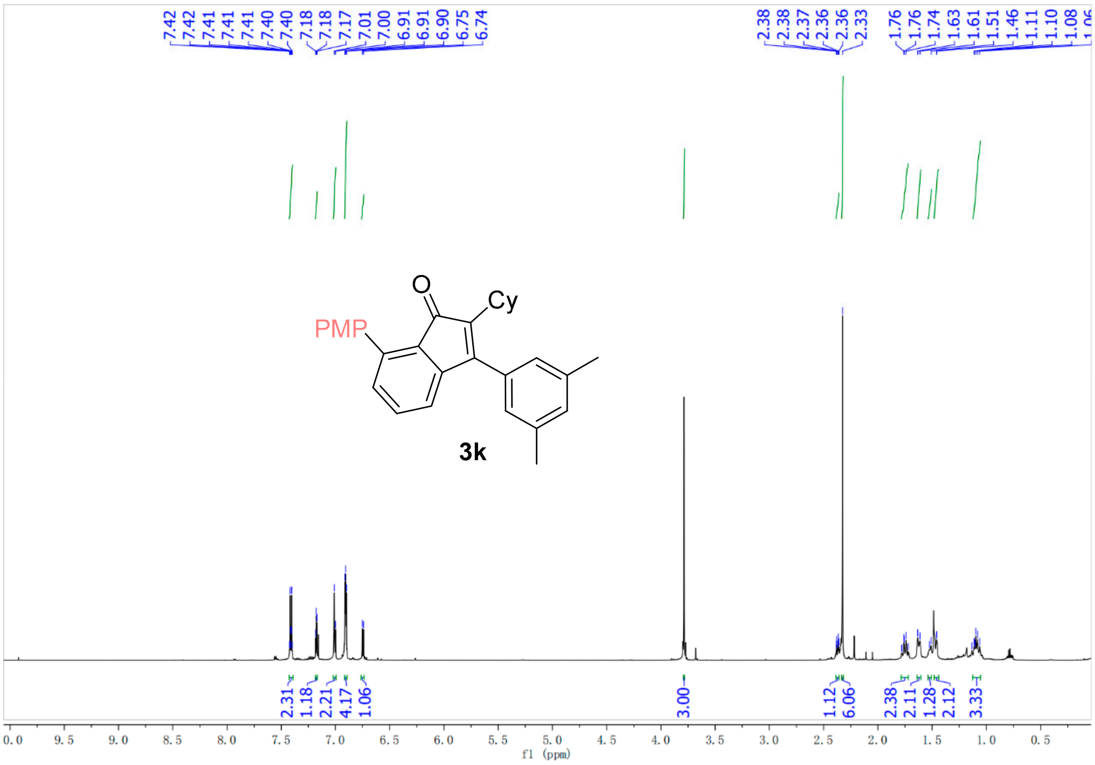

^{13}C NMR of **3k** in $CDCl_3$ (151 MHz, $CDCl_3$)

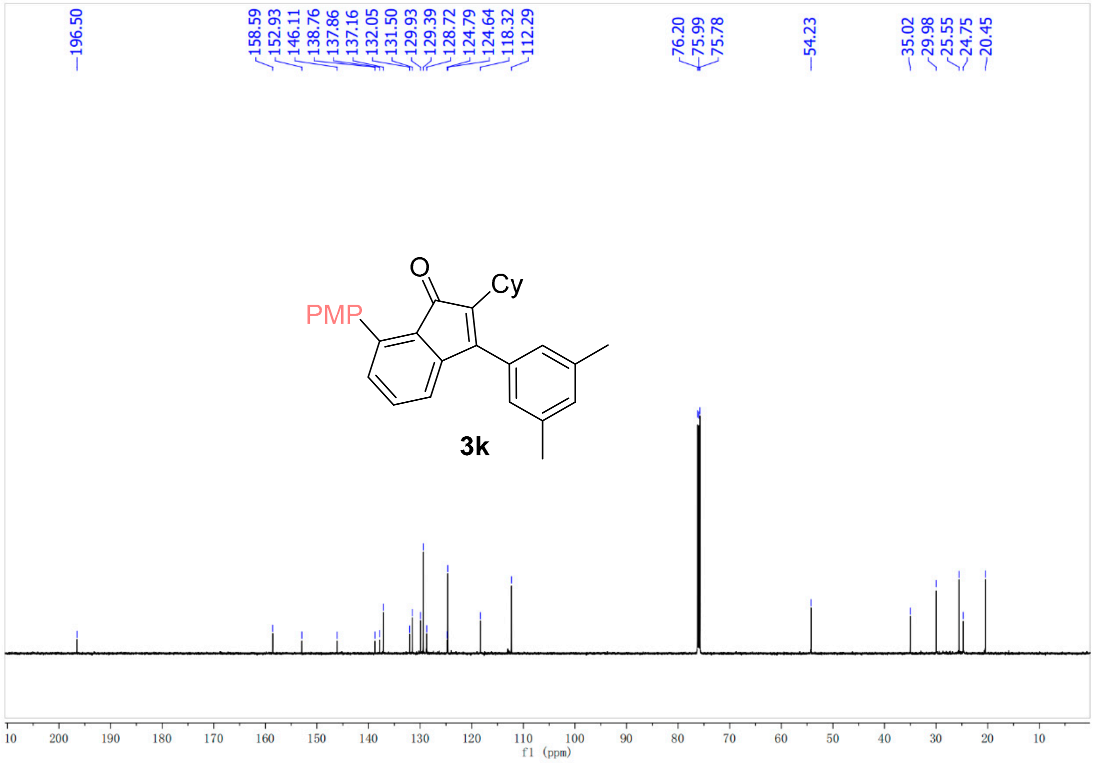

^{1}H NMR of **3l** in $CDCl_3$ (600 MHz, $CDCl_3$)

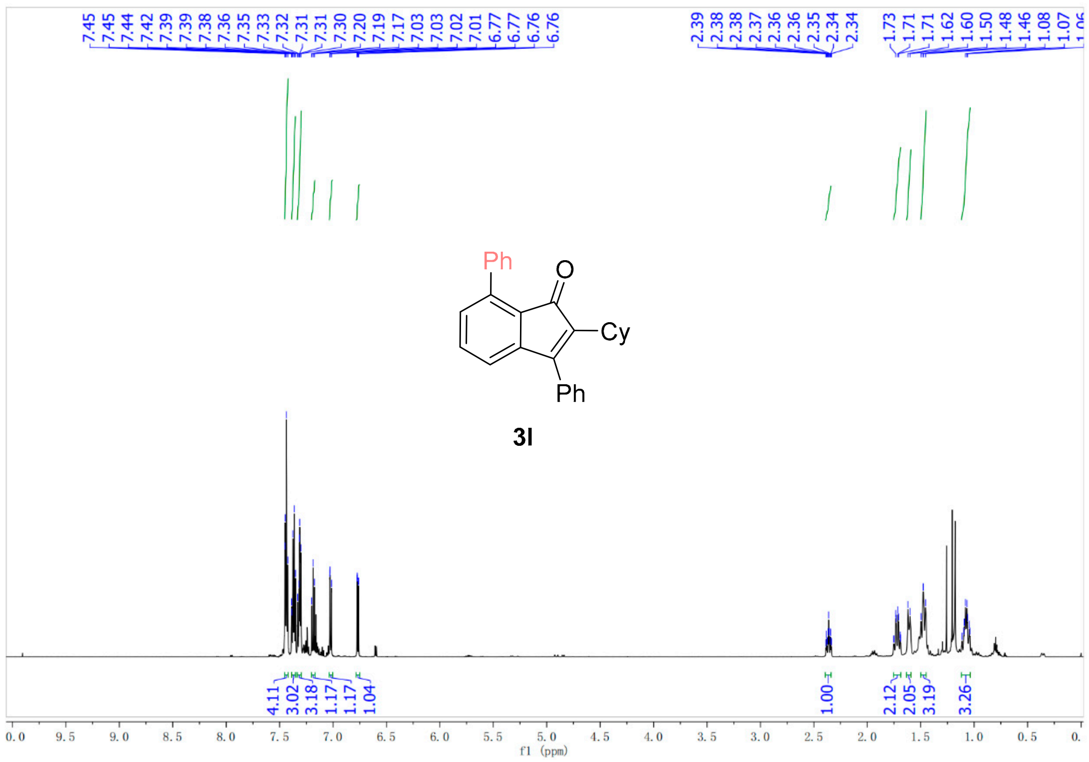

^{13}C NMR of **3l** in $CDCl_3$ (151 MHz, $CDCl_3$)

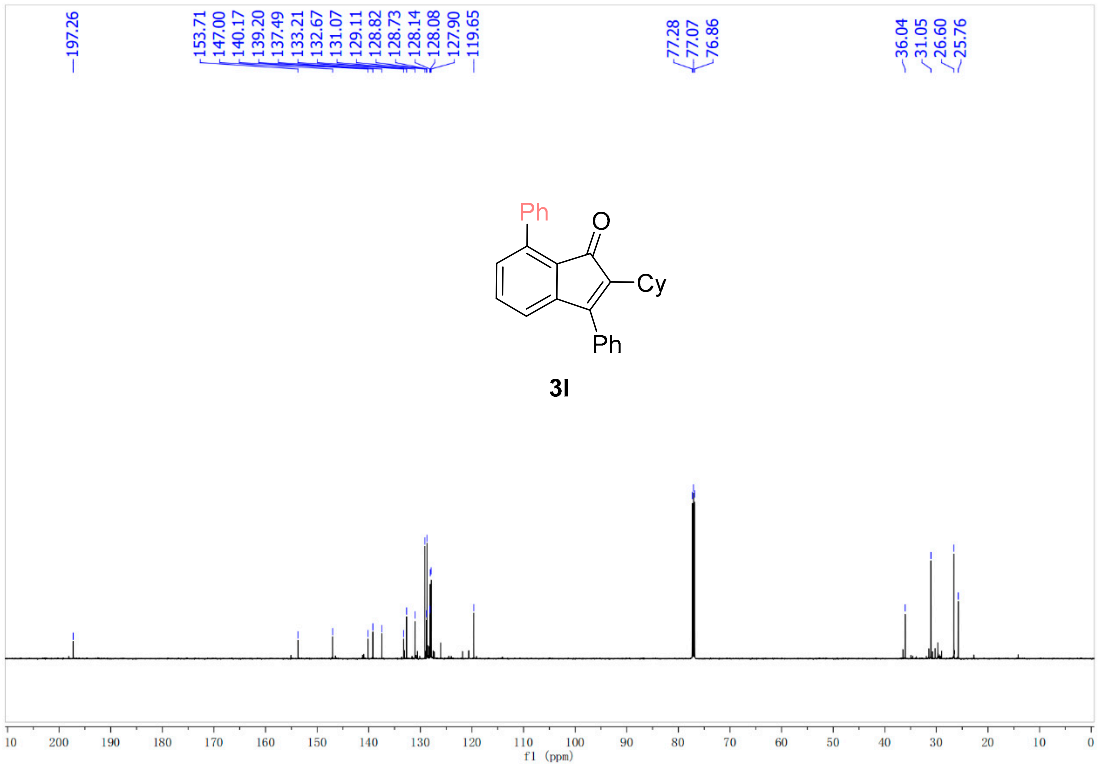

^{1}H NMR of **3m** in $CDCl_3$ (600 MHz, $CDCl_3$)

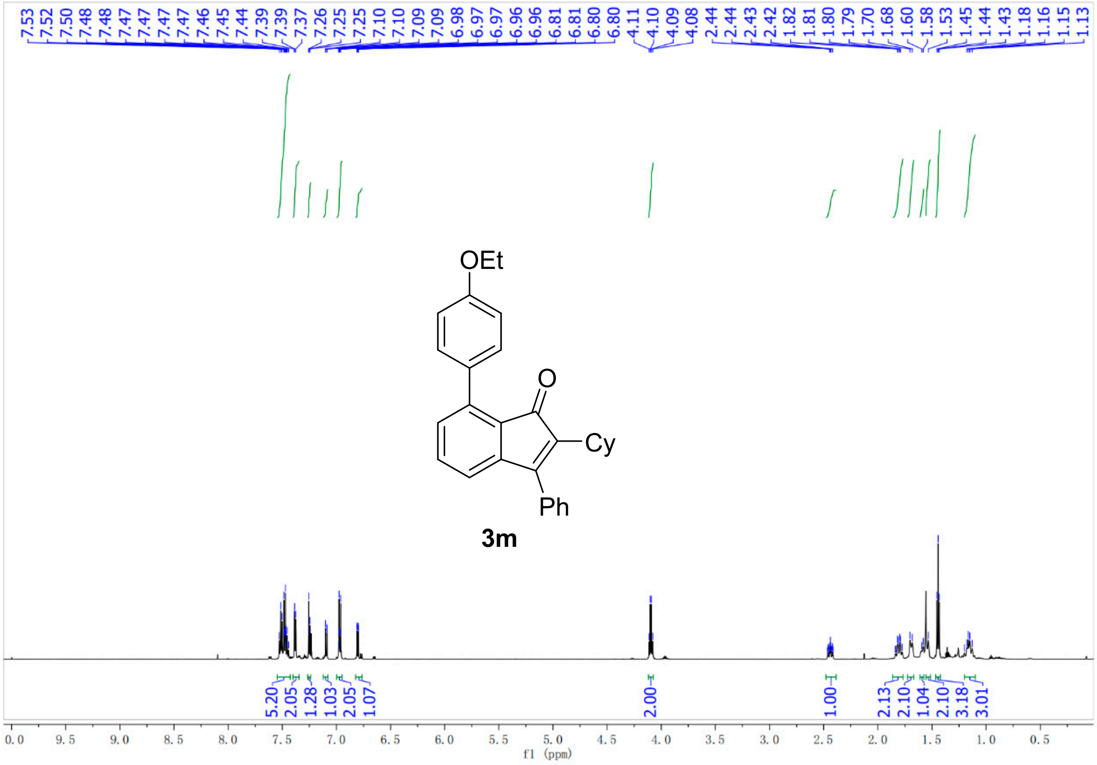

^{13}C NMR of **3m** in $CDCl_3$ (151 MHz, $CDCl_3$)

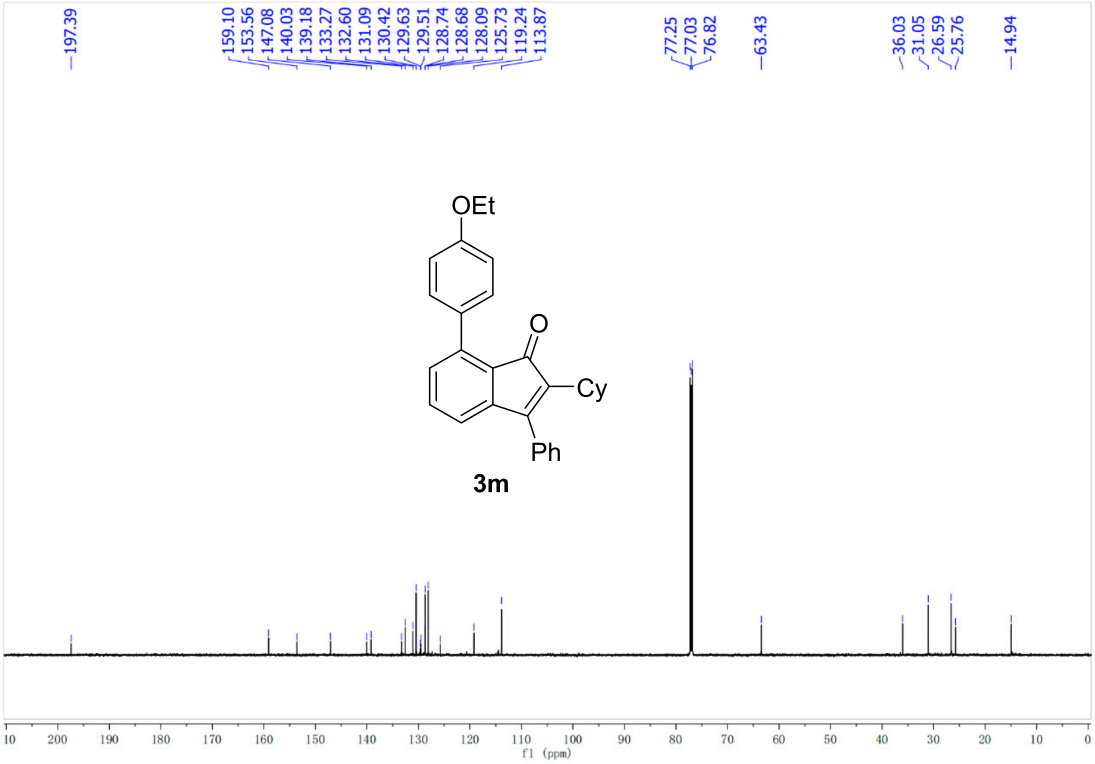

^{1}H NMR of **3n** in $CDCl_3$ (600 MHz, $CDCl_3$)

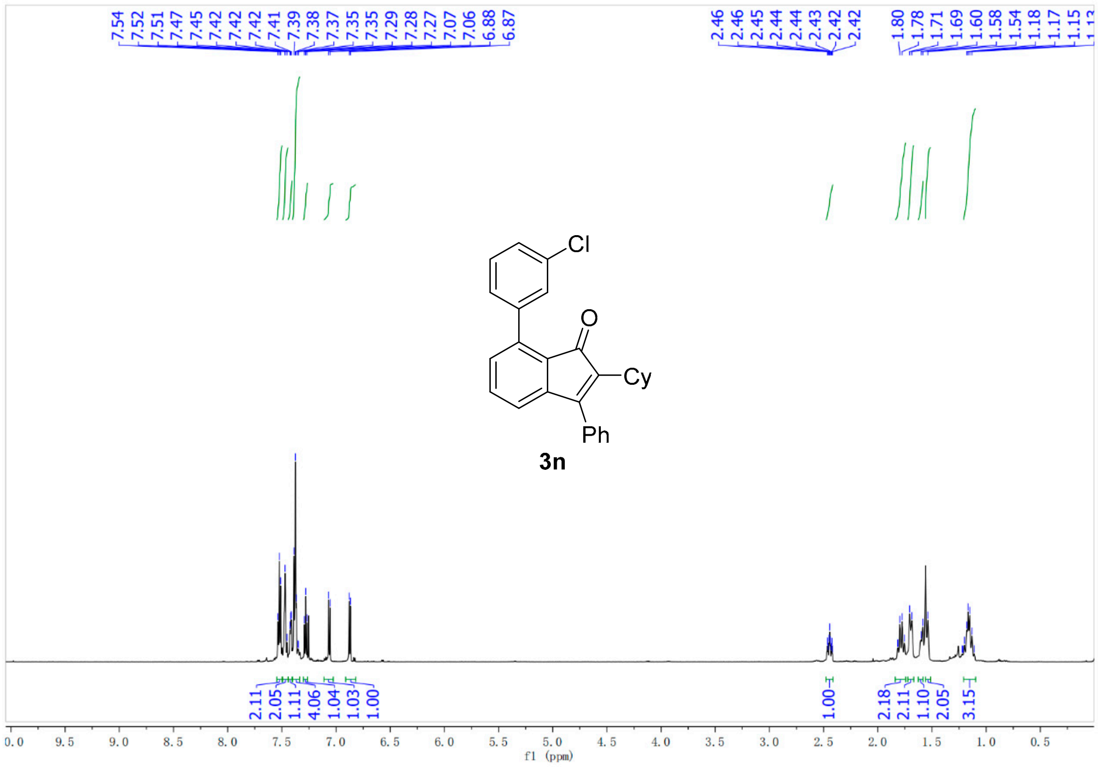

^{13}C NMR of **3n** in $CDCl_3$ (151 MHz, $CDCl_3$)

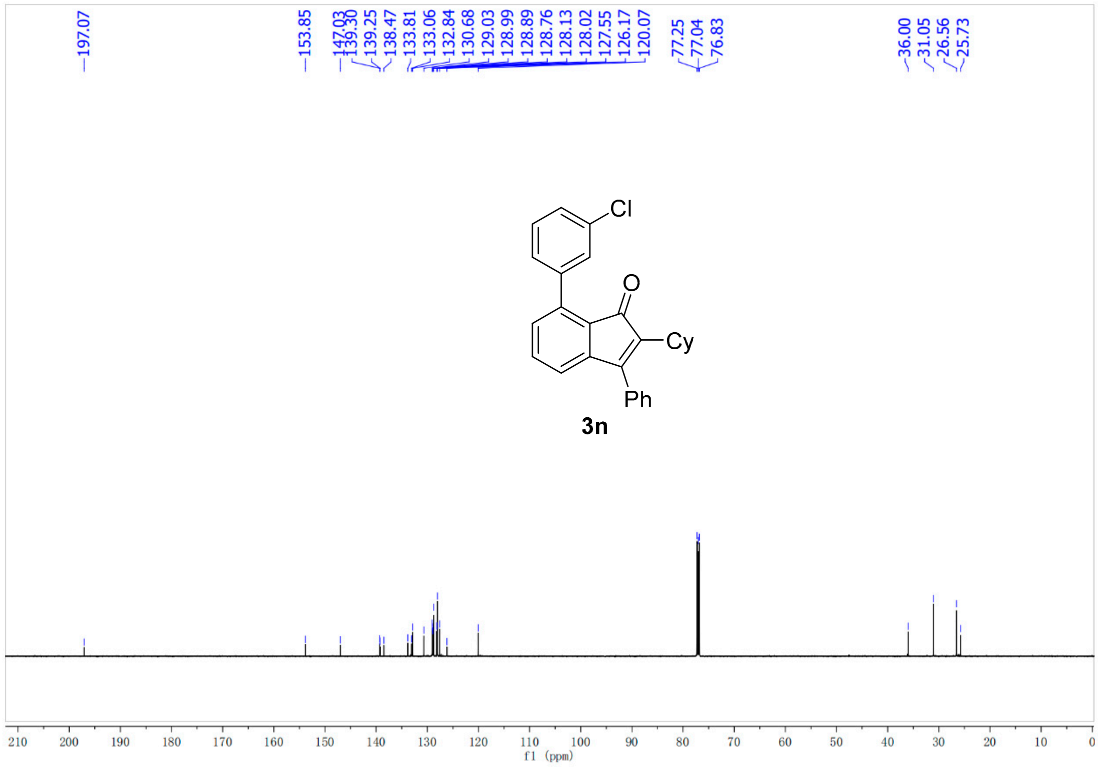

^{1}H NMR of **3o** in $CDCl_3$ (600 MHz, $CDCl_3$)

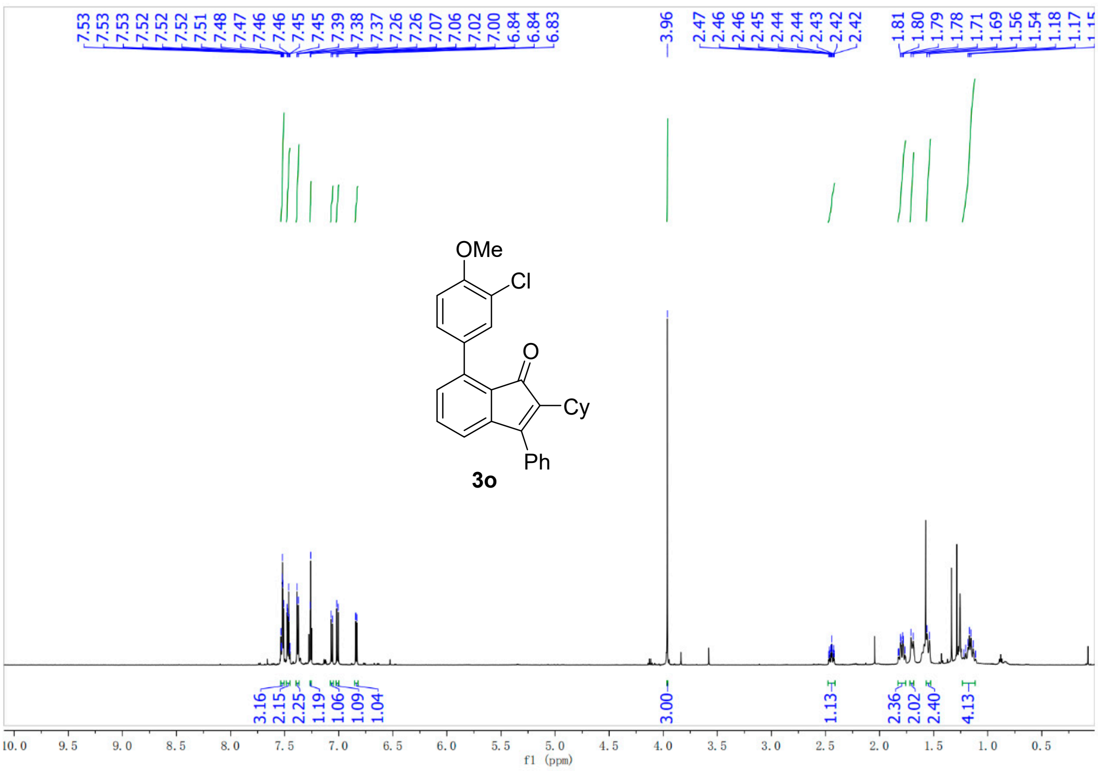

^{13}C NMR of **3o** in $CDCl_3$ (151 MHz, $CDCl_3$)

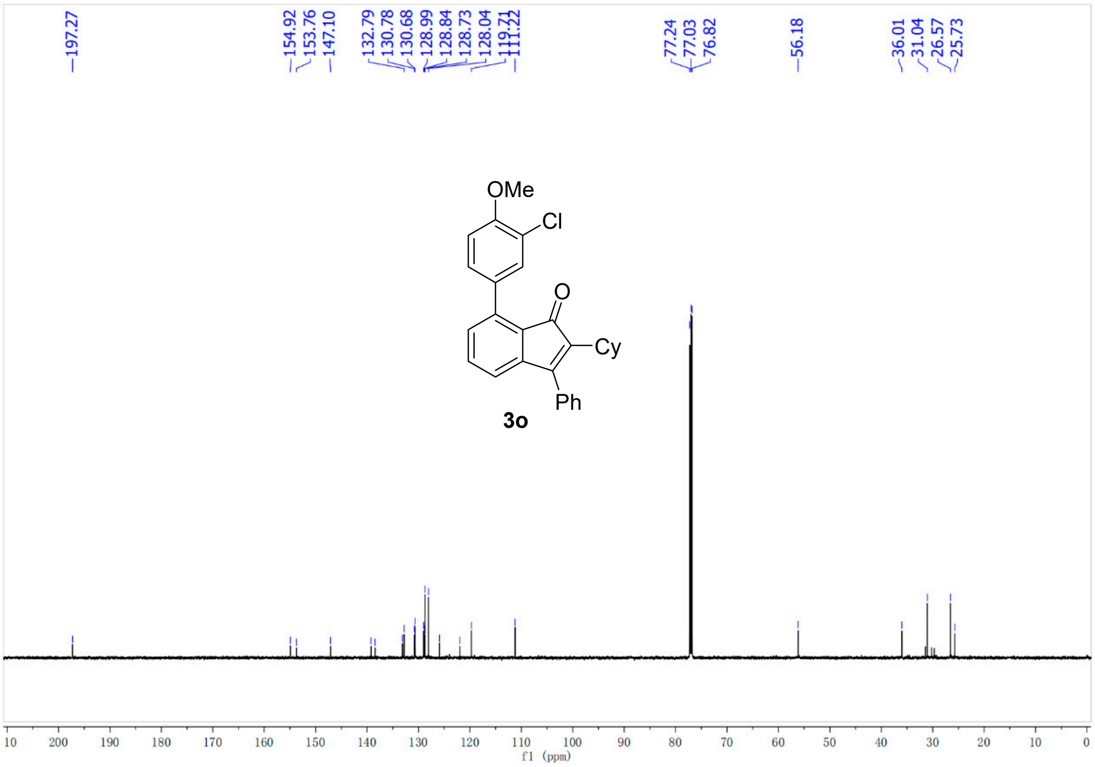

^{1}H NMR of **3p** in $CDCl_3$ (600 MHz, $CDCl_3$)

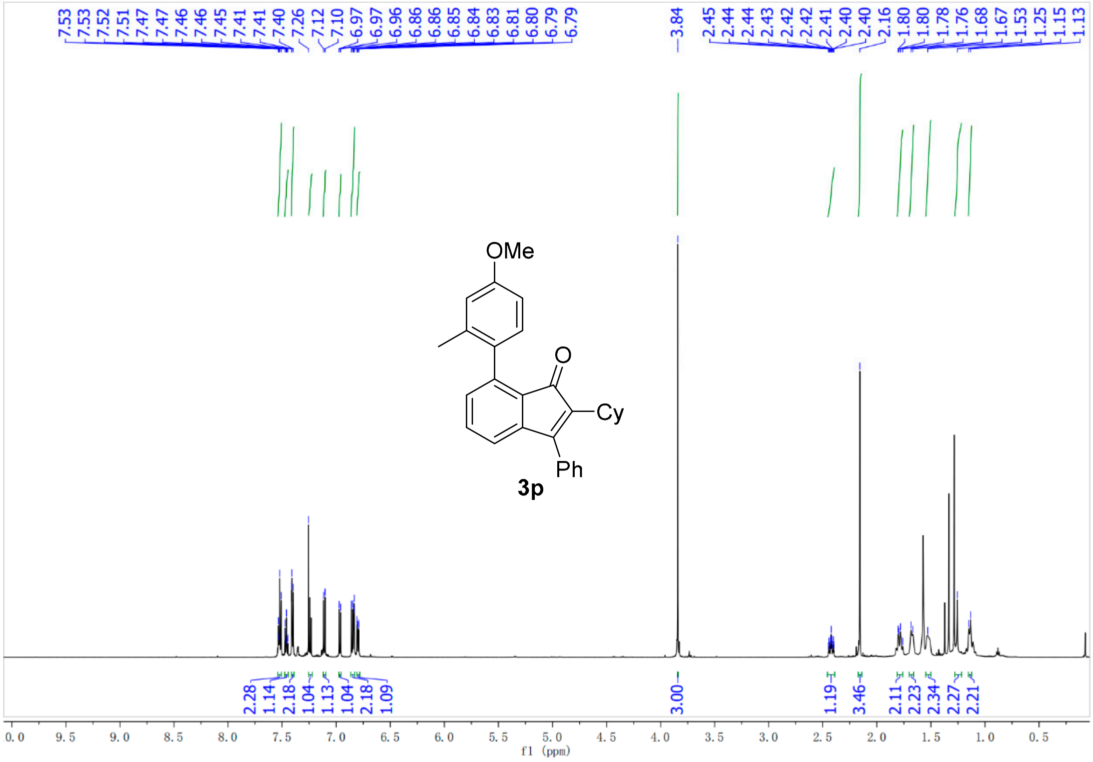

^{13}C NMR of **3p** in $CDCl_3$ (151 MHz, $CDCl_3$)

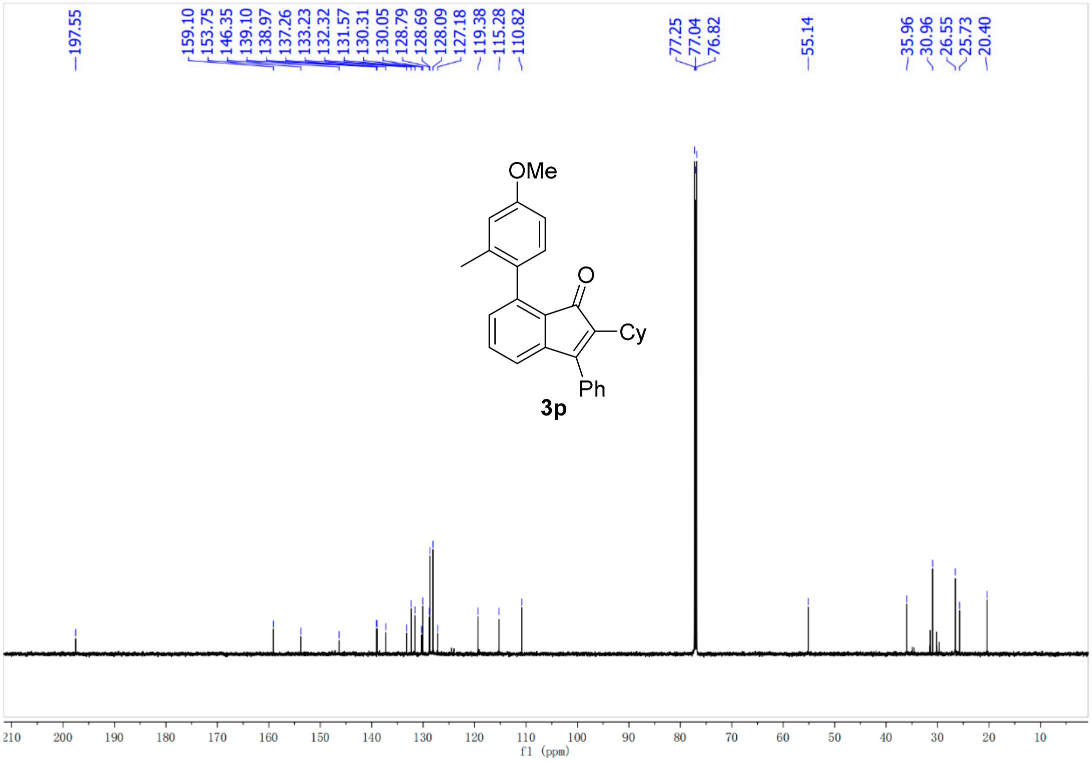

^{1}H NMR of **3q** in $CDCl_3$ (600 MHz, $CDCl_3$)

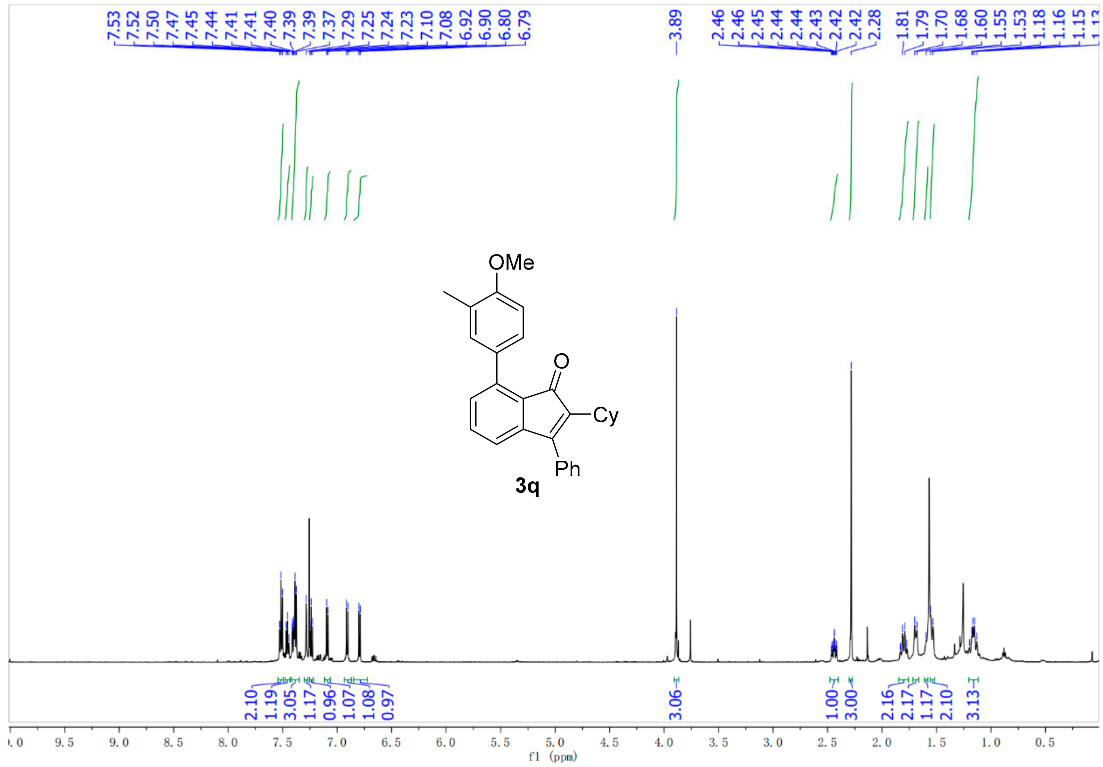

^{13}C NMR of **3q** in $CDCl_3$ (151 MHz, $CDCl_3$)

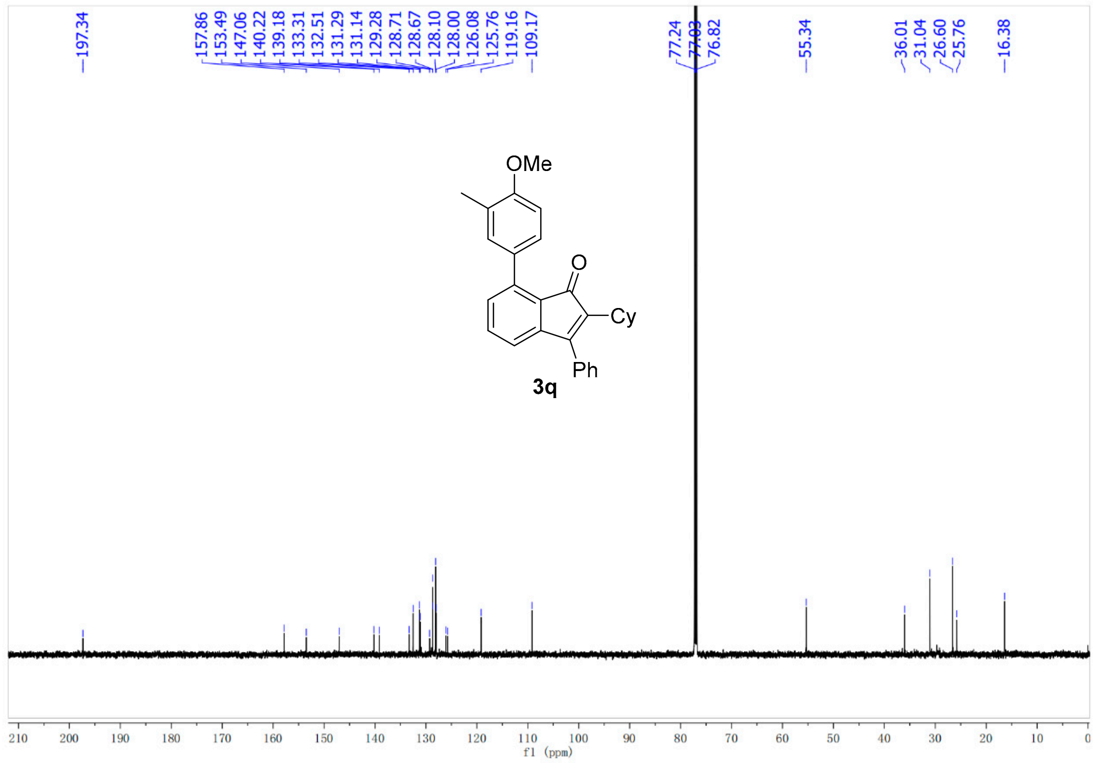

^1H NMR of **3r** in $CDCl_3$ (600 MHz, $CDCl_3$)

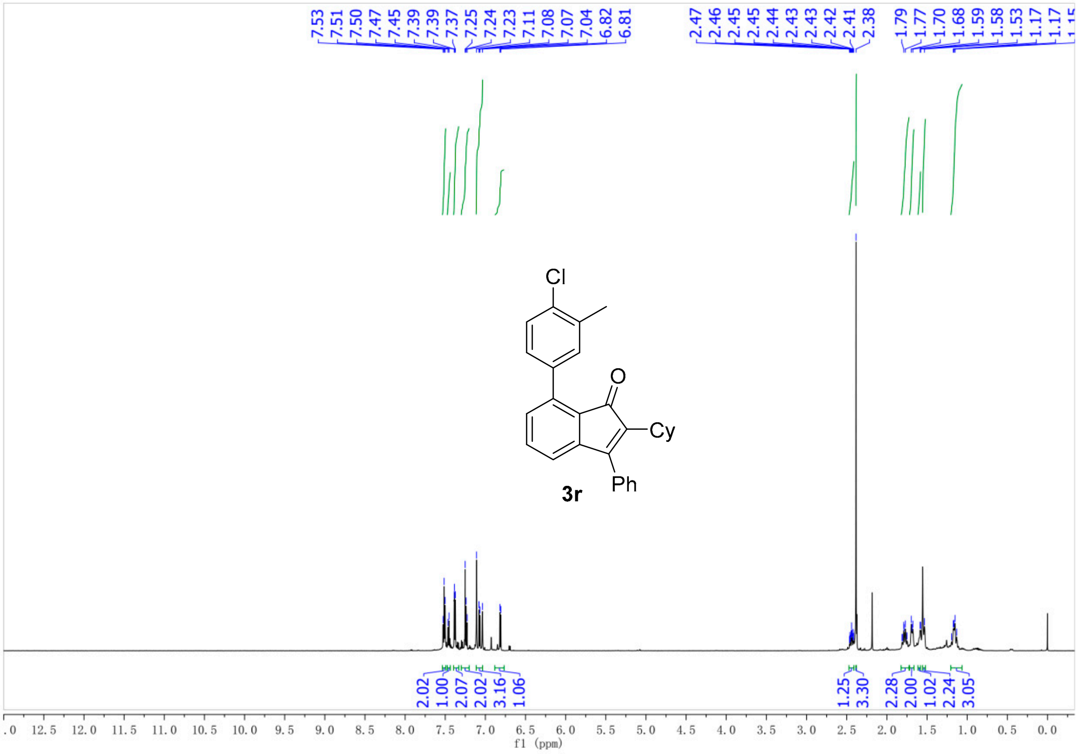

^{13}C NMR of **3r** in $CDCl_3$ (151 MHz, $CDCl_3$)

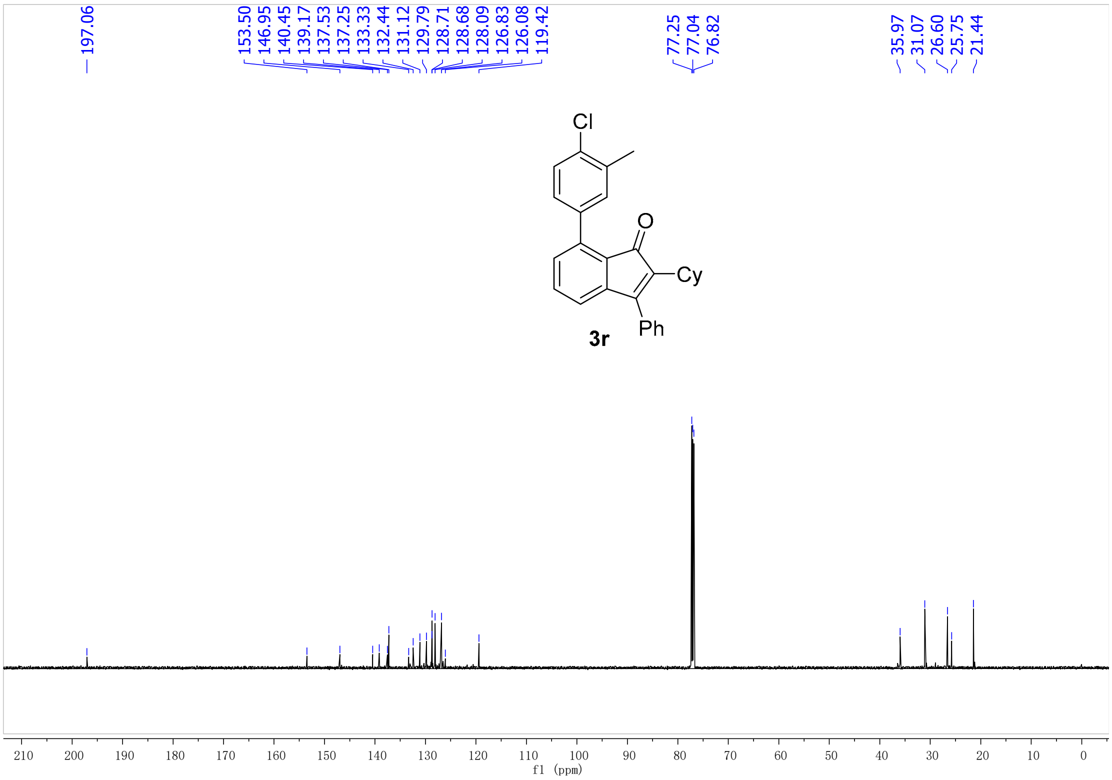

^{1}H NMR of **3s** in $CDCl_3$ (600 MHz, $CDCl_3$)

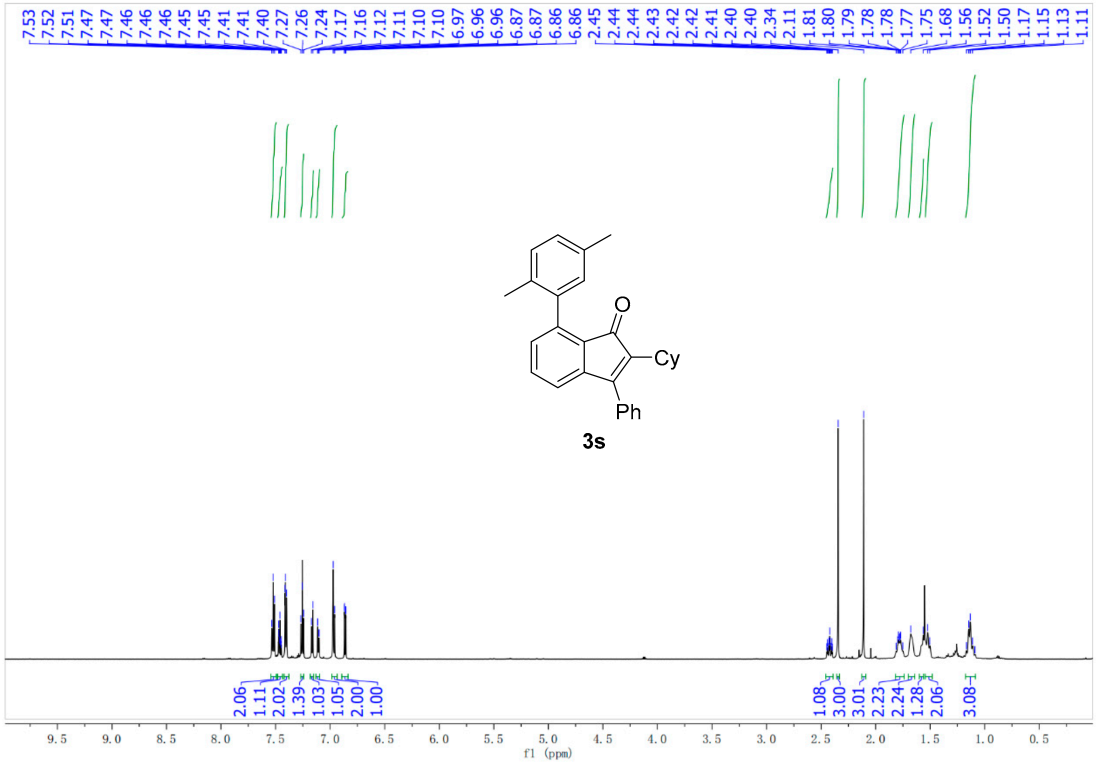

^{13}C NMR of **3s** in $CDCl_3$ (151 MHz, $CDCl_3$)

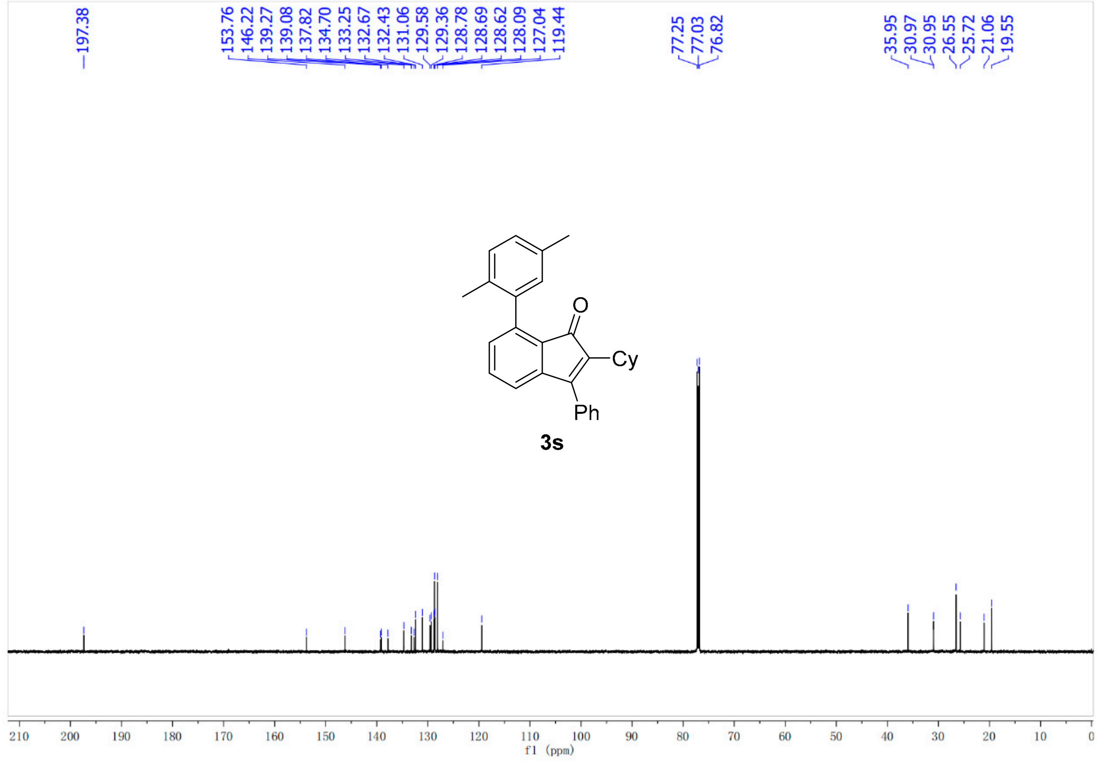

^{1}H NMR of **3t** in $CDCl_3$ (600 MHz, $CDCl_3$)

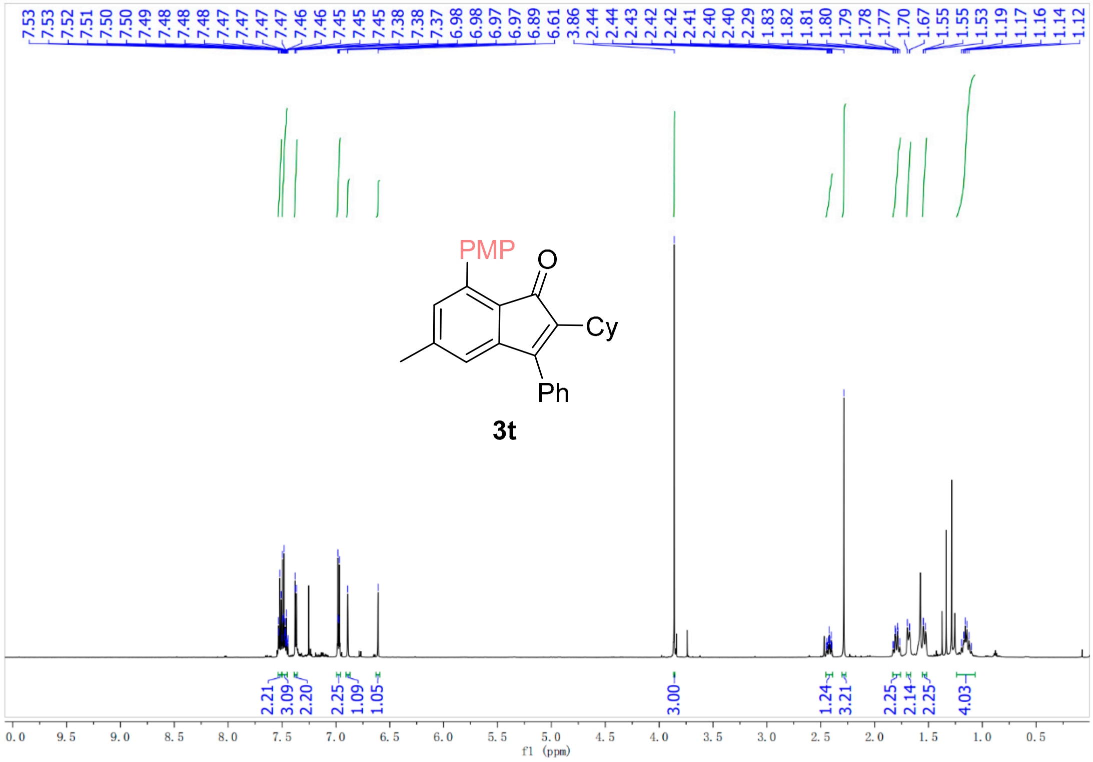

^{13}C NMR of **3t** in $CDCl_3$ (151 MHz, $CDCl_3$)

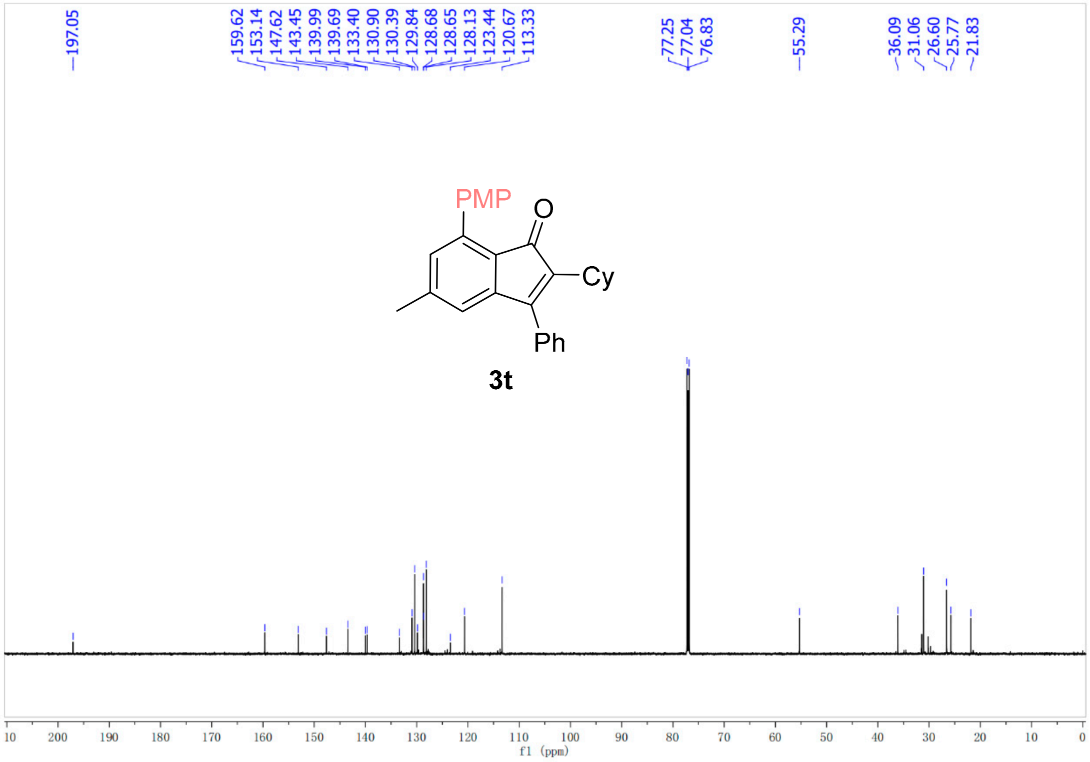

^{1}H NMR of **3u** in $CDCl_3$ (600 MHz, $CDCl_3$)

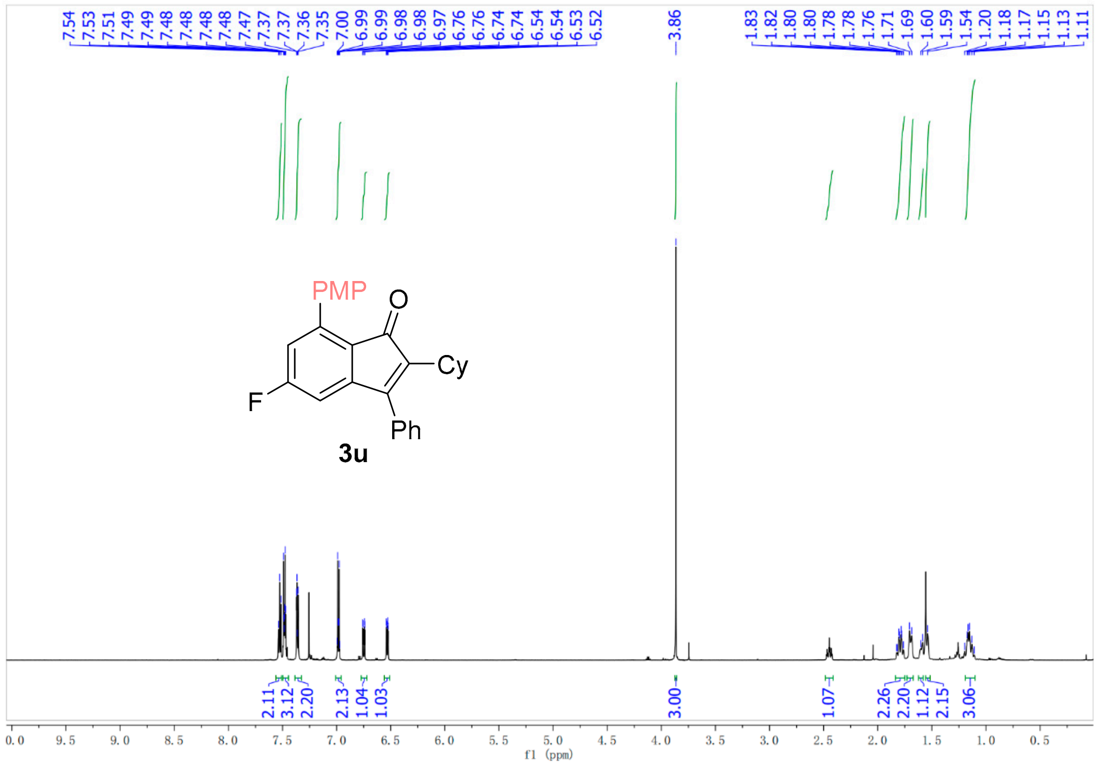

^{13}C NMR of **3u** in $CDCl_3$ (151 MHz, $CDCl_3$)

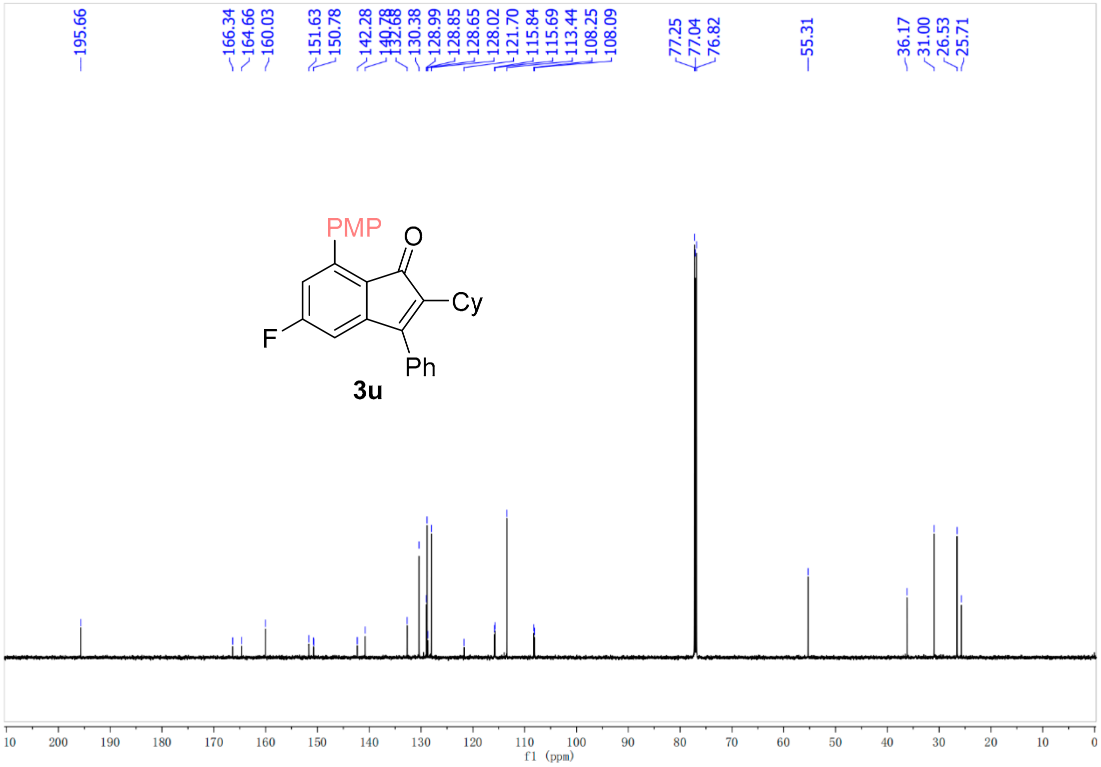

^{1}H NMR of **3v** in $CDCl_3$ (600 MHz, $CDCl_3$)

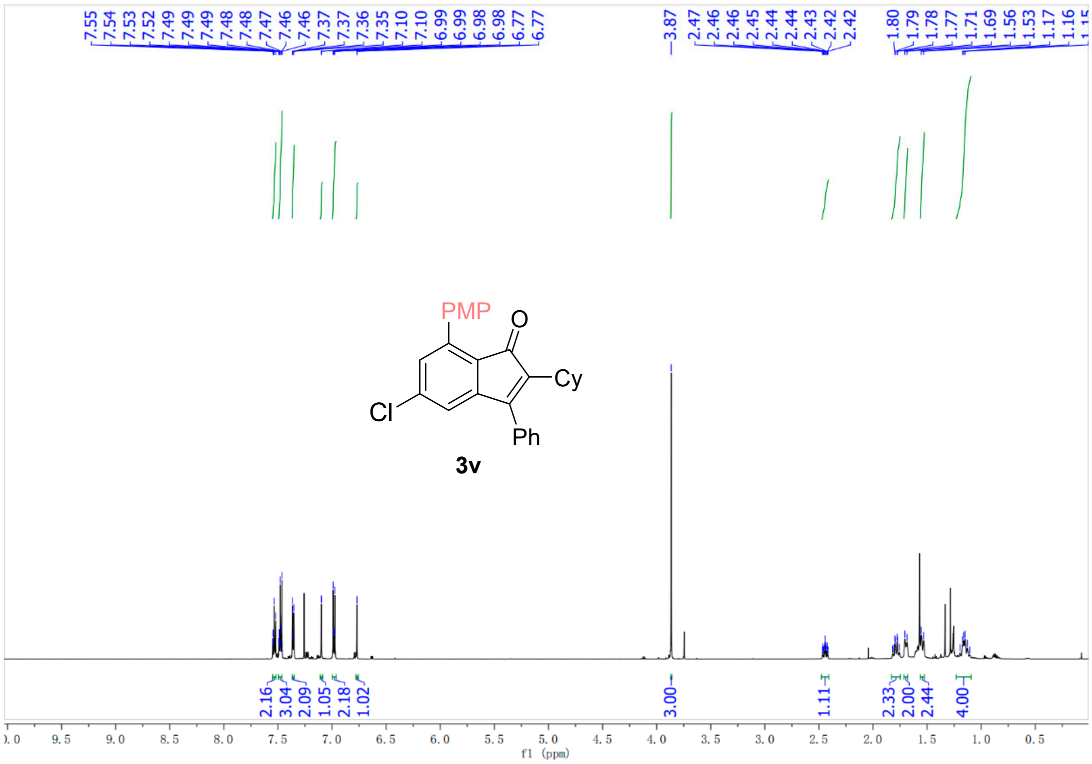

^{13}C NMR of **3v** in $CDCl_3$ (151 MHz, $CDCl_3$)

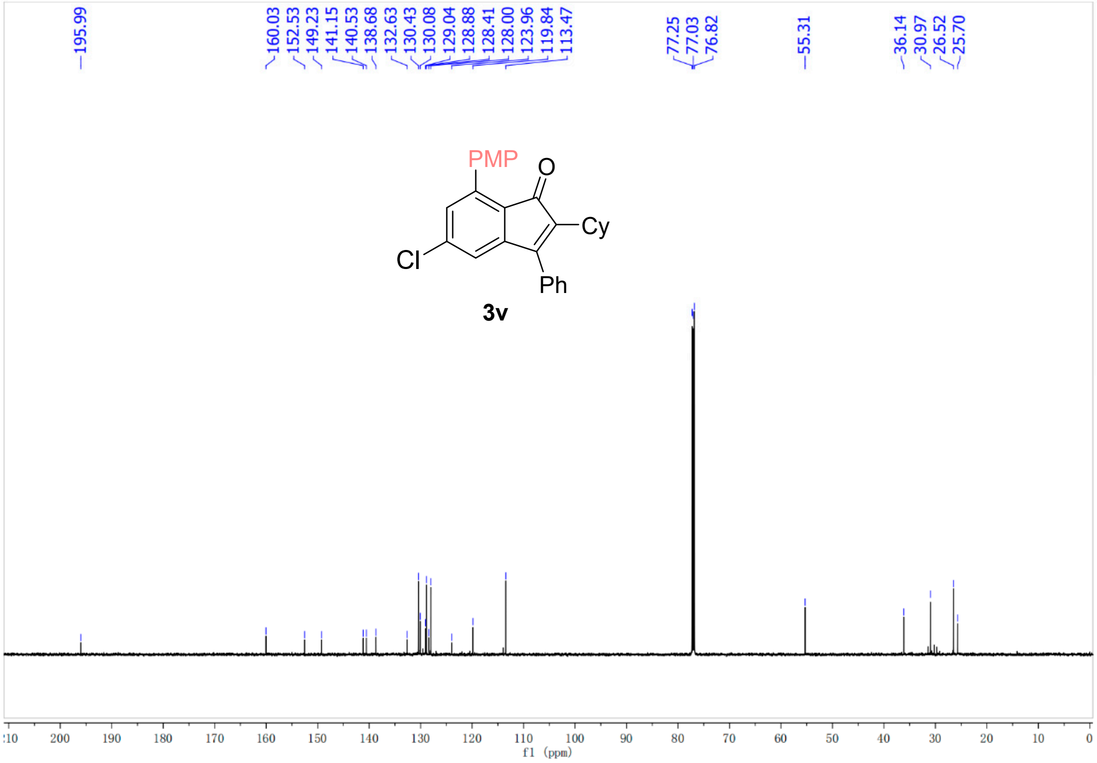

^1H NMR of **3w** in $CDCl_3$ (600 MHz, $CDCl_3$)

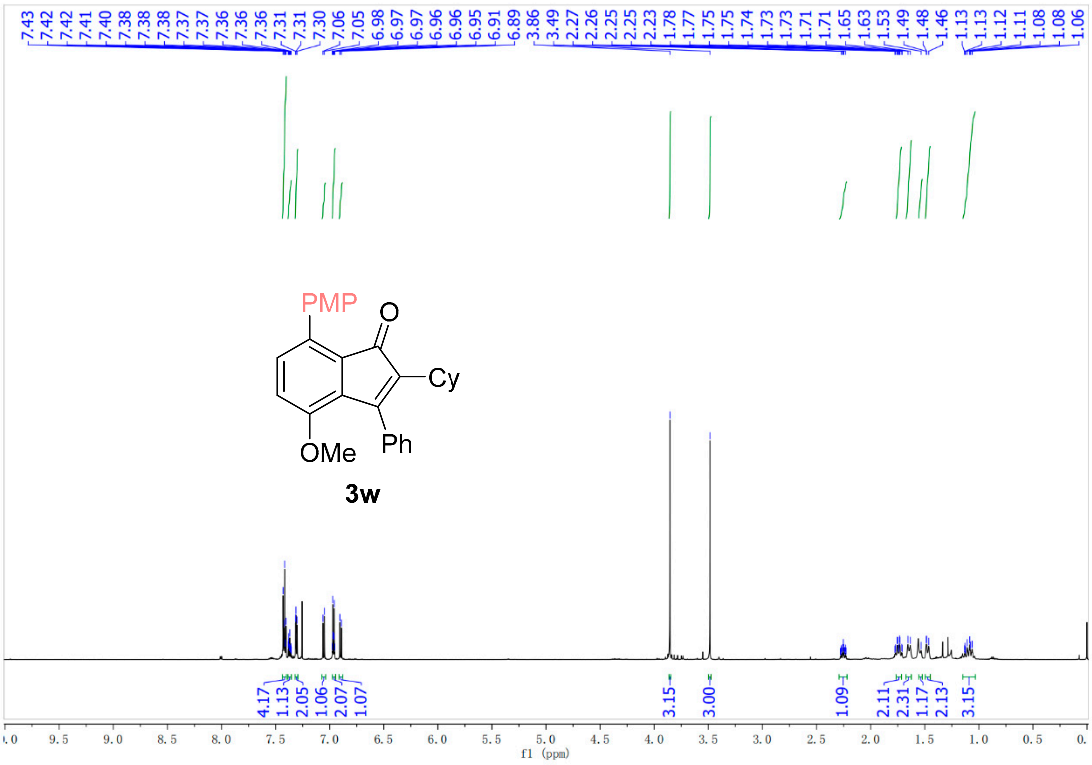

^{13}C NMR of **3w** in $CDCl_3$ (151 MHz, $CDCl_3$)

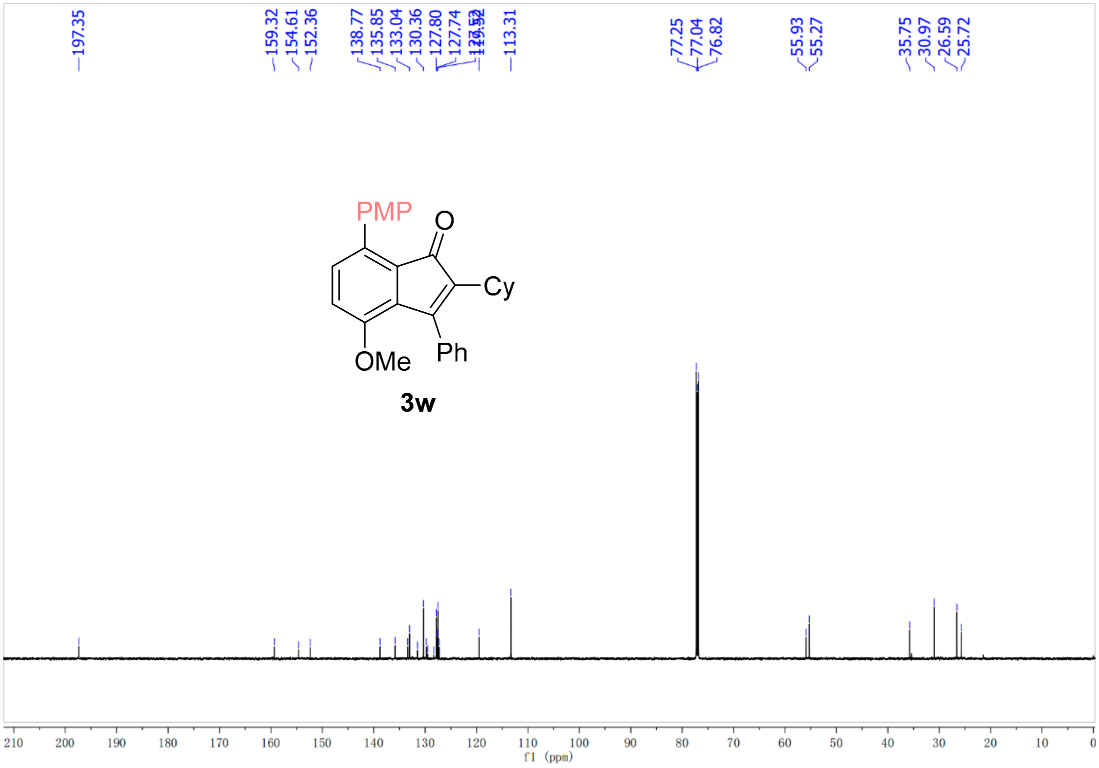

^{1}H NMR of **3x** in $CDCl_3$ (600 MHz, $CDCl_3$)

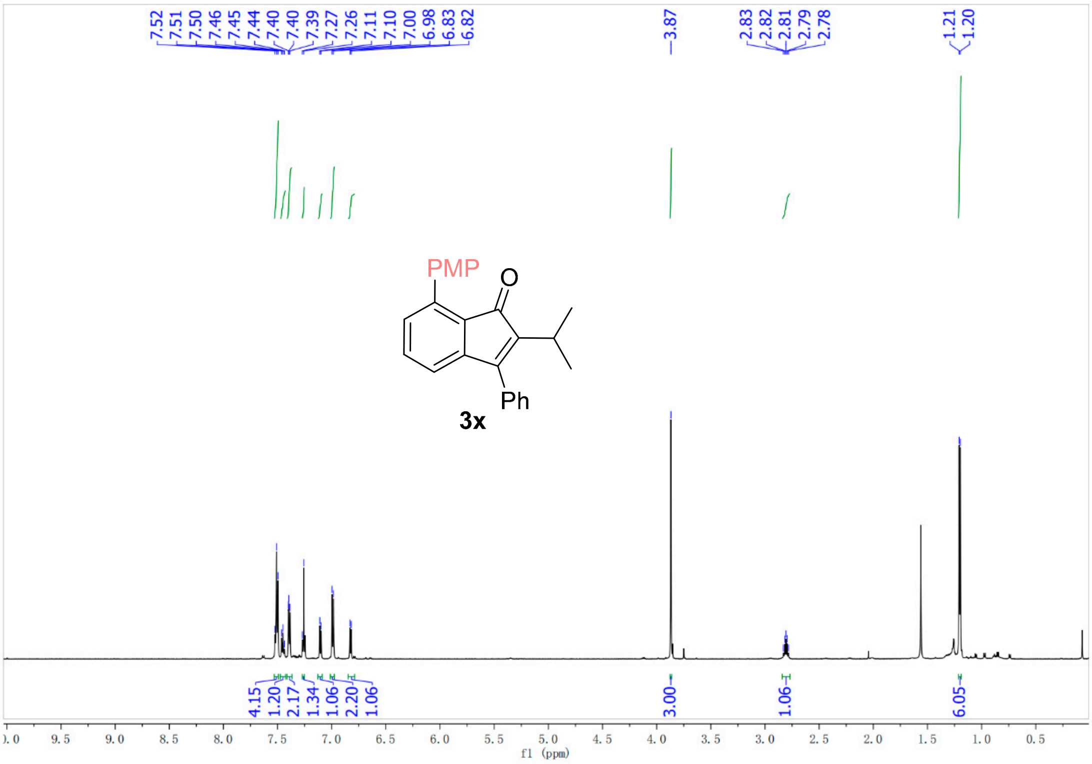

^{13}C NMR of **3x** in $CDCl_3$ (151 MHz, $CDCl_3$)

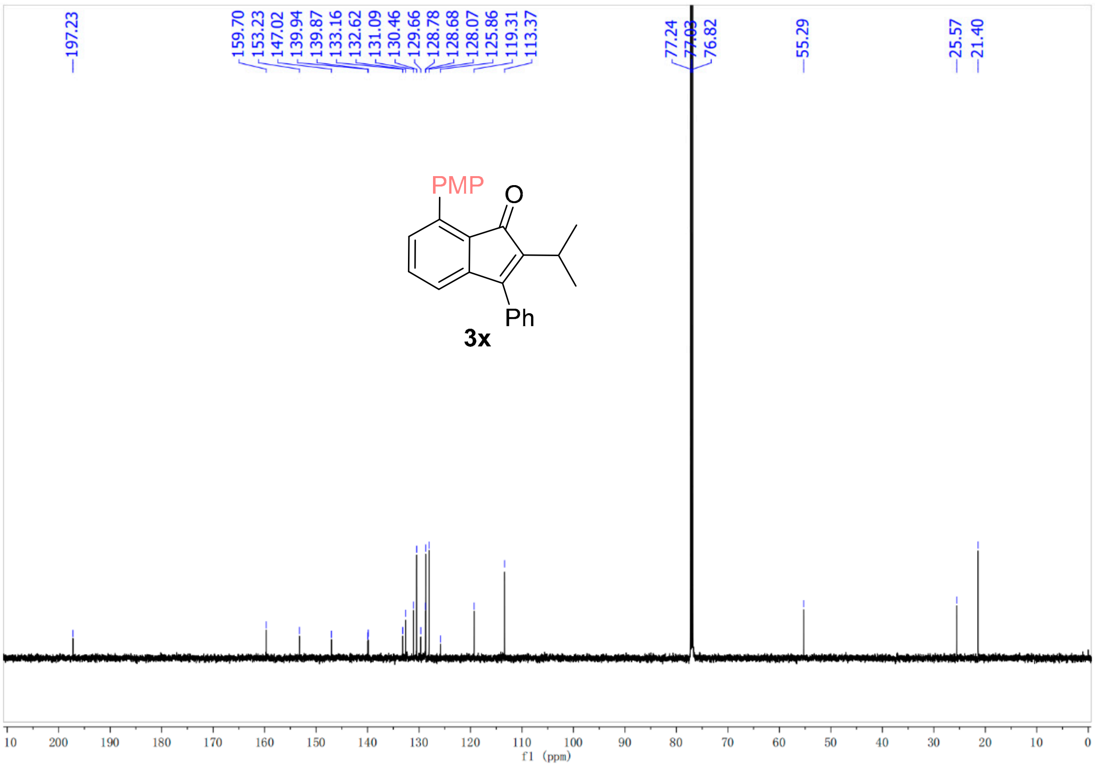

^1H NMR of **3y** in $CDCl_3$ (600 MHz, $CDCl_3$)

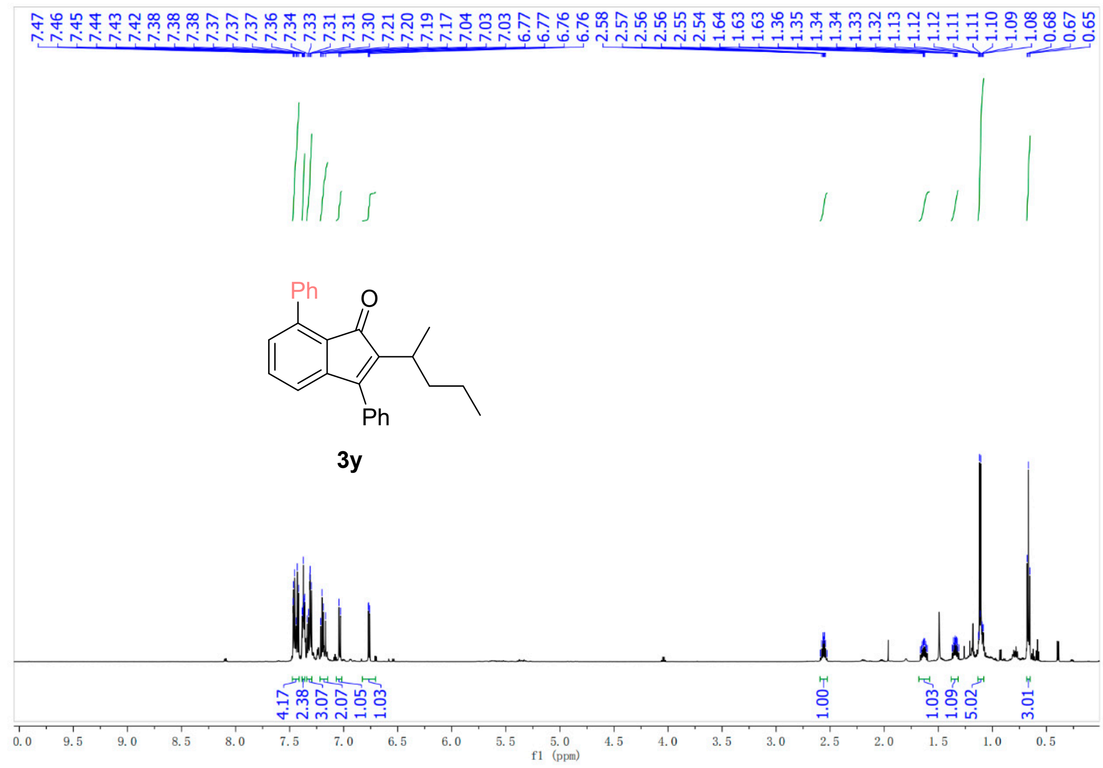

^{13}C NMR of **3y** in $CDCl_3$ (151 MHz, $CDCl_3$)

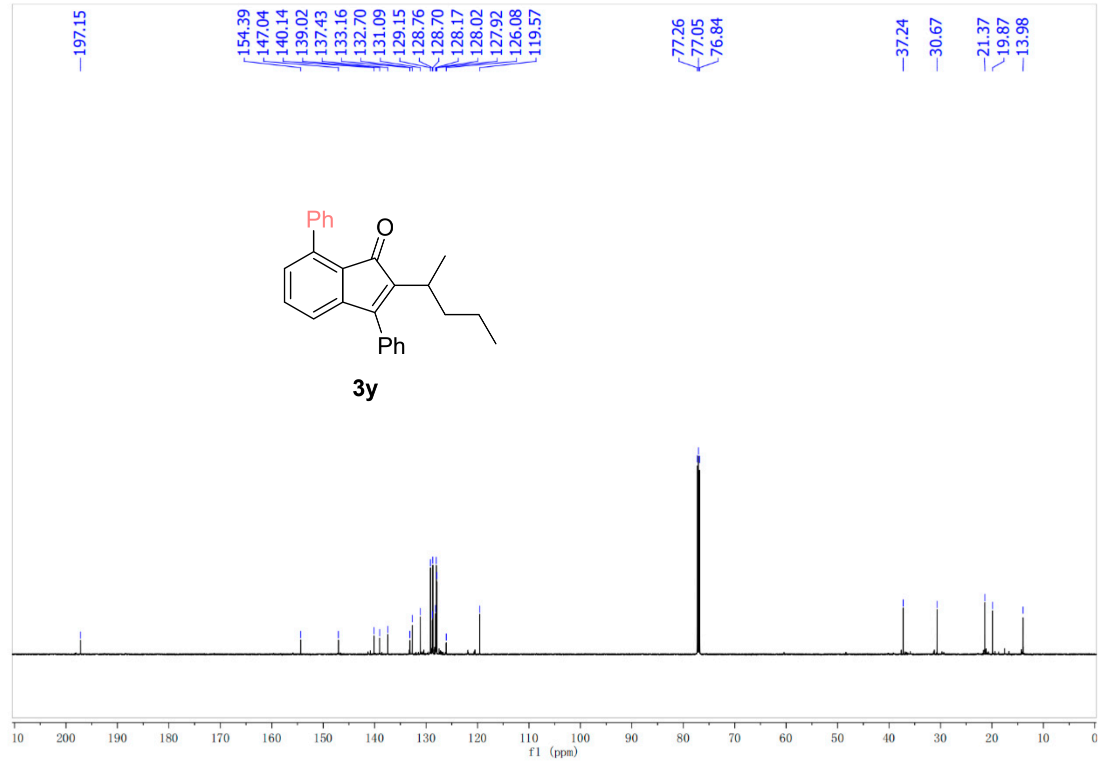

^{1}H NMR of **3z** in $CDCl_3$ (600 MHz, $CDCl_3$)

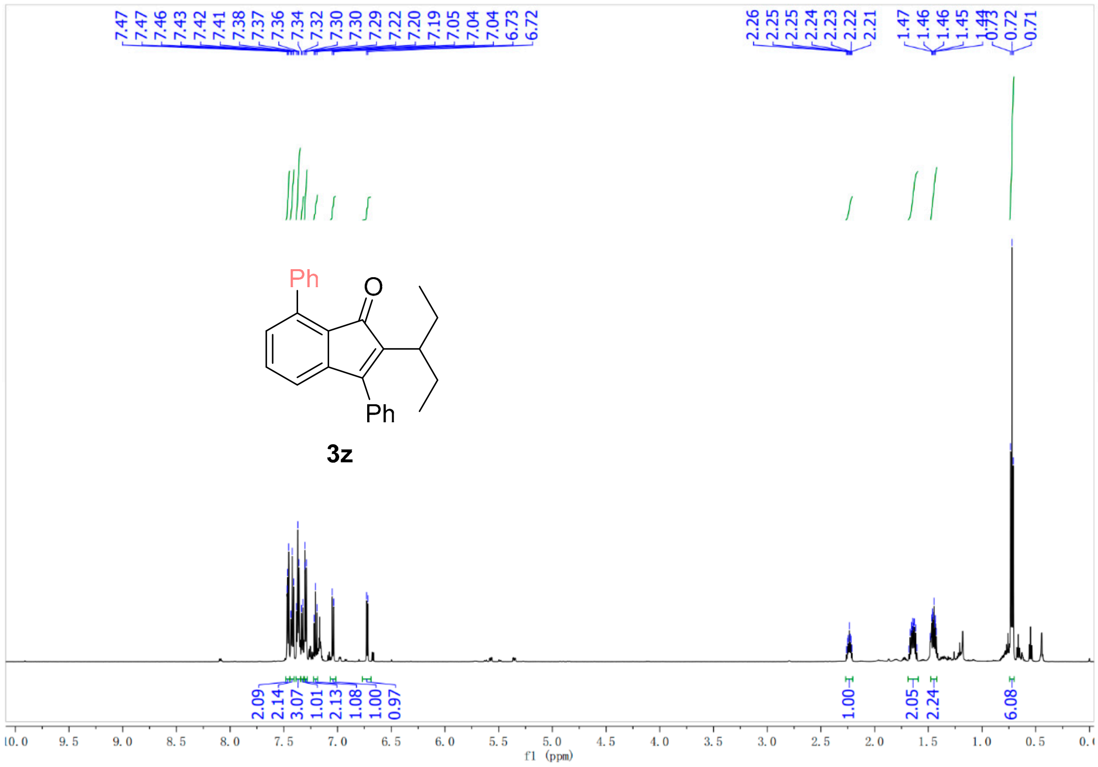

^{13}C NMR of **3z** in $CDCl_3$ (151 MHz, $CDCl_3$)

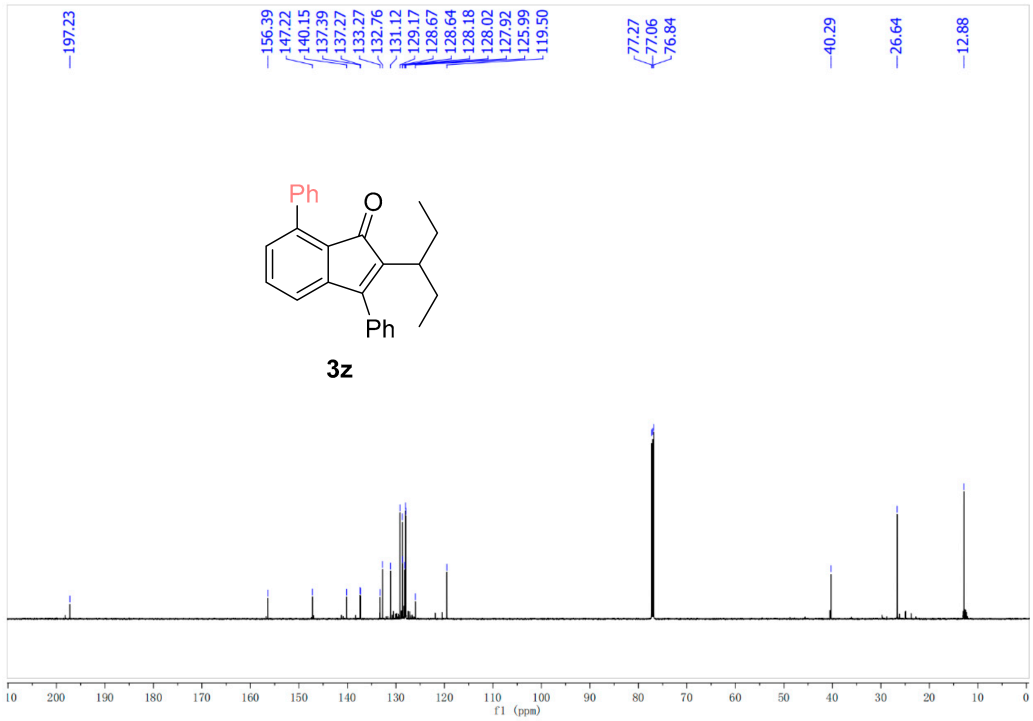

Supplement: Supplementary file 1 [file molecules-29-00458-s001.zip › molecules-2816533-supplementary.pdf]
